# Supplementary material for: SCFFBW7-mediated degradation of Brg1 suppresses gastric cancer metastasis
Source: Nat Commun. 2018 Sep 3;9:3569. doi: 10.1038/s41467-018-06038-y (PMC6120942; doi:10.1038/s41467-018-06038-y)

Supplementary Information

SCF^{FBW7}-mediated Degradation of Brg1 Suppresses Gastric Cancer Metastasis

Huang et al.

Supplementary Figure 1

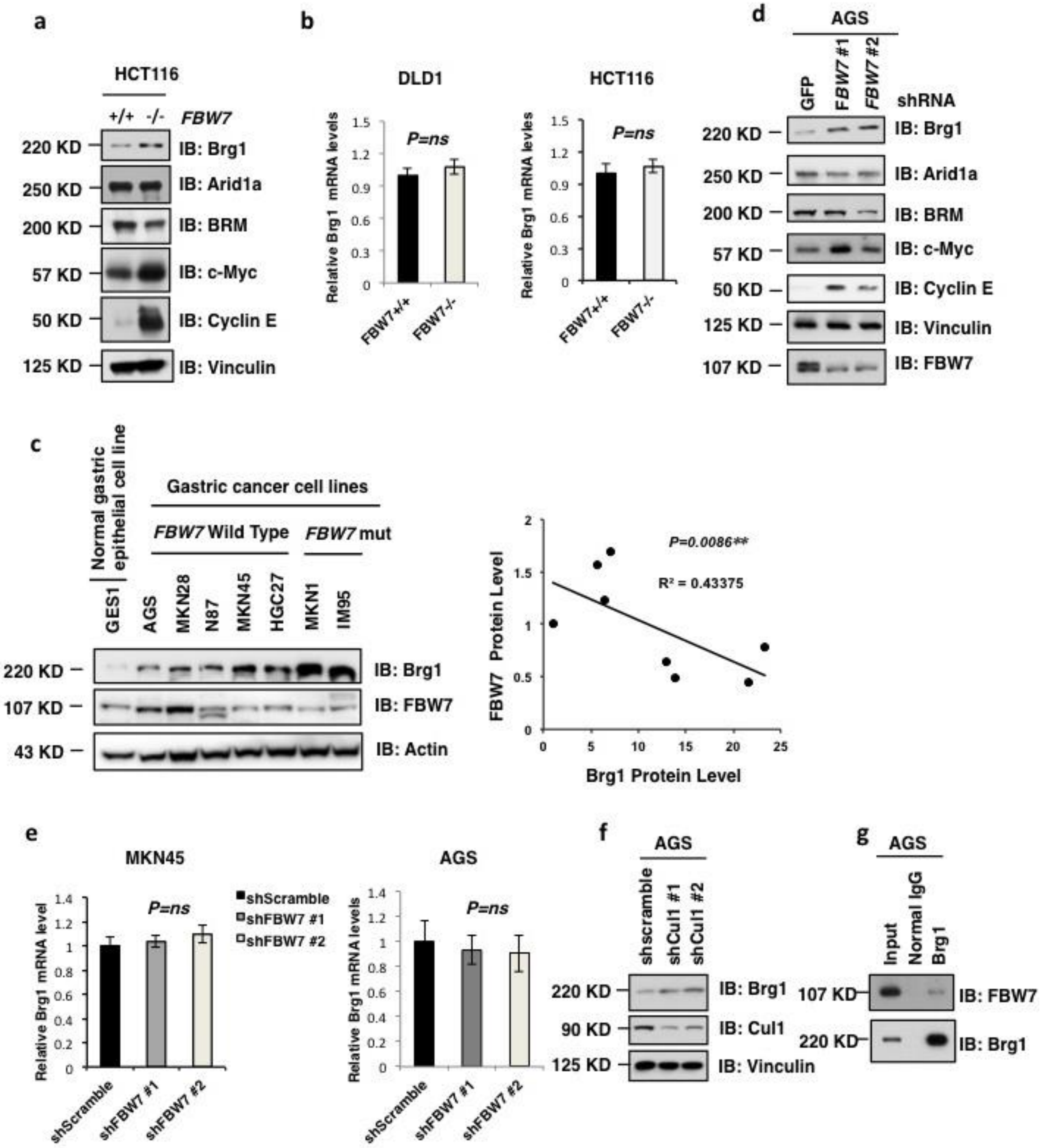

Supplementary Figure 1: FBW7 negatively regulates Brg1.

- **(a)** Immunoblot (IB) analysis of whole cell lysates (WCL) derived from wild-type (*FBW7*$^{+/+}$) and *FBW7*$^{-/-}$ HCT116 cells.
- **(b)** Real-time PCR analysis of Brg1 gene expression in WT and *FBW7*$^{-/-}$ DLD1 and HCT116 cells.
- **(c)** IB analysis of WCL derived from different gastric cancer cell lines. GES1 is a normal gastric epithelial cell line as a negative control.
- **(d)** IB analysis of WCLs derived from AGS cells transfected with the indicated *FBW7* shRNA lentivirus.
- **(e)** Real-time PCR analysis of Brg1 gene expression in MKN45 and AGS cells transfected with the indicated shRNA.
- **(f)** IB analysis of WCLs derived from AGS cells transfected with the indicated *Cullin1* shRNA lentiviruses.
- **(g)** Co-IP experiments in AGS cells were performed using anti-Brg1 antibody (sc-17796, Santa Cruz). Mouse IgG was used as a control.

Supplementary Figure 2

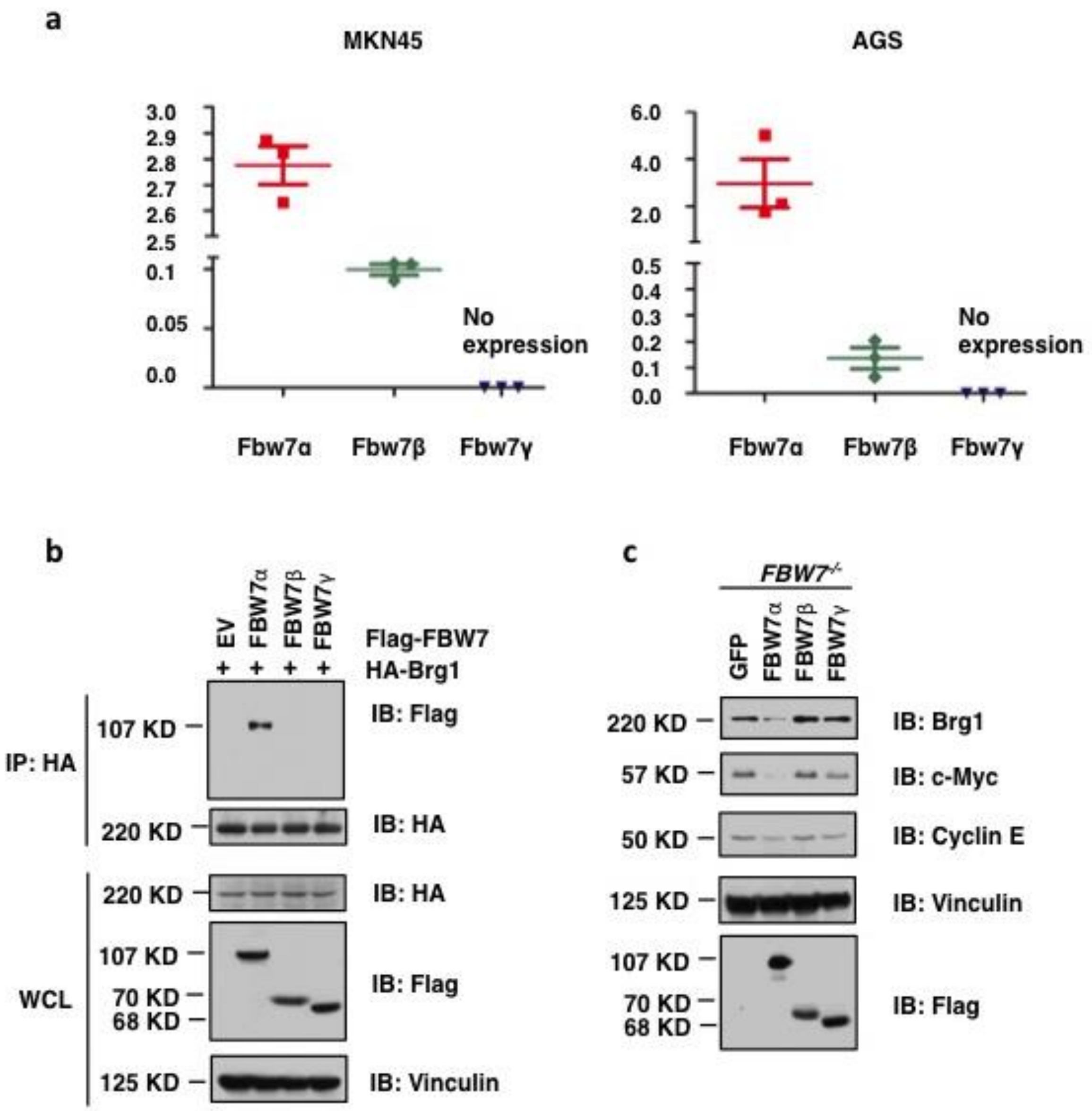

Supplementary Figure 2: FBW7α is the dominant isoform of FBW7 expressed in gastric cancer cells that regulates Brg1.

- **(a)** Real-time PCR analysis of three FBW7 isoforms (α, β, γ) gene expression in MKN45, and AGS gastric cancer cells. Specific Primers are designed: FBW7α Forward: 5-CGGAGTCTCCCAAACCTGAC-3, Reverse: 5-GGCGCGGTACTCCTCTTTT-3; FBW7β Forward: 5-AGTCTGCATTGCTGAATCCTG-3, Reverse: 5-ACTATGCCC TGTCAAAGCCT; FBW7γ Forward: 5-GGTCAGGACATTTGGTAGGG-3, Reverse: 5-GACAAAAAGGGAGGCCTTGG-3.
- **(b)** IB analysis of WCLs and IPs derived from MKN45 cells transfected with HA-Brg1 together with three Flag-FBW7 isoforms (α, β, γ) plasmids.
- **(c)** IB analysis of WCLs derived from *FBW7*$^{-/-}$ DLD1 cells transfected with three Flag-FBW7 isoforms (α, β, γ) plasmids.

Supplementary Figure 3

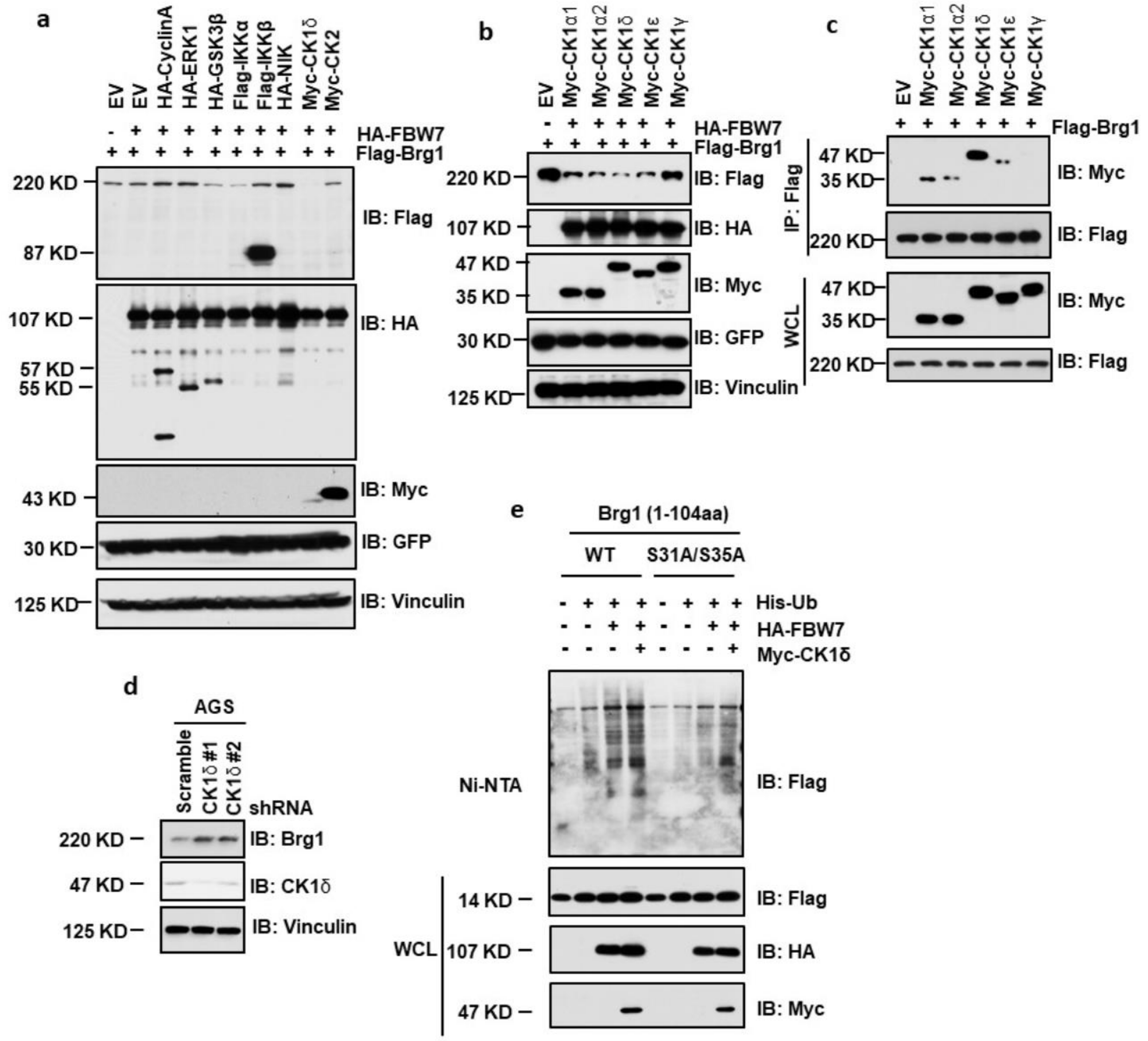

Supplementary Figure 3: CK1δ is the crucial upstream kinase of Brg1 and promotes the FBW7-mediated Brg1 ubiquitination.

- **(a)** IB analysis of WCL derived from 293T cells transfected with the Flag-Brg1 and HA-FBW7 plasmids in the presence of indicated constructs. A plasmid encoding GFP was used as a negative control for transfection efficiency.
- **(b)** IB analysis of 293T cells transfected with the Flag-Brg1 and HA-FBW7 plasmids in the presence of the indicated constructs.
- **(c)** IB analysis of WCLs and IPs derived from 293T cells transfected with Flag-Brg1 together with the indicated Myc-tagged CK1 family vectors.
- **(d)** IB analysis of WCLs derived from AGS cells infected with the indicated lentiviral shRNA-CK1δ constructs.
- **(e)** IB analysis of WCLs and His tag pull-down of 293 cells transfected with indicated Flag-Brg1(1-104aa) constructs together with the HA-FBW7, Myc–CK1δ and His-Ub plasmids.

Supplementary Figure 4

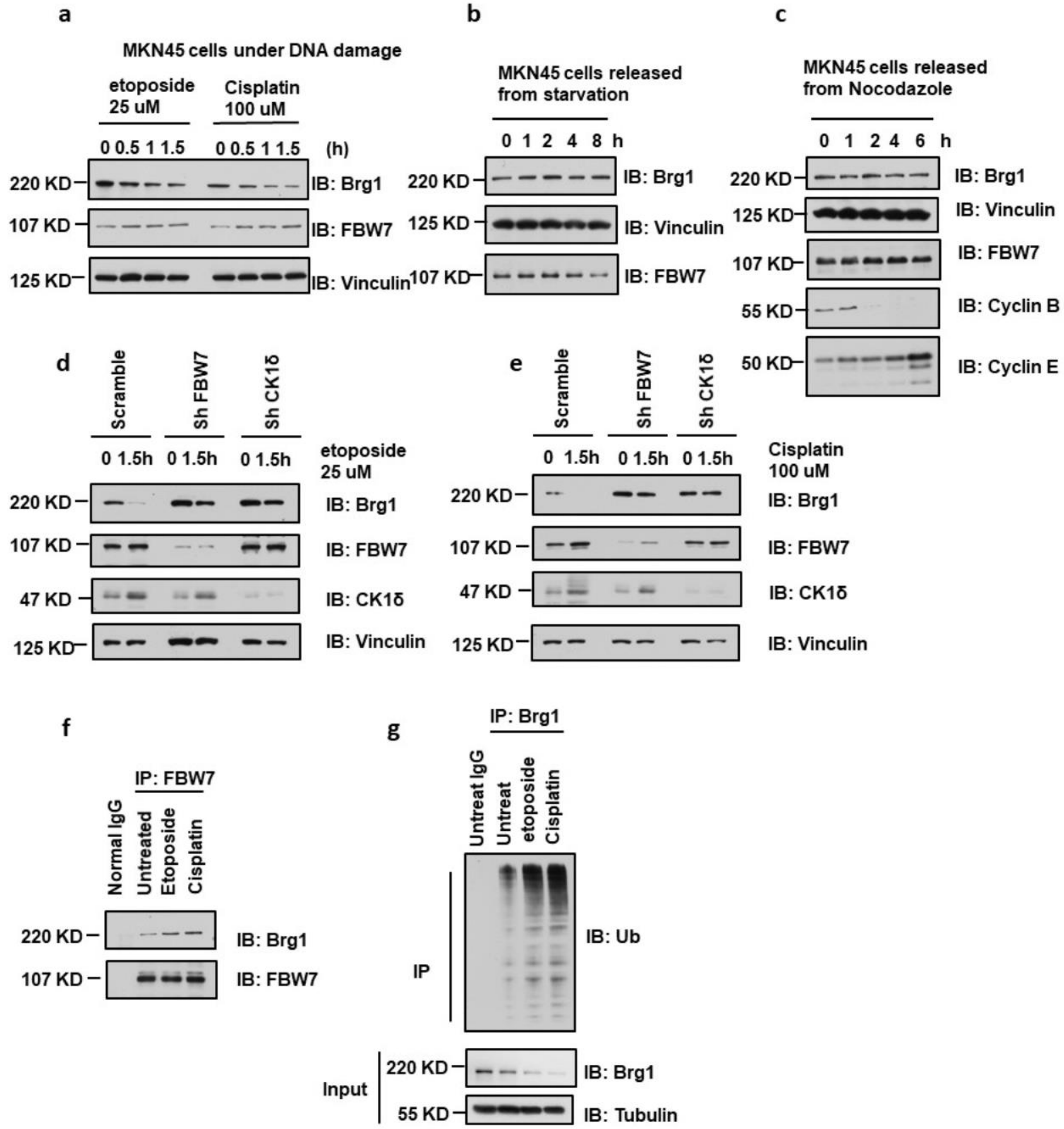

Supplementary Figure 4: Brg1 degradation and ubiquitination mediated by FBW7/CK1δ is triggered under DNA damage conditions.

- **(a)** MKN45 cells were treated with 25 uM etoposide or 100 uM Cisplatin. At the indicated time points, WCLs were prepared and immunoblots were probed with the indicated antibodies.
- **(b)** IB analysis of MKN45 cells treated by serum starvation overnight and followed by serum re-addition for the indicated times.
- **(c)** IB analysis of MKN45 cells synchronized by growth in nocodazole and then released for the indicated periods of time.
- **(d)** IB analysis of WCLs derived from MKN45 cells treated with 25 uM etoposide.
- **(e)** IB analysis of WCLs derived from MKN45 cells treated with 100 uM Cisplatin.
- **(f)** Endogenous co-IPs using FBW7 antibody and IB analysis of WCLs derived from MKN45 cells treated with 25 uM etoposide, or 100 uM Cisplatin for 1.5h.
- **(g)** Endogenous co-IPs using Brg1 antibody and IB analysis of WCLs derived from MKN45 cells treated with 25 uM etoposide, or 100 uM Cisplatin for 1.5h.

Supplementary Figure 5

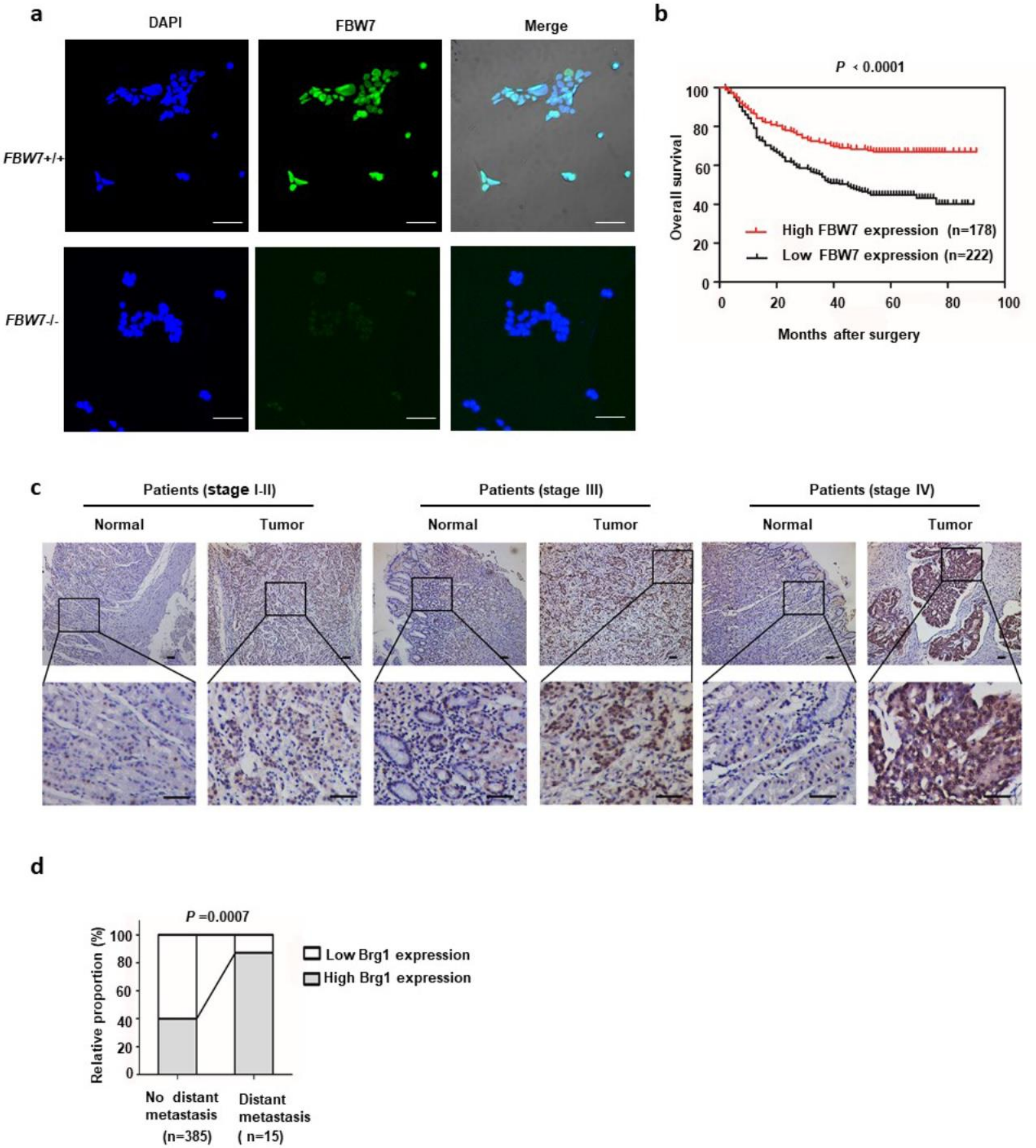

Supplementary Figure 5: Brg1 overexpression correlates with gastric tumor progression.

- **(a)** The immunofluorescence of FBW7 (ab109617, Abcam) in wild-type (*FBW7*$^{+/+}$) and *FBW7*$^{-/-}$ DLD1 cells. Scale Bar, 100 μm.
- **(b)** The association of FBW7 expression with overall survival was examined by Kaplan-Meier analysis in gastric cancer patients from the Zhongshan cohort.
- **(c)** Representative images of Brg1 expression in tumor sections and adjacent non-tumor tissues according to TNM stage. Scale Bar, 100 μm.
- **(d)** High Brg1 expression positively correlates with distant metastasis in gastric cancer. Their correlation was analyzed by Spearman rank correlation test.

Supplementary Figure 6

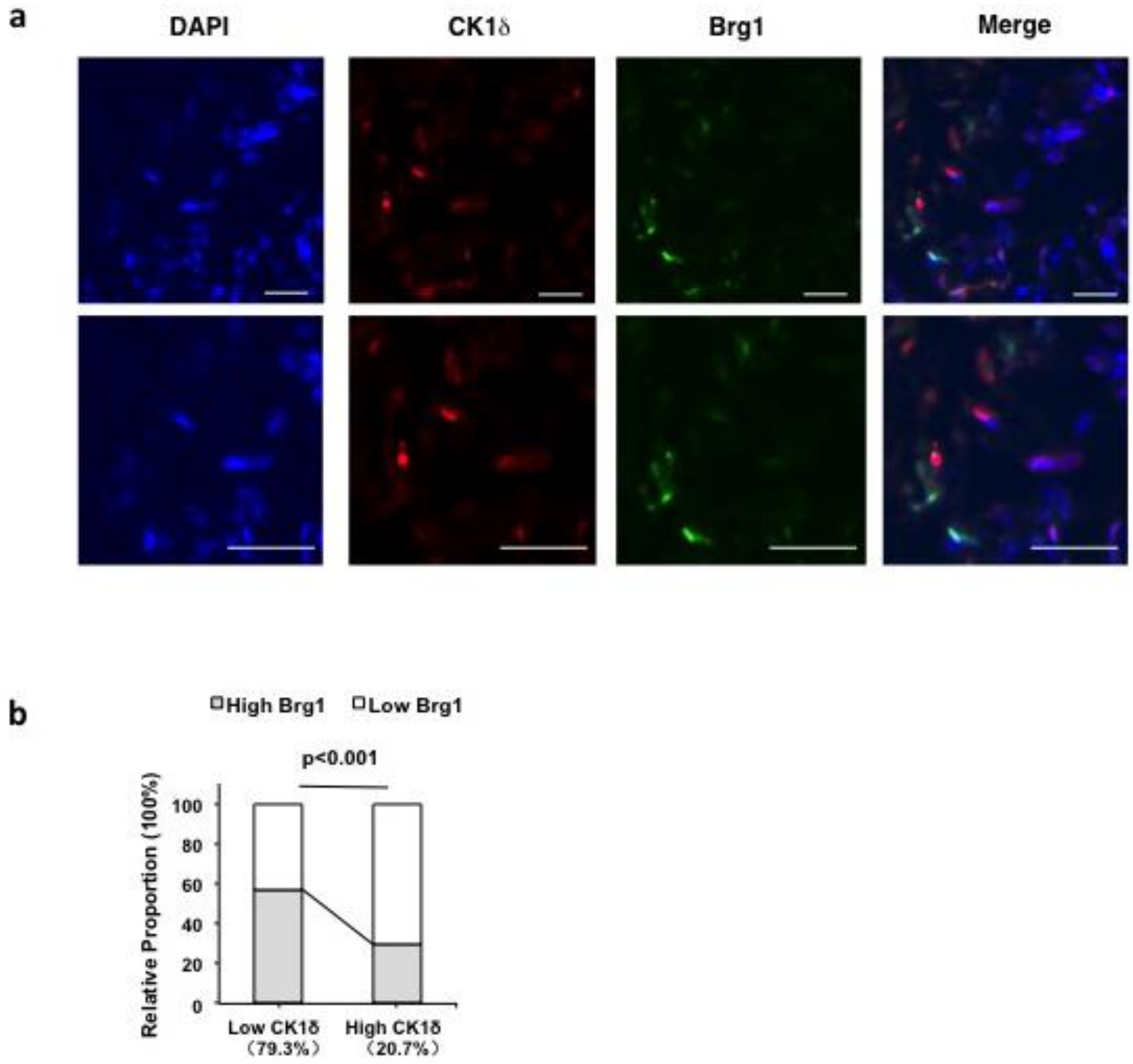

Supplementary Figure 6: The Brg1 level is inversely correlated with the activation of CK1δ in gastric cancer samples.

(a) Representative immunofluorescence (IF) images of Brg1(sc-17796, Santa Cruz) and CK1δ (14388-1-AP, Proteintech) in tumor sections. Scale Bar, 100 μm.

(b) The Brg1 expression inversely correlates with CK1δ activation in gastric cancer. Their correlation was analyzed by Spearman rank correlation test.

Supplementary Figure 7

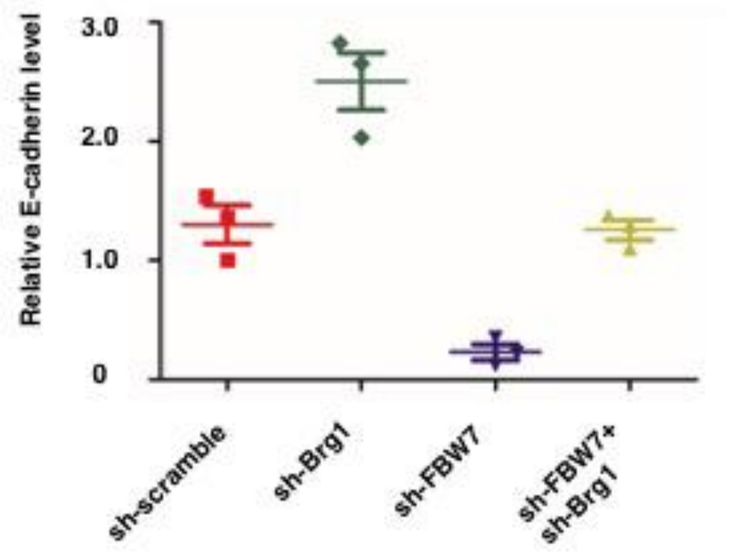

Supplementary Figure 7: The FBW7-Brg1 axis regulates the mRNA expression of E-cadherin.

Real-time PCR analysis of E-cadherin gene expression in MKN45 cells transfected with the indicated shRNA.

Supplementary Figure 8

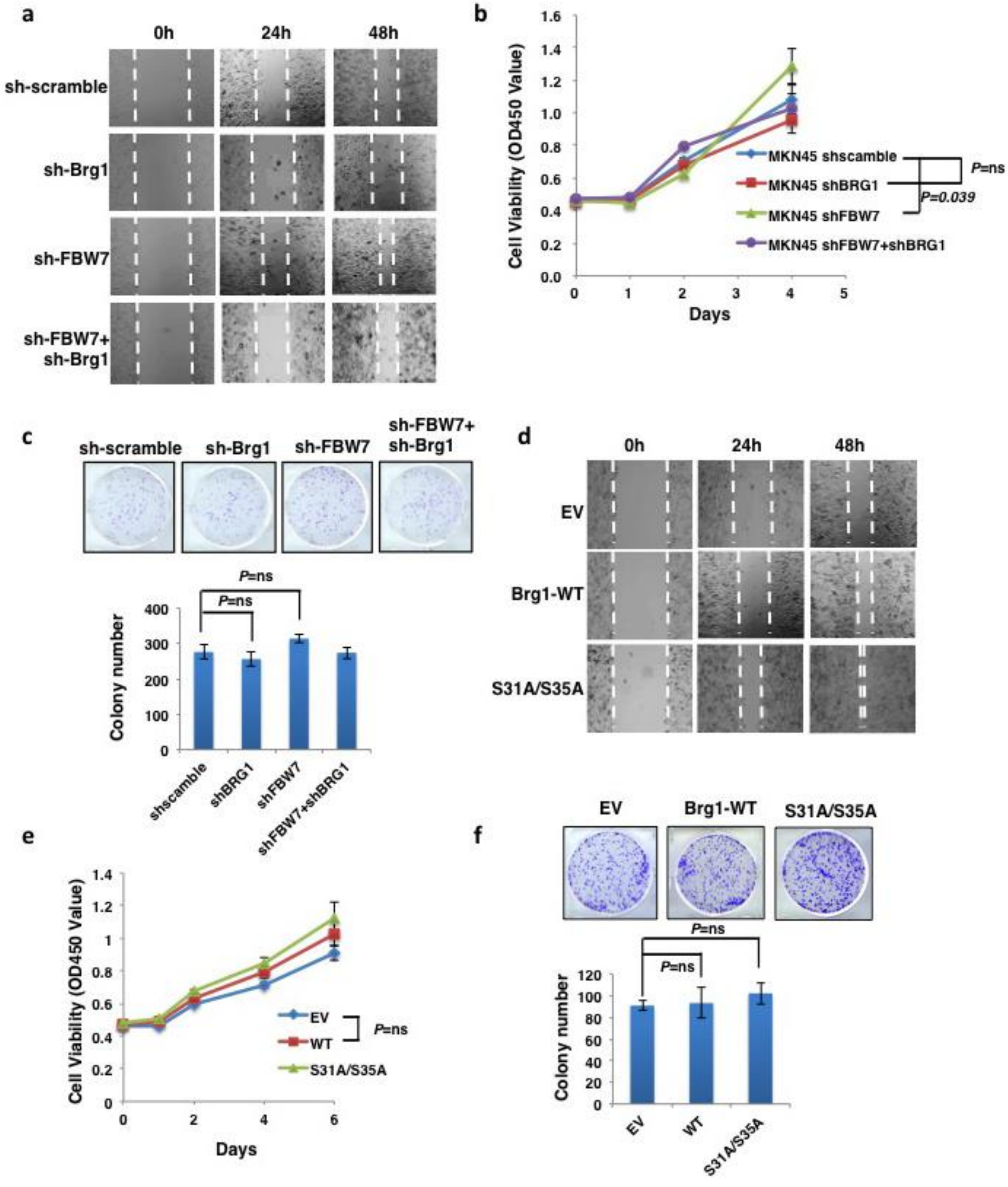

Supplementary Figure 8: Brg1 has no significant effect on the cell proliferation of gastric cancer cells.

- **(a)** *In vitro* scratch assays were performed with cell lines generated in Fig 4a. Cells were seeded on 60 mm dishes and scratched on the surface with a pipette tip. Representative photographs at time points 0, 24 and 48 hr were imaged after the scratch.
- **(b)** Cell growth curves of MKN45 cells treated with the indicated shRNA lentiviruses were plotted.
- **(c)** Colony formation assays of MKN45 cells treated with the indicated shRNA lentiviruses were performed. The quantification of the colonies was shown in the bottom. Data was presented as mean ± SD.
- **(d)** *In vitro* scratch assay was performed with cell lines in Fig 4h.
- **(e)** Cell growth curves of AGS cells transfected with the indicated Flag-Brg1 vectors were plotted.
- **(f)** Colony formation assays of AGS cells transfected with the indicated Flag-Brg1 vectors. The quantification of the colonies was shown in the bottom. Data was presented as mean ± SD.

Supplementary Figure 9

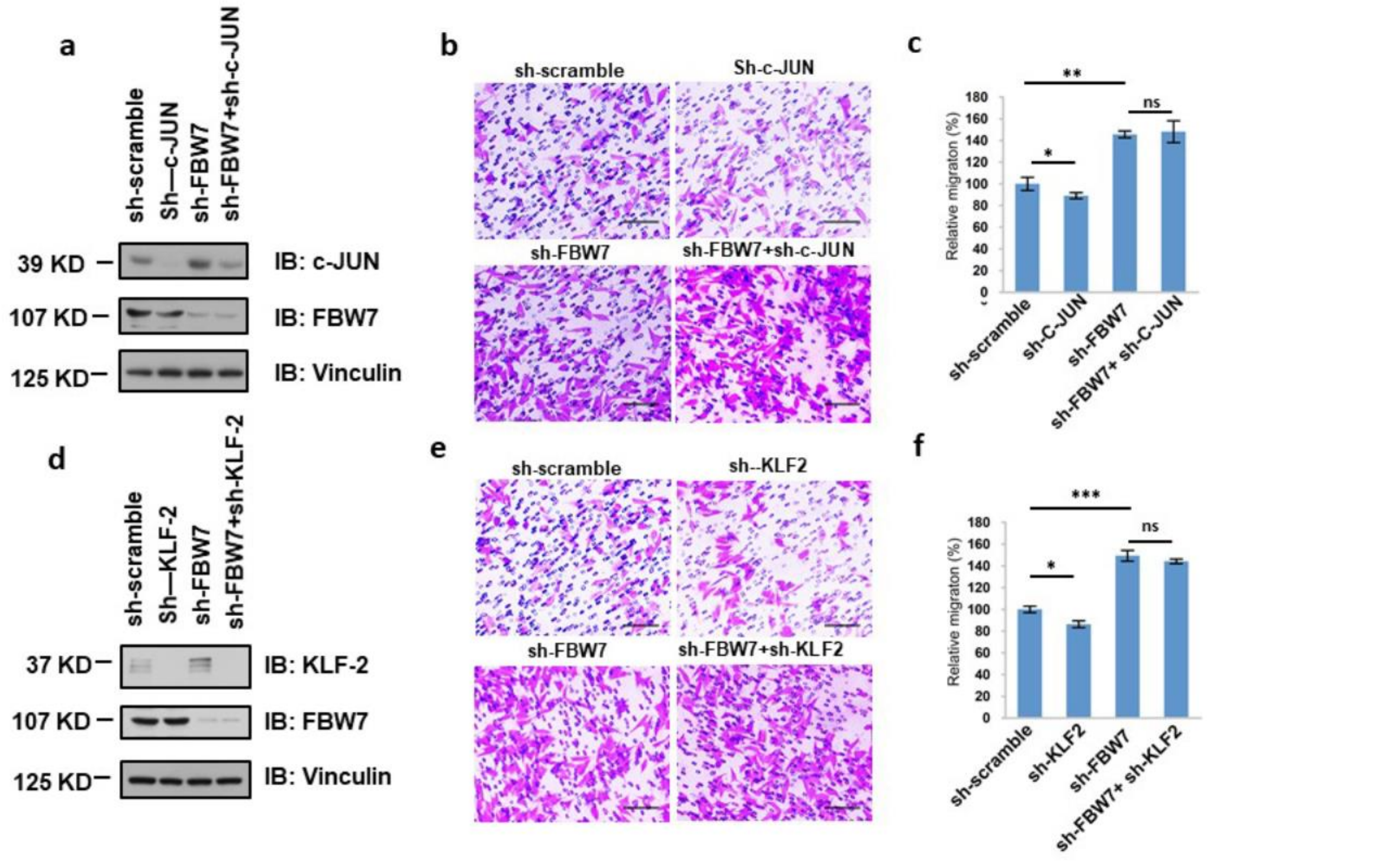

Supplementary Figure 9: The knockdown of *c-Jun* or *KLF2* does not attenuate the increased cell migration induced by *FBW7* knockdown in gastric cancer cell line MKN45.

(a) IB analysis of WCLs derived from AGS cells infected with the indicated lentiviral shRNA constructs.

(b-c) Representative images of migrated AGS cells infected with the indicated lentiviral shRNA constructs in a transwell assay **(b)** and quantification of migrated cells in **(c)**. Data were shown as mean ± SD of three independent experiments. *p < 0.05, **p < 0.01, Student's t test. Scale Bar, 100 μm.

(d) IB analysis of WCLs derived from AGS cells infected with the indicated lentiviral shRNA constructs.

(e-f) Representative images of migrated AGS cells infected with the indicated lentiviral shRNA constructs in a transwell assay **(e)** and quantification of migrated cells in **(f)**. Data were shown as mean ± SD of three independent experiments. *p < 0.05, ***p < 0.001,Student's t test. Scale Bar, 100 μm.

Supplementary Figure 10

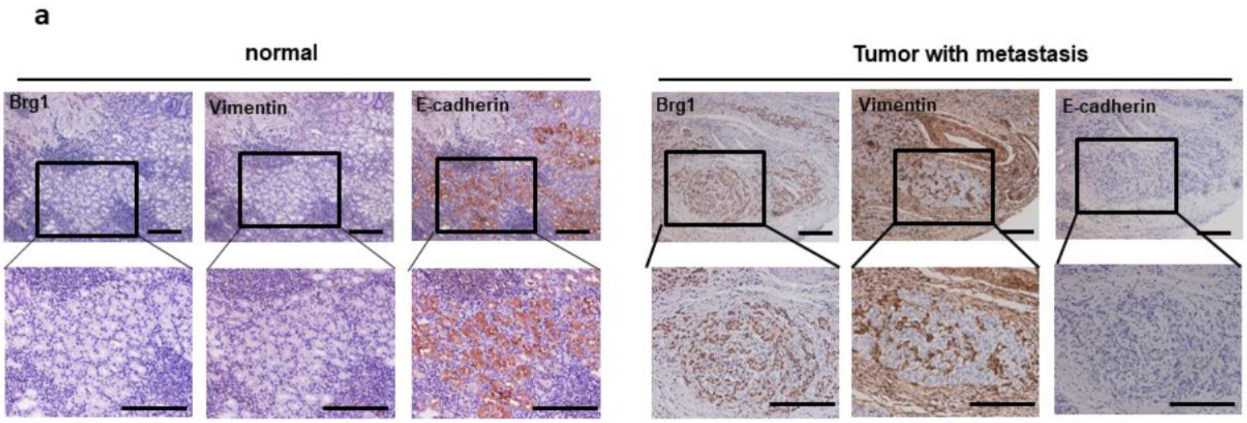

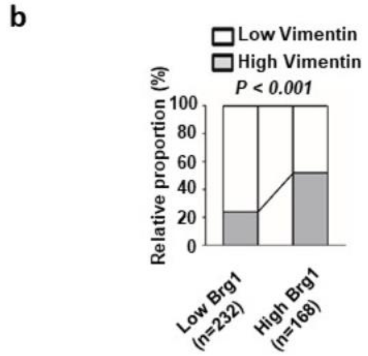

Supplementary Figure 10: Brg1 expression correlates with EMT in gastric cancer.

(a) Representative images of Brg1, Vimentin and E-cadherin expression in tumor sections. Scale Bar, 100 μm.

(b) High Brg1 expression positively correlates with Vimentin expression in gastric cancer. Their correlation was analyzed by Spearman rank correlation test.

Supplementary Figure 11

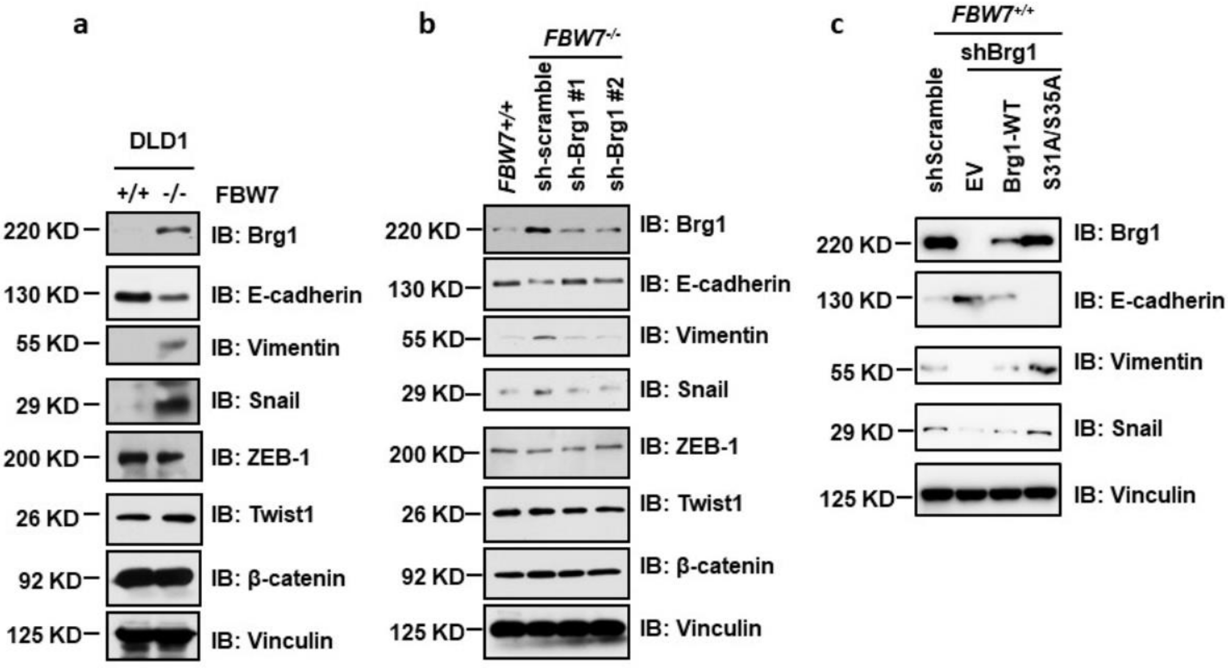

Supplementary Figure 11: Brg1 drives EMT transcriptional program.

- **(a)** IB analysis of WCLs derived from WT (*FBW7*$^{+/+}$) and *FBW7*$^{-/-}$ DLD1 cells.
- **(b)** IB analysis of WCLs derived from WT and *FBW7*$^{-/-}$ DLD1 cells infected with the indicated shRNA lentiviruses.
- **(c)** IB analysis of WCLs derived from WT DLD1 cells transfected with the indicated shRNA lentivirus and Flag-Brg1 constructs.

Supplementary Figure 12

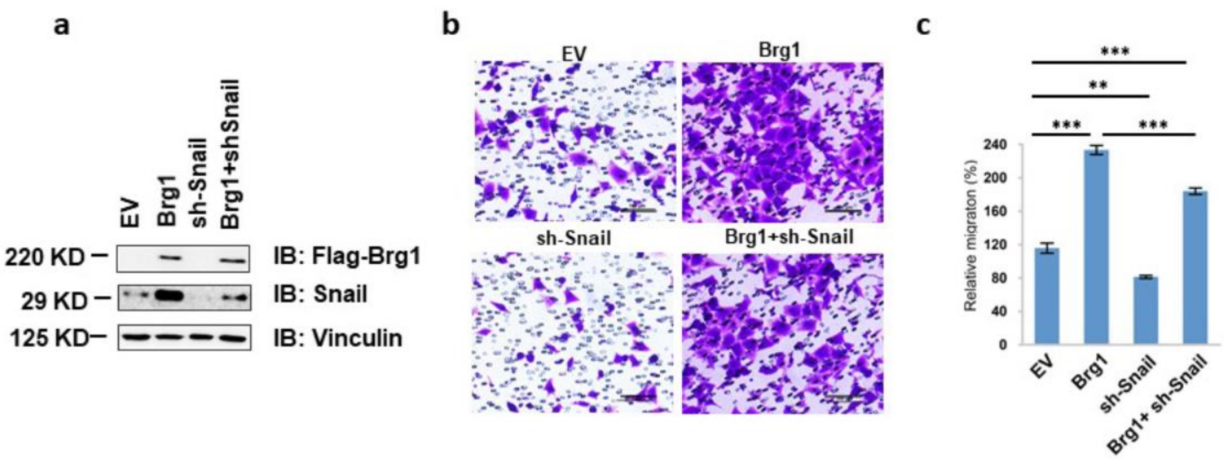

Supplementary Figure 12: Brg1 drives EMT in part through regulating Snail.

(a) IB analysis of WCL derived from AGS cells transfected with indicated plasmid or lentiviral shRNA in the presence of indicated constructs.

(b-c) Representative images of migrated AGS cells infected with indicated plasmid or lentiviral shRNA constructs in a transwell assay **(b)** and quantification of migrated cells in **(c)**. Data were shown as mean ± SD of three independent experiments. $^{**}p < 0.01$, $^{***}p < 0.001$, Student's t test.

Supplementary Figure 13

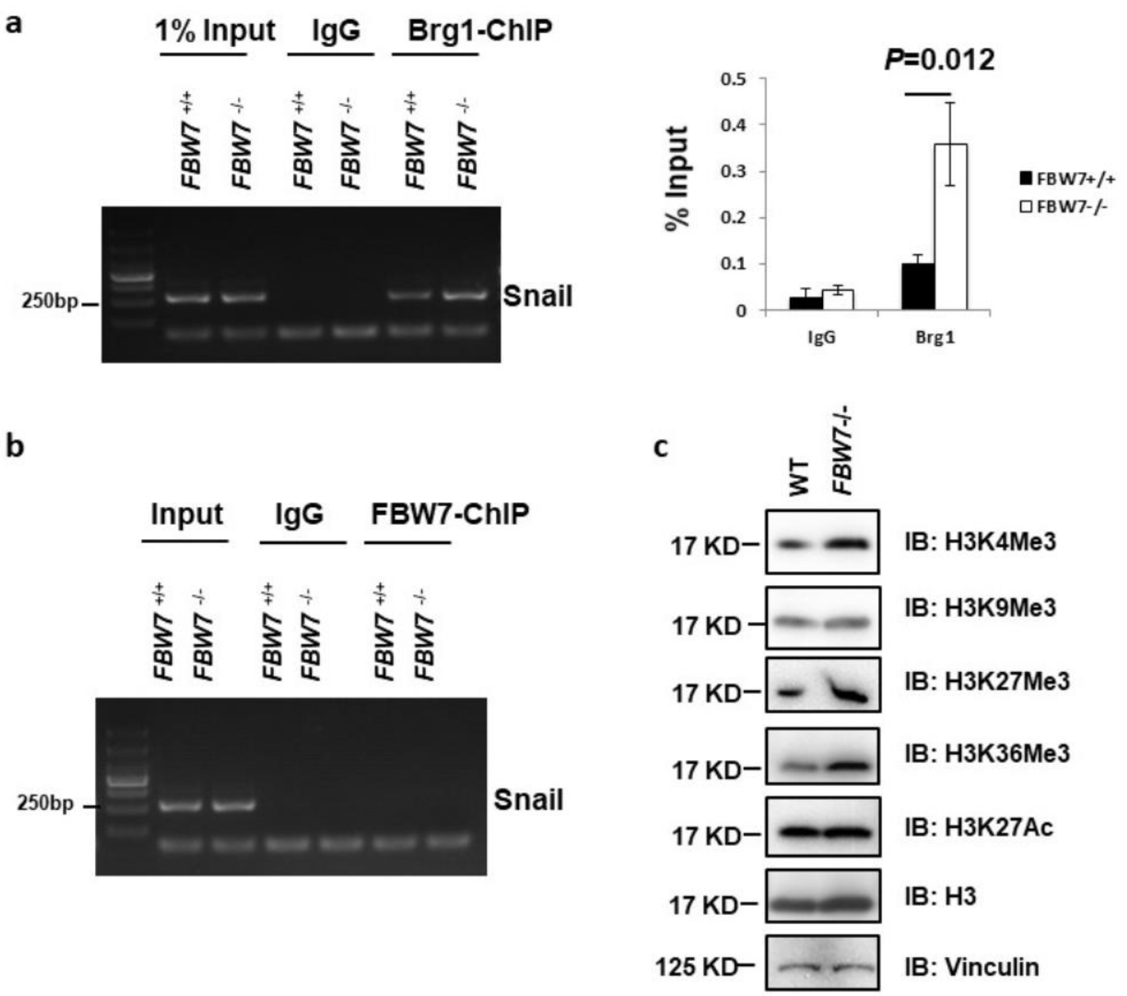

Supplementary Figure 13: FBW7-Brg1 regulates histone modification.

- **(a)** ChIP assays with anti-Brg1 antibody were performed in WT (*FBW7*$^{+/+}$) and *FBW7*$^{-/-}$ DLD1 cells.
- **(b)** ChIP assays with anti-FBW7 antibody were performed in WT (*FBW7*$^{+/+}$) and *FBW7*$^{-/-}$ DLD1 cells.
- **(c)** IB analysis of WCLs derived from WT (*FBW7*$^{+/+}$) and *FBW7*$^{-/-}$ DLD1 cells.

Supplementary Table 1. Relation between intratumoral FBW7 expression and clinical characteristics of gastric cancer, related to Figure 3

Factor	Patients		FBW7 expression		
	No.	%	Low	High	P-value
**All patients**	400	100	222	178	
**Age (years)**					0.062
≤65	248	62	147	101	
＞65	152	38	75	77	
**Gender**					0.937
Female	134	34	74	60	
Male	266	66	148	118	
**Location**					0.479
Proximal	58	15	30	28	
Middle	87	21	53	34	
Distal	255	64	139	116	
**Size**					0.629
≦2.5cm	115	29	66	49	
> 2.5cm	285	71	156	129	
**Differentiation**					0.363
Well	75	19	47	28	
Moderately	114	29	60	54	
Poorly	211	52	115	96	
**Lauren classification**					**0.001**
Intestinal type	245	61	120	125	
Diffuse type	134	34	85	49	
Mixed type	21	5	17	4	
**T classification**					0.537
T1	61	15	29	32	
T2	36	10	19	17	
T3	120	30	68	52	
T4	183	45	106	77	
**N classification**					**0.026**
N0	111	28	50	61	
N1	68	17	41	27	
N2	75	19	39	36	
N3	146	36	92	54	
**Distant metastasis**					**0.013**
No	385	96	209	176	
Yes	15	4	13	2	
**TNM stage**					**0.034**
I	67	16	31	36	
II	96	24	51	45	
III	222	56	127	95	
IV	15	4	13	2	
**Vascular invasion**					0.583
No	282	71	159	123	
Yes	118	29	63	55	

Abbreviation: TNM=tumour node metastasis.

P-value＜0.05 marked in bold font shows statistical significant.

Supplementary Table 2. Relation between intratumoral Brg1 expression and clinical characteristics of gastric cancer, related to Figure 3

Factor	Patients		Brg1 expression		
	No.	%	Low	High	P-value
**All patients**	400	100	232	168	
**Age (years)**					0.199
⩽65	248	62	150	98	
＞65	152	38	82	70	
**Gender**					0.481
Female	134	34	81	53	
Male	266	66	151	115	
**Location**					0.971
Proximal	58	15	33	25	
Middle	87	21	50	37	
Distal	255	64	149	106	
**Size**					**0.003**
≦2.5cm	115	29	80	35	
> 2.5cm	285	71	152	133	
**Differentiation**					0.506
Well	75	19	47	28	
Moderately	114	29	68	46	
Poorly	211	52	117	94	
**Lauren classification**					0.384
Intestinal type	245	61	137	108	
Diffuse type	134	34	84	50	
Mixed type	21	5	11	10	
**T classification**					**< 0.001**
T1	61	15	55	6	
T2	36	10	21	15	
T3	120	30	69	51	
T4	183	45	87	96	
**N classification**					**< 0.001**
N0	111	28	85	26	
N1	68	17	42	26	
N2	75	19	44	31	
N3	146	36	61	85	
**Distant metastasis**					**< 0.001**
No	385	96	230	155	
Yes	15	4	2	13	
**TNM stage**					**< 0.001**
I	67	16	57	10	
II	96	24	64	32	
III	222	56	109	113	
IV	15	4	2	13	
**Vascular invasion**					**0.001**
No	282	71	178	104	
Yes	118	29	54	64	

Abbreviation: TNM=tumour node metastasis.

P-value＜0.05 marked in bold font shows statistical significant.

Supplementary Figure 14

Uncropped blots related to Figure 1a

Brg1

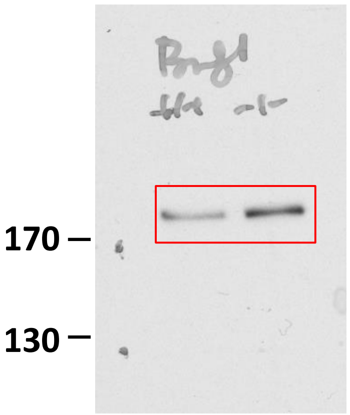

Arid1a

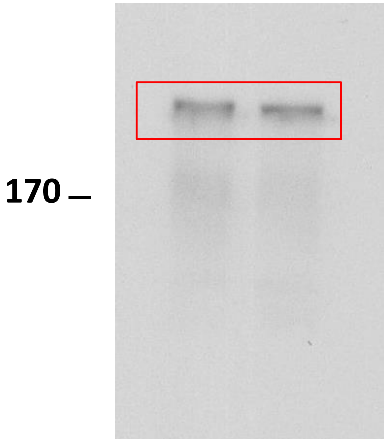

BRM

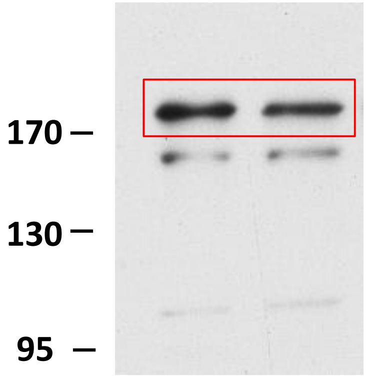

c-Myc

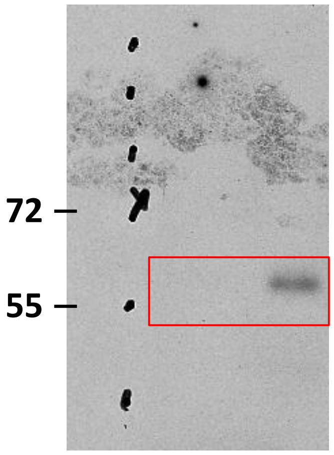

cyclinE

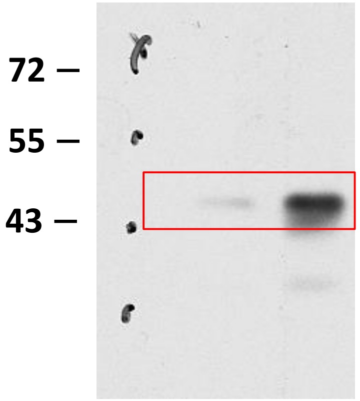

Vinculin

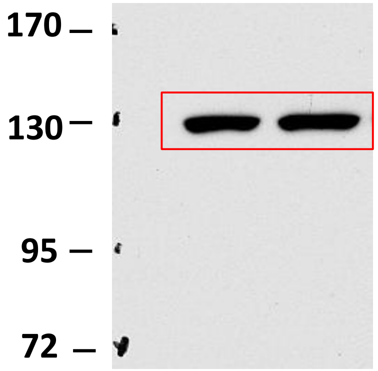

FBW7

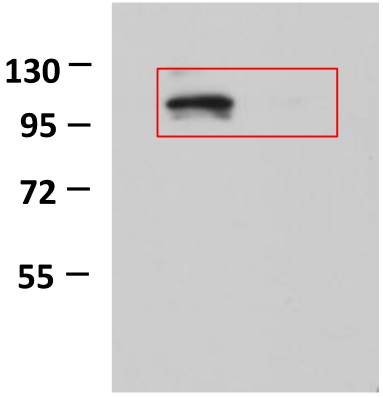

Uncropped blots related to Figure 1b and 1d

Fig1b-Brg-1

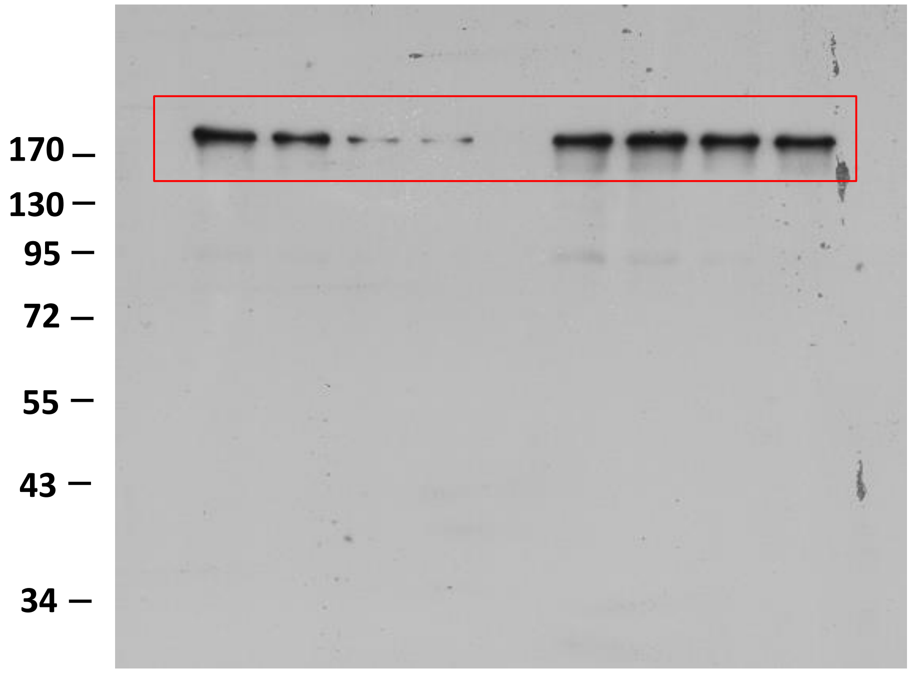

Fig1b-Vinculin

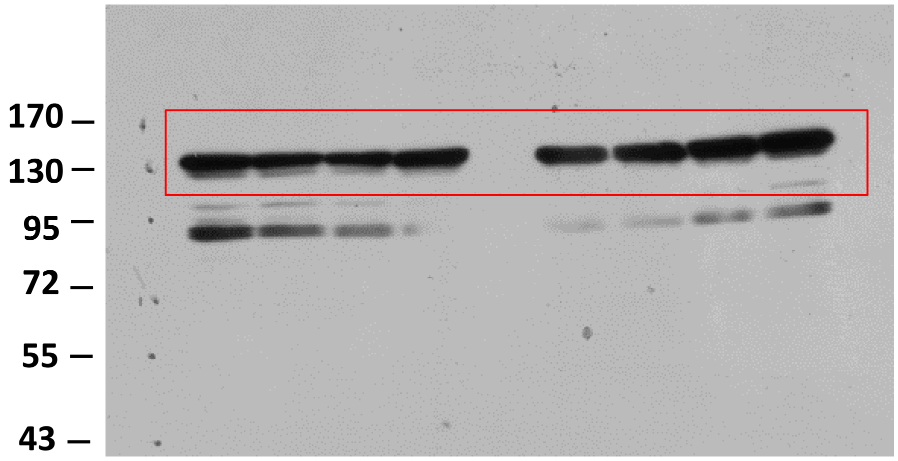

Fig1d-Brg1

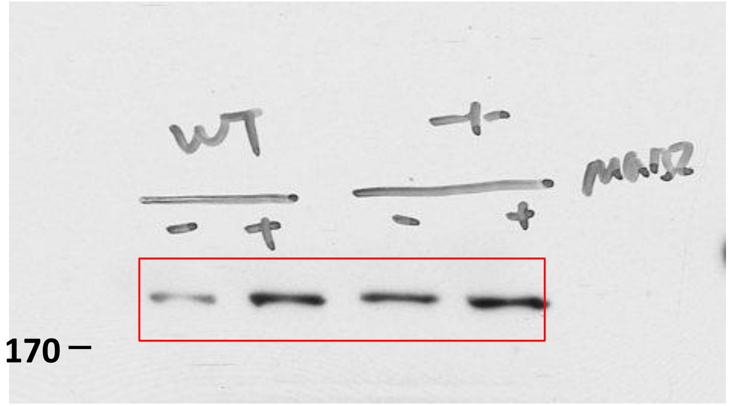

Fig1d-Vinculin

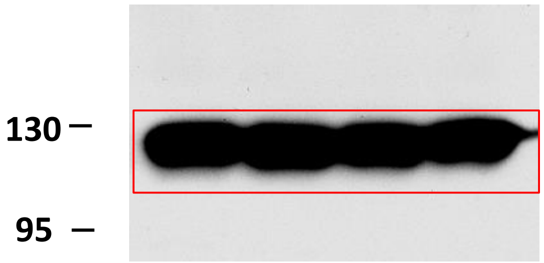

Uncropped blots related to Figure 1e

Brg1

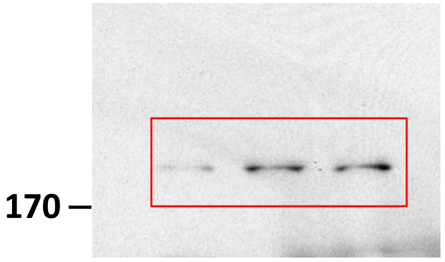

Arid1a

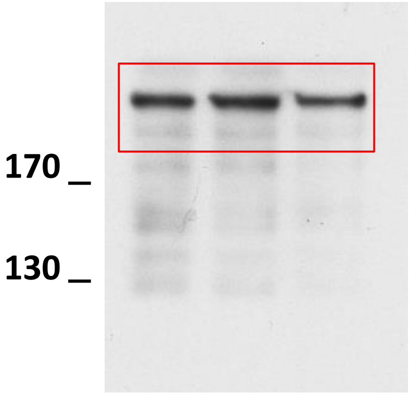

BRM

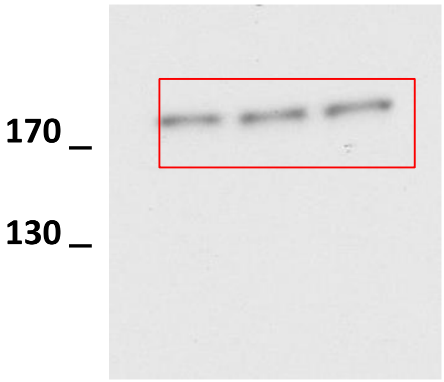

c-Myc

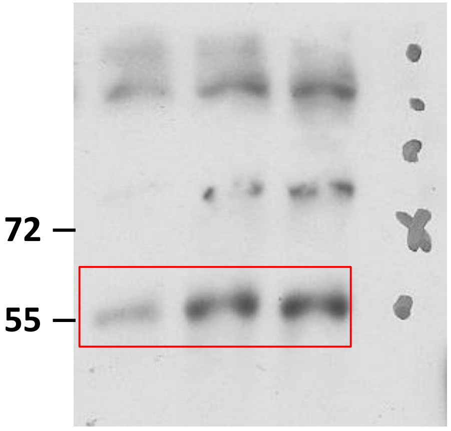

cyclinE

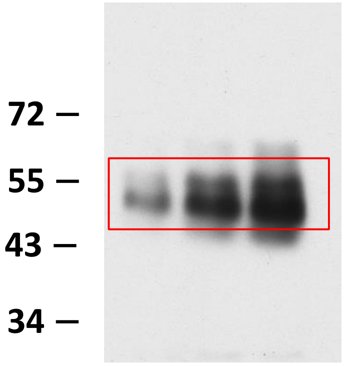

Vinculin

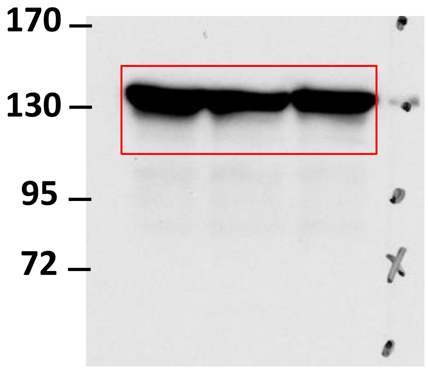

FBW7

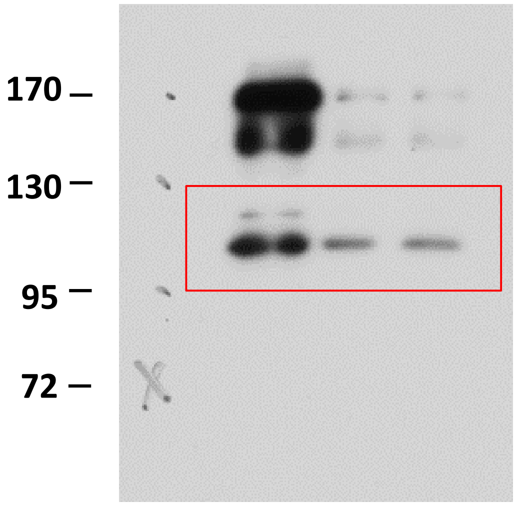

Fig1f-Brg-1

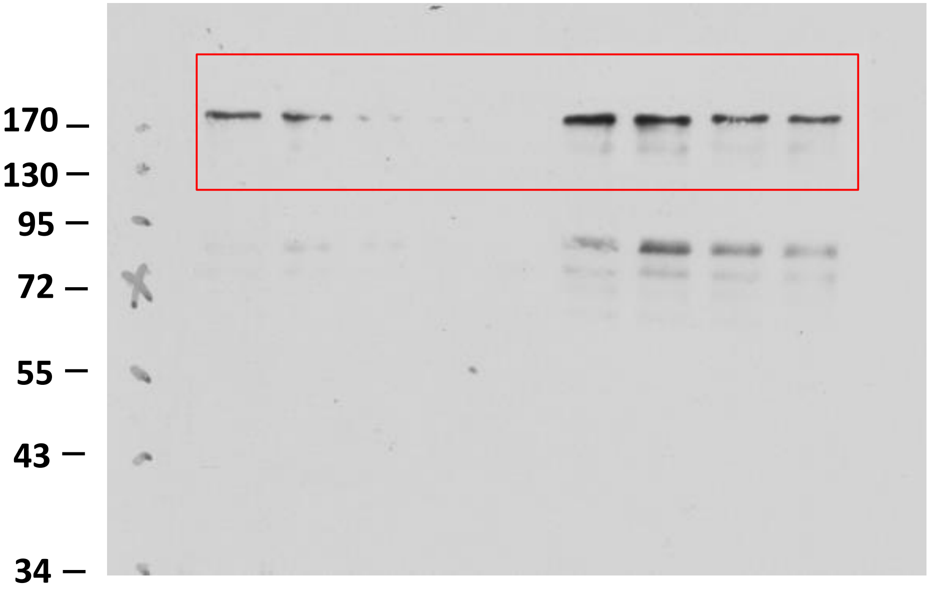

Fig1f-Vinculin

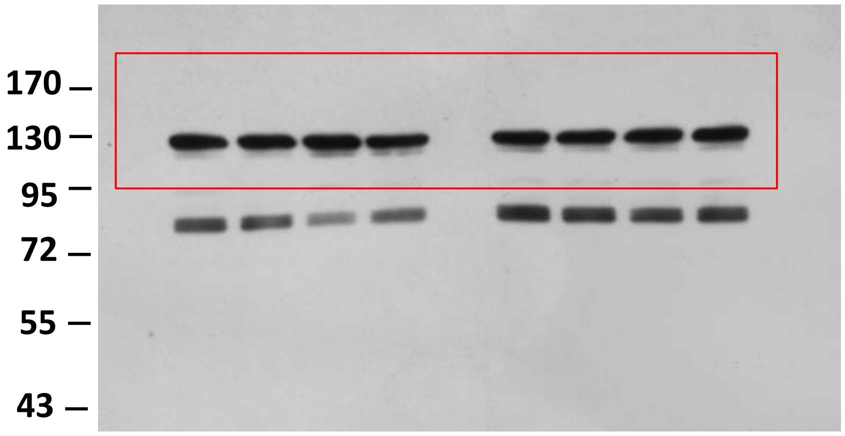

Fig1g-Myc (IP)

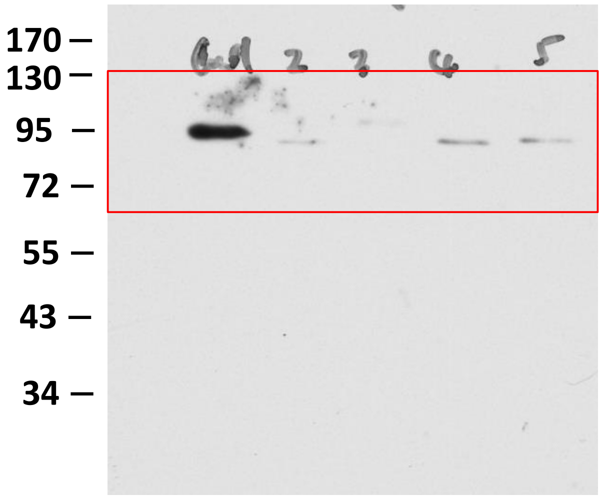

Fig1g-Flag (IP)

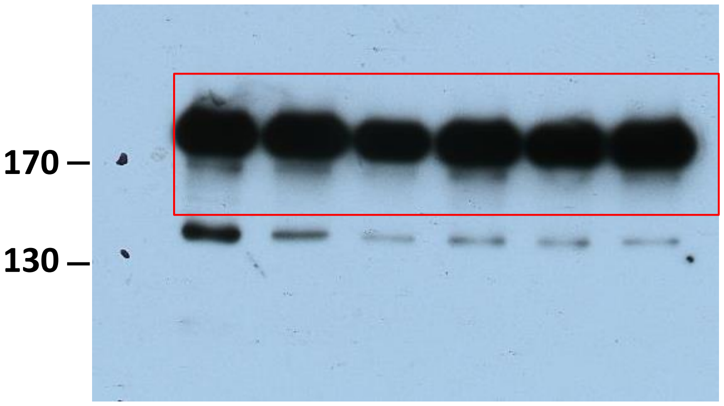

Fig1g-Myc (Lysates)

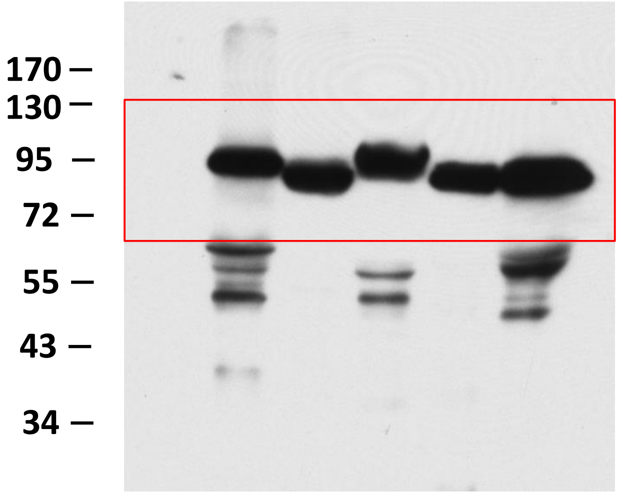

Fig1g-Flag (lysates)

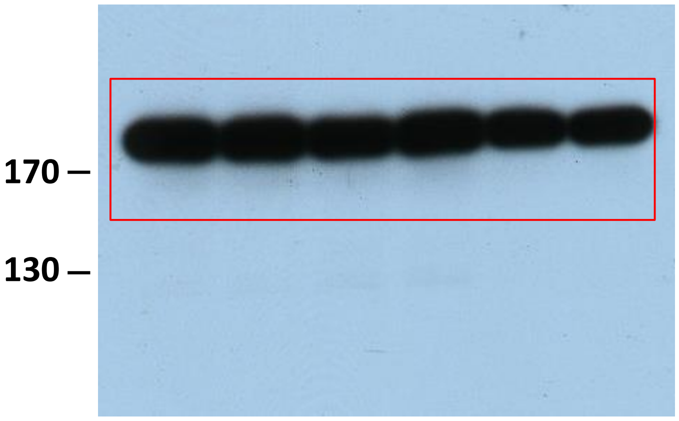

Uncropped blots related to Figure 1h

Brg1

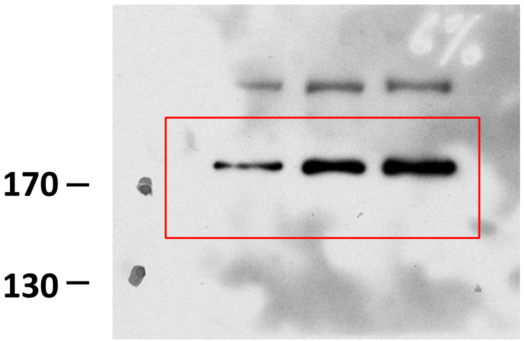

c-Myc

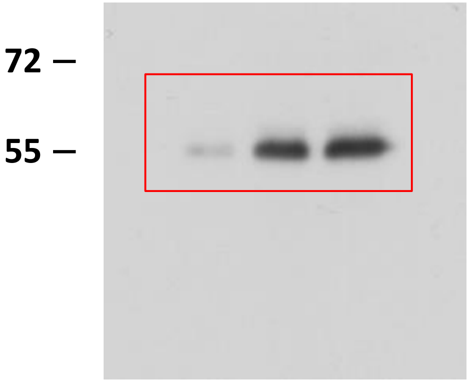

cyclinE

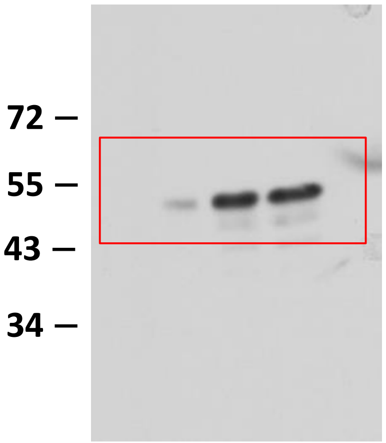

Cul1

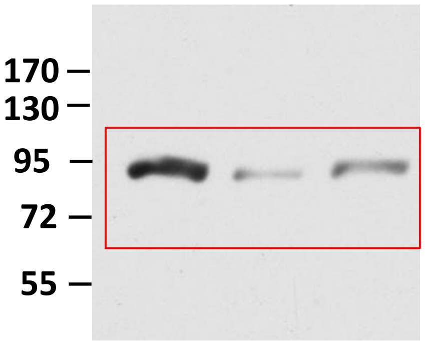

Vinculin

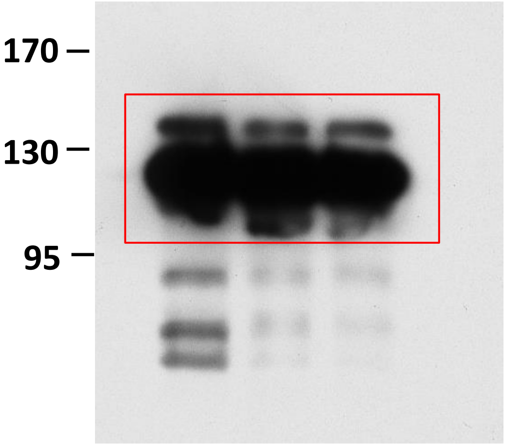

Uncropped blots related to Figure 1i and 1j

Fig1i-HA (IP)

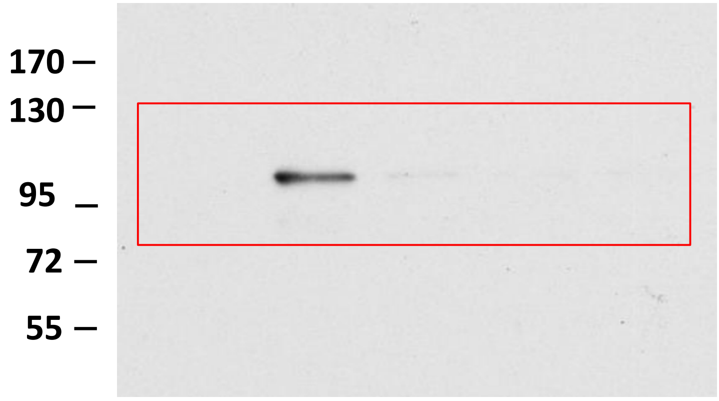

Fig1i-Flag (IP)

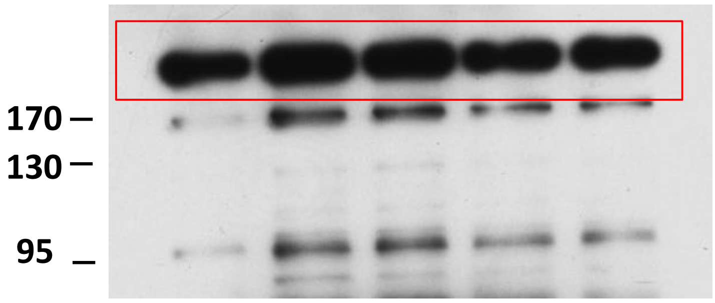

Fig1i-HA (lysates)

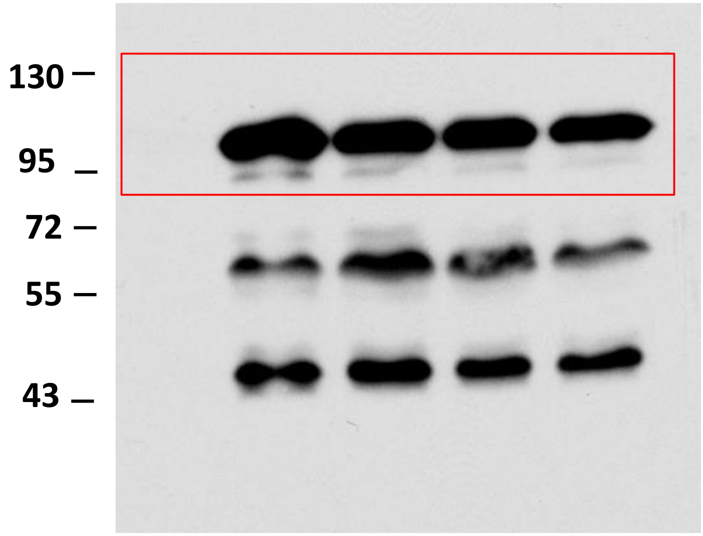

Fig1i-Flag (lysates)

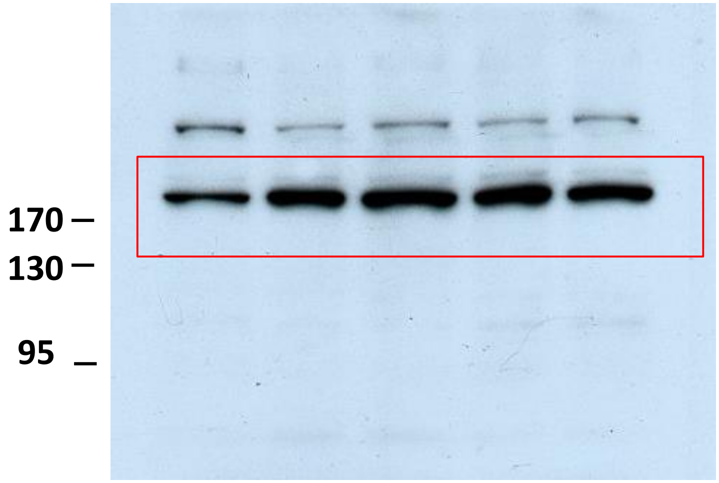

Fig1j-Brg1 (IP)

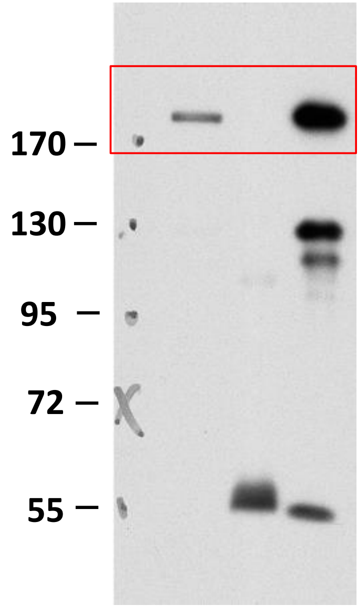

Fig1j-FBW7 (IP)

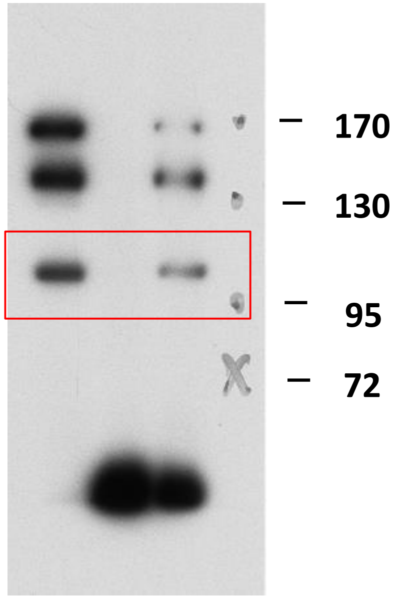

Fig1j-BAF155 (IP)

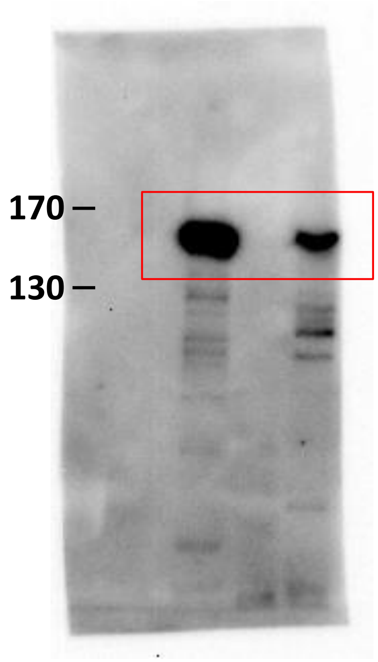

Uncropped blots related to Figure 1k

Brg1

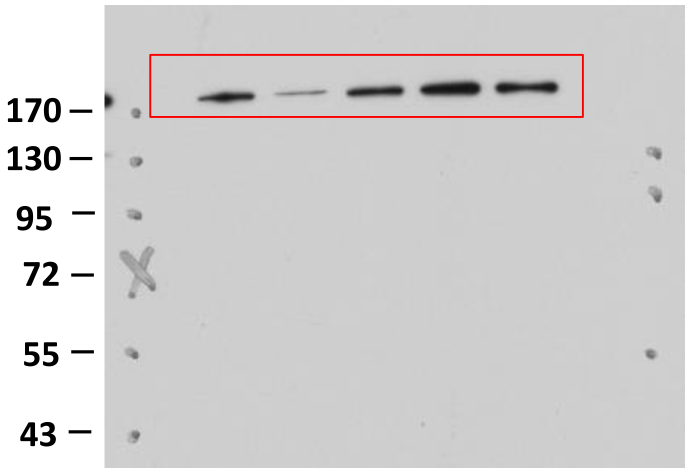

Arid1a

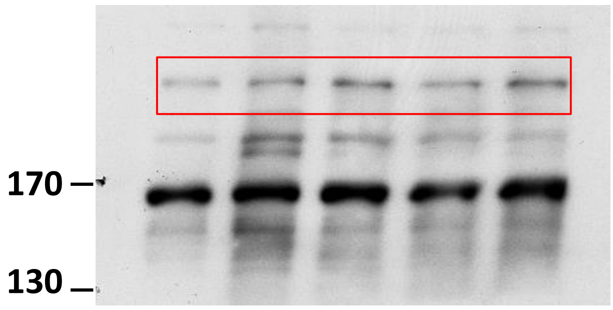

c-Myc

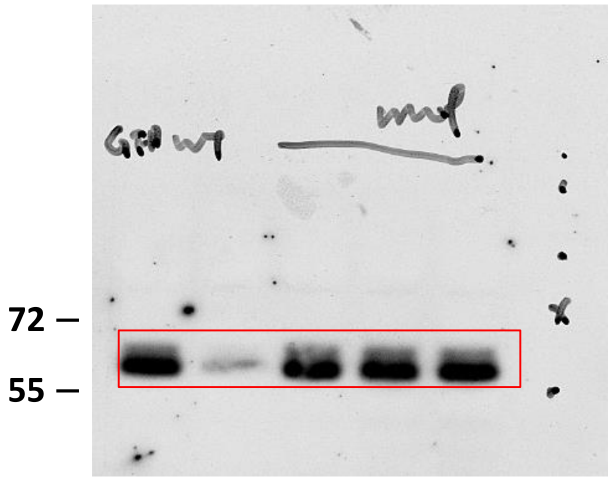

BRM

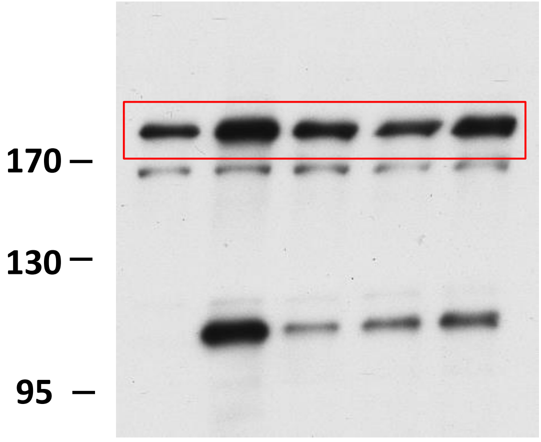

Vinculin

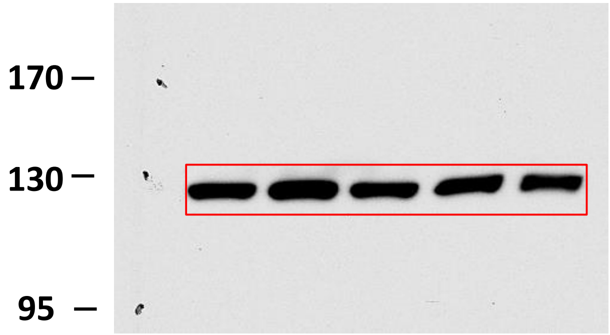

cyclinE

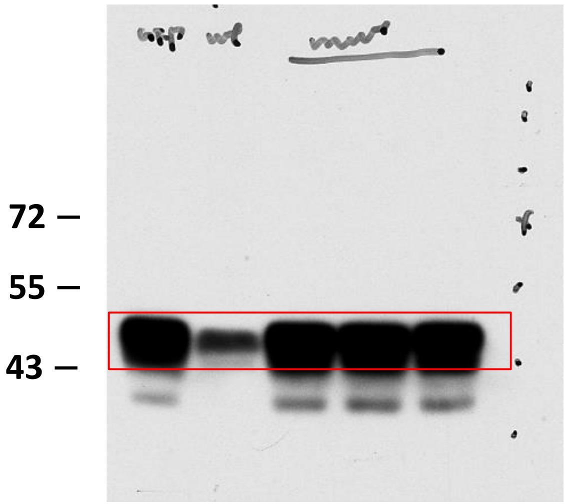

HA

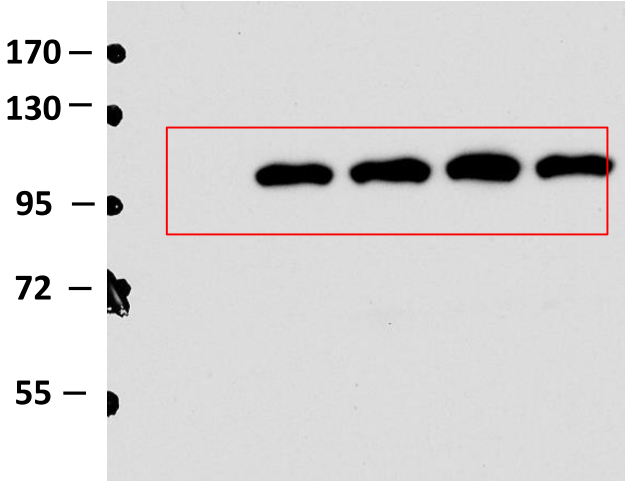

Uncropped blots related to Figure 2b, 2c

Fig2b, HA (IP)

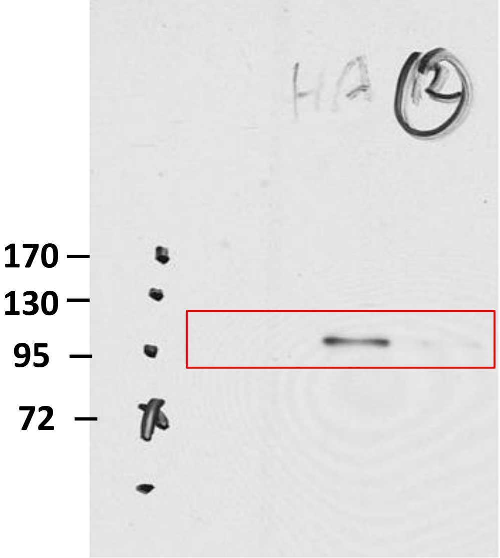

Fig2b, HA (lysates)

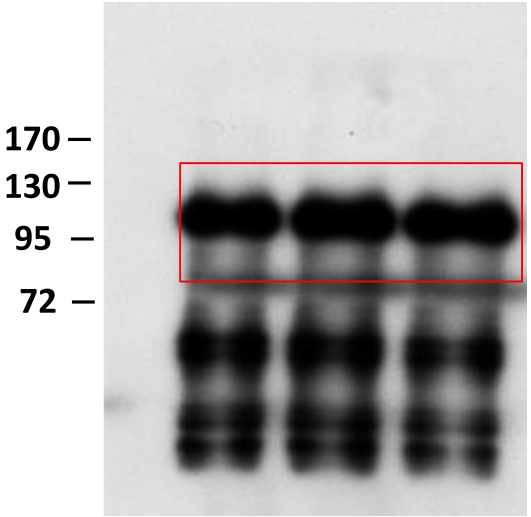

Fig2b, Flag (IP)

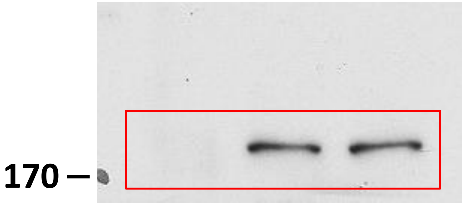

Fig2b, Flag (lysates)

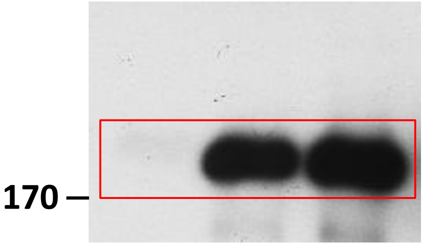

FIg2c, HA

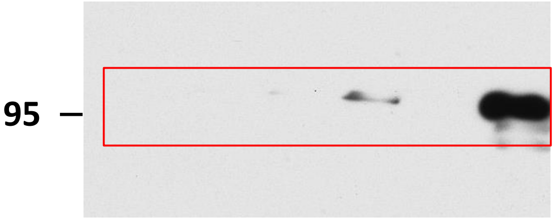

FIg2c, GST

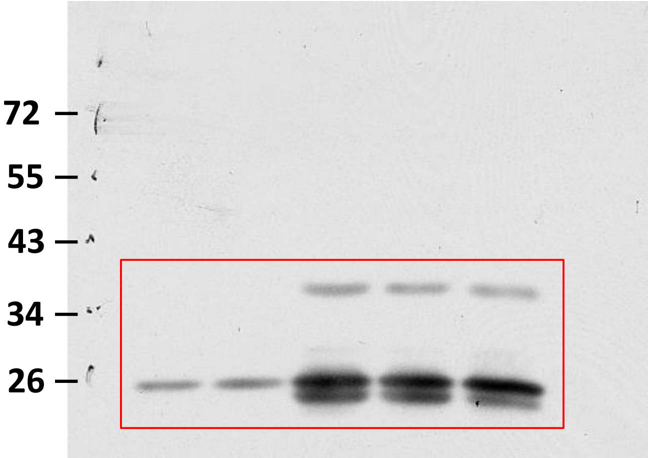

Uncropped blots related to Figure 2d,2e

Fig 2d-HA (IP)

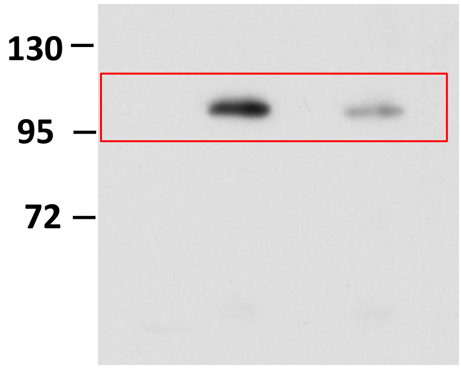

Fig 2d-Flag (IP)

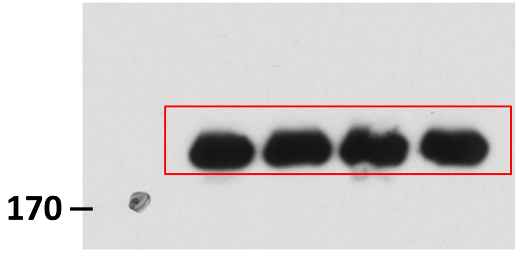

Fig 2d-Flag, HA (lysates)

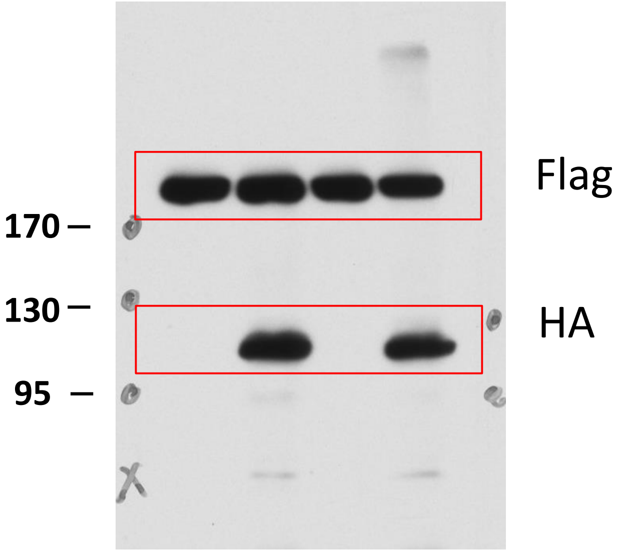

Fig 2e--Brg1

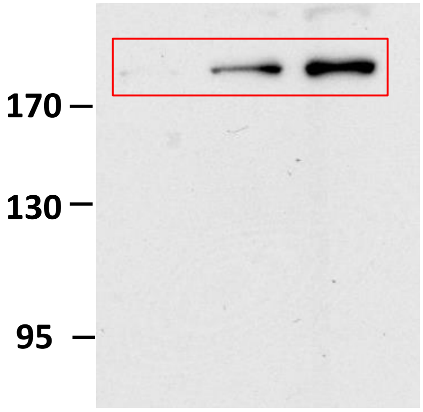

Fig 2e-CK1δ

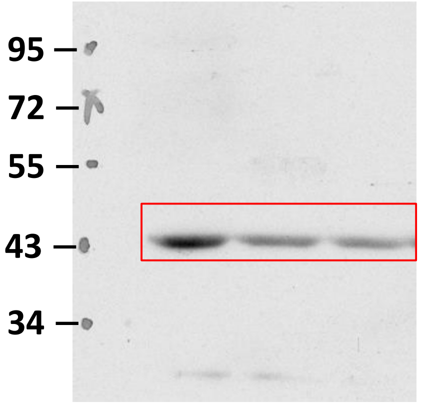

Fig 2e--Vinculin

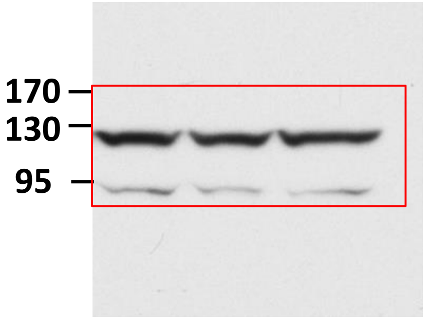

Uncropped blots related to Figure 2f

Flag-Brg1

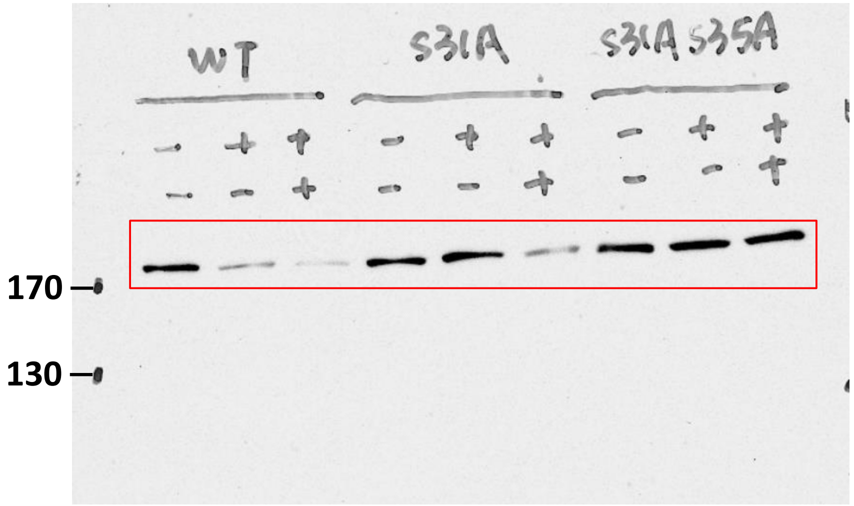

HA-FBW7

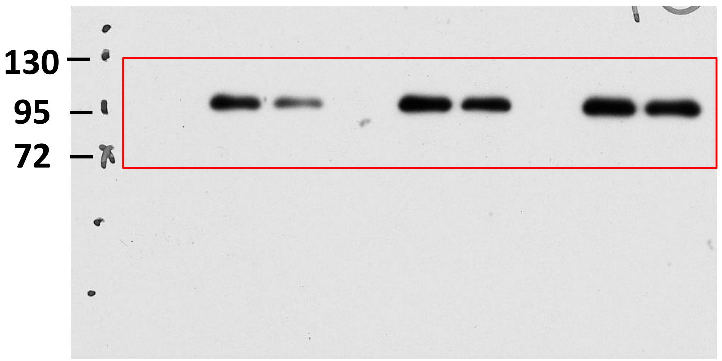

Myc-CK1δ

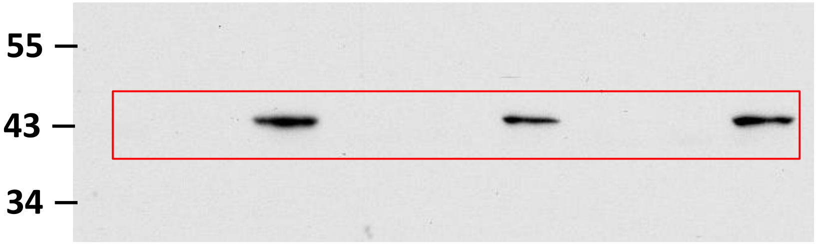

GFP

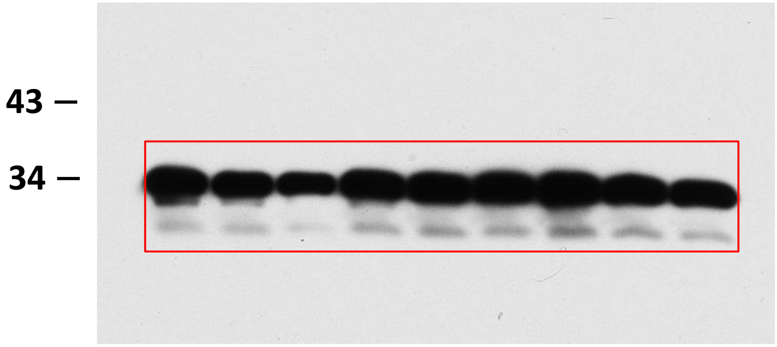

Vinculin

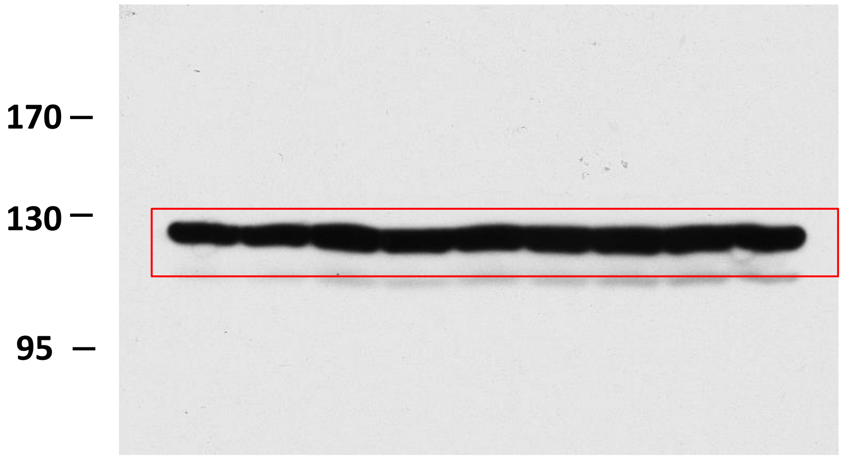

Uncropped blots related to Figure 2g

Flag-Brg1

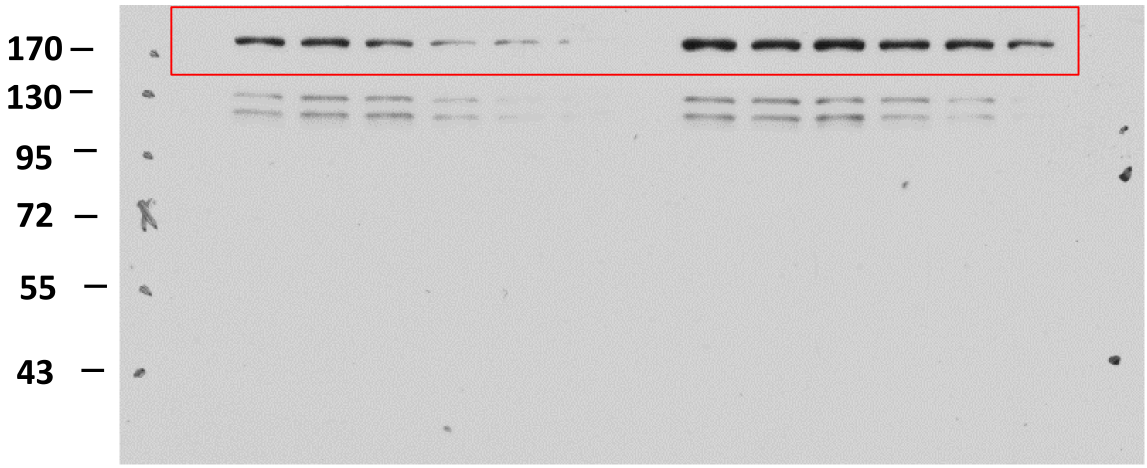

HA-FBW7

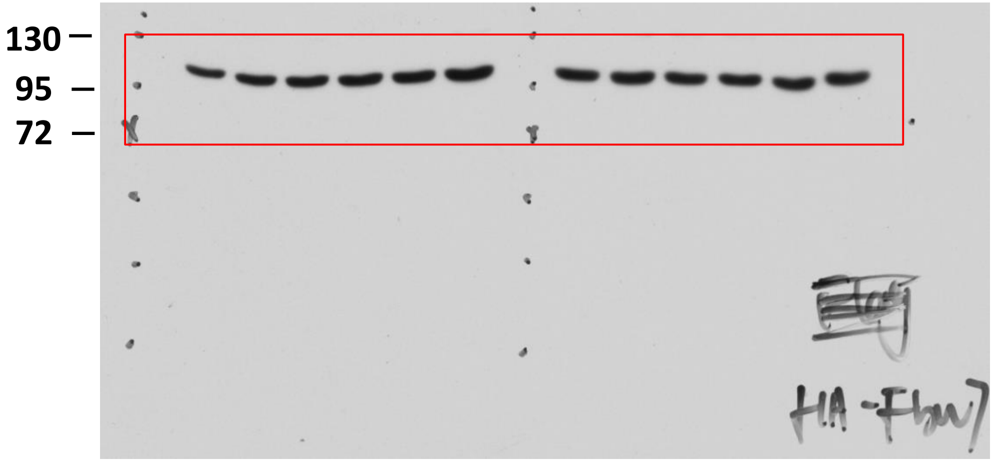

Vinculin

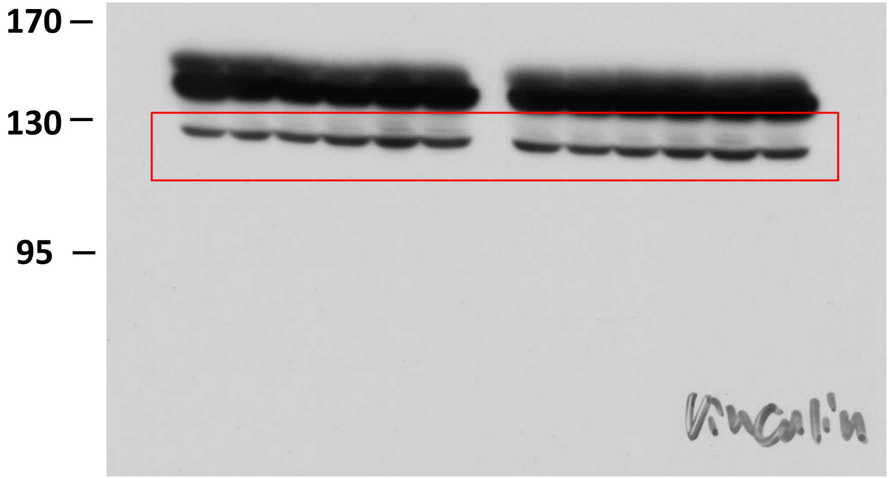

GFP

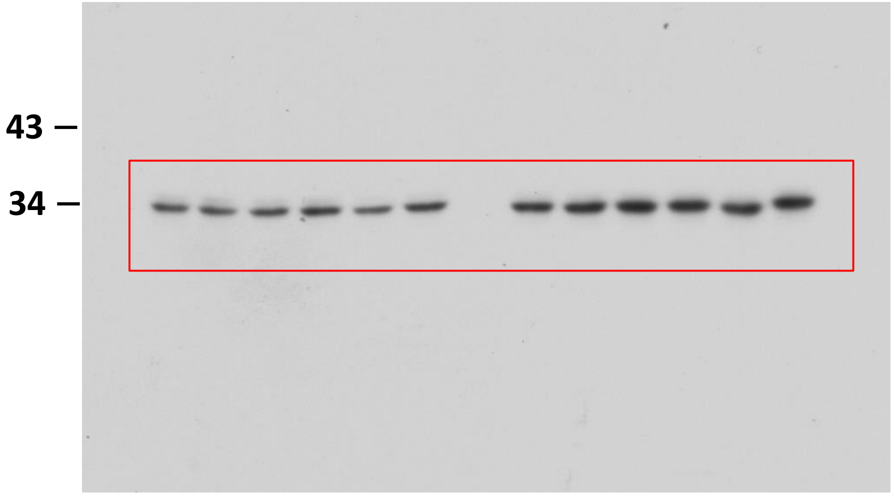

Uncropped blots related to Figure 2i

Flag-Brg1

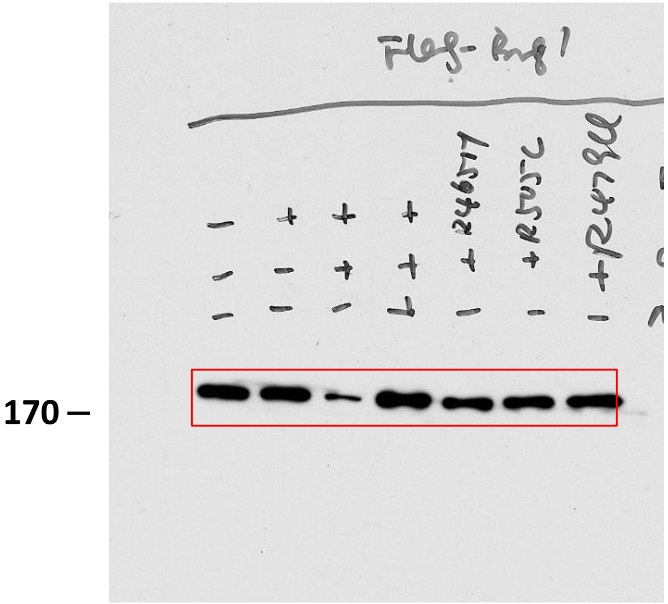

HA-FBW7, GFP

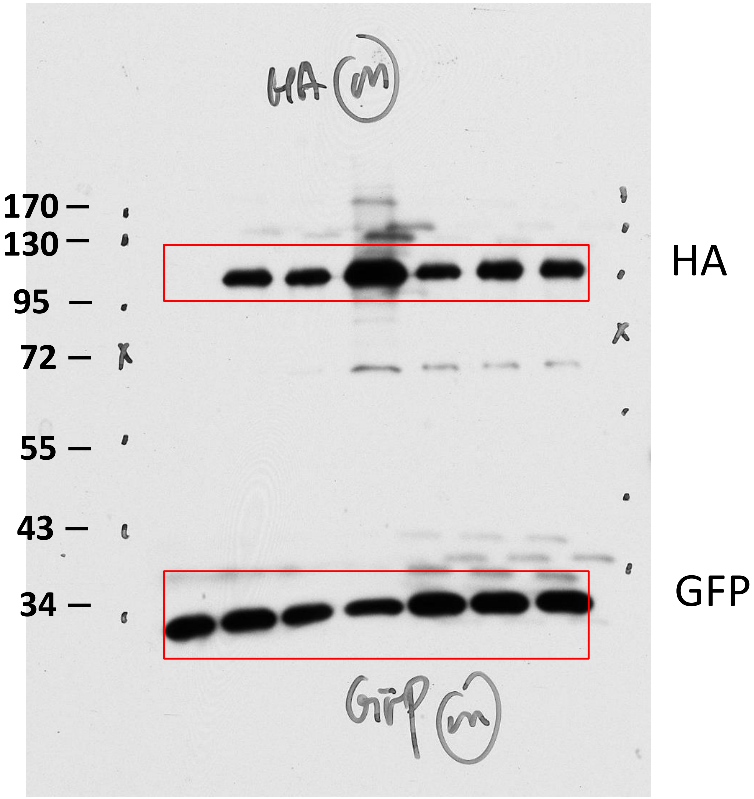

Myc-CK1δ

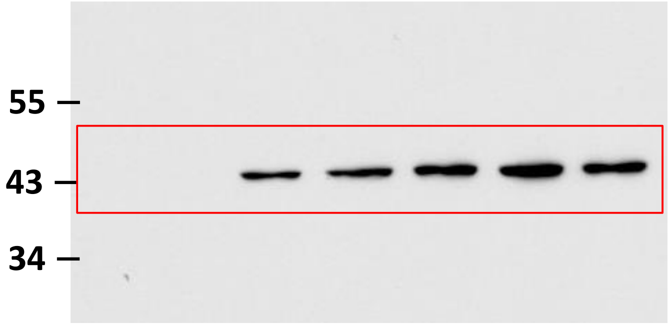

Vinculin

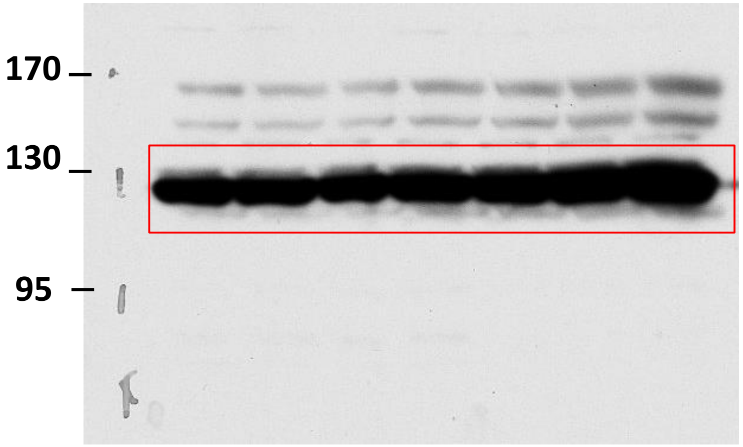

Uncropped blots related to Figure 2j

Flag (Ni-NTA)

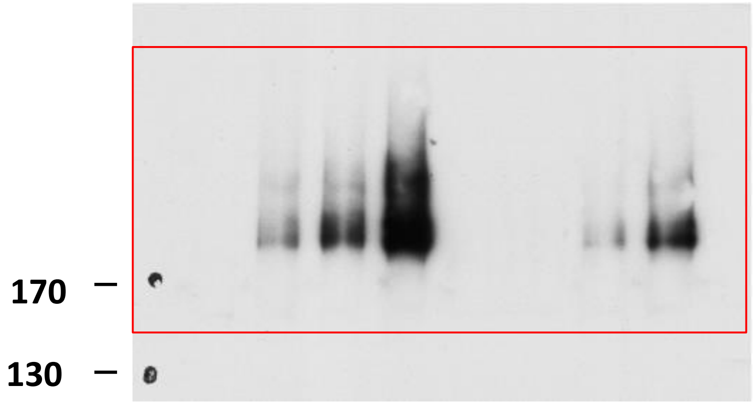

Flag (lysates)

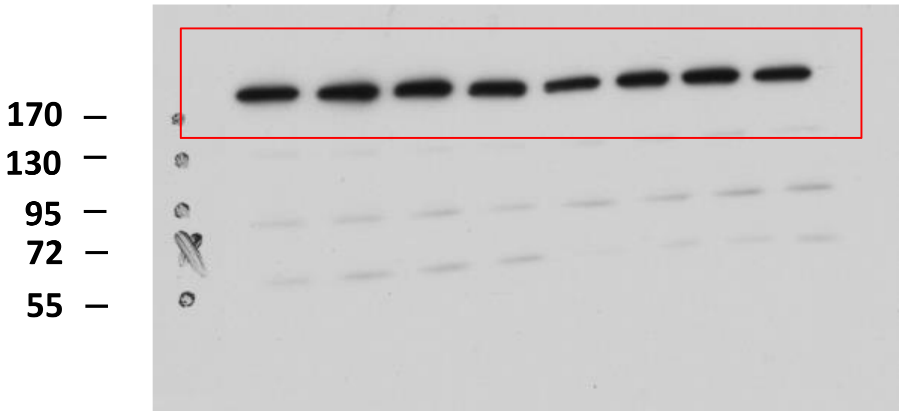

HA (lysates)

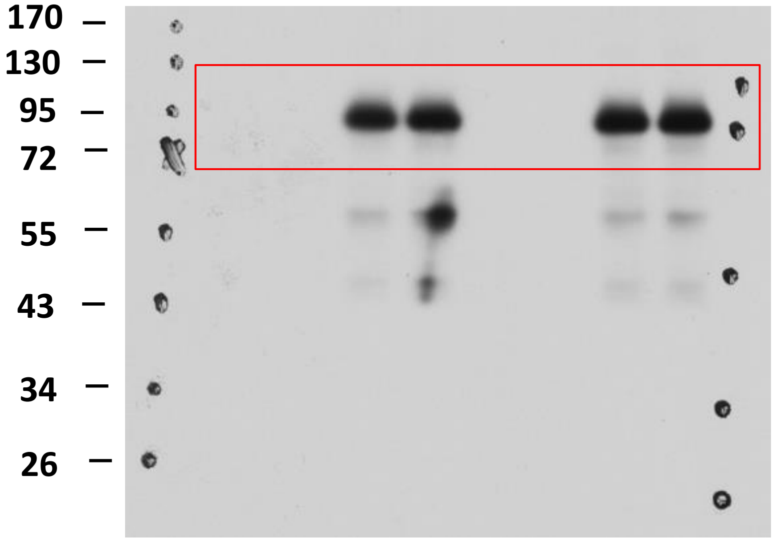

Myc (lysates)

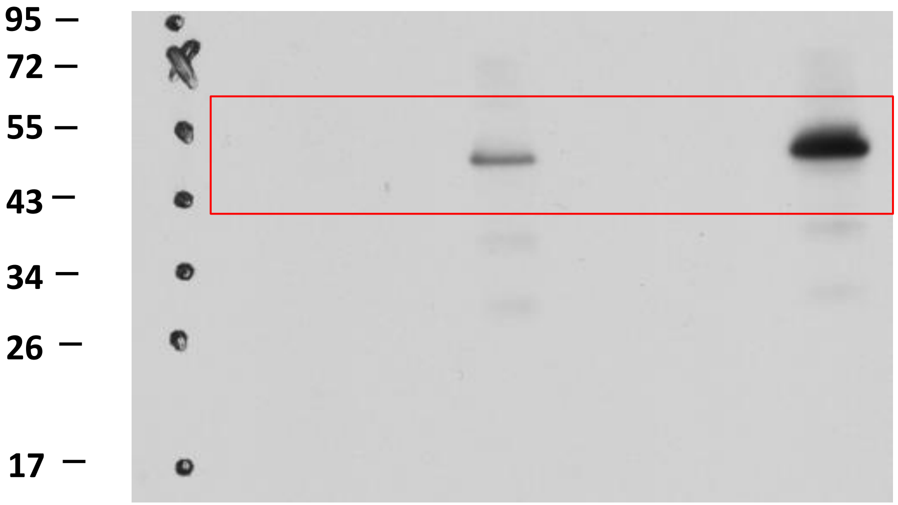

Uncropped blots related to Figure 4a, 4h

Fig 4a-Brg1

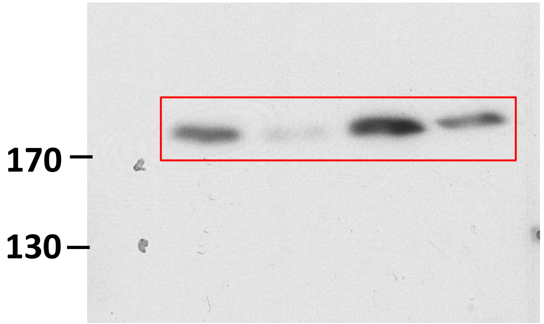

Fig4a-FBW7

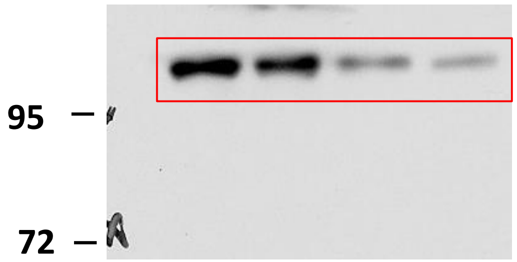

Fig4a-E-cadherin

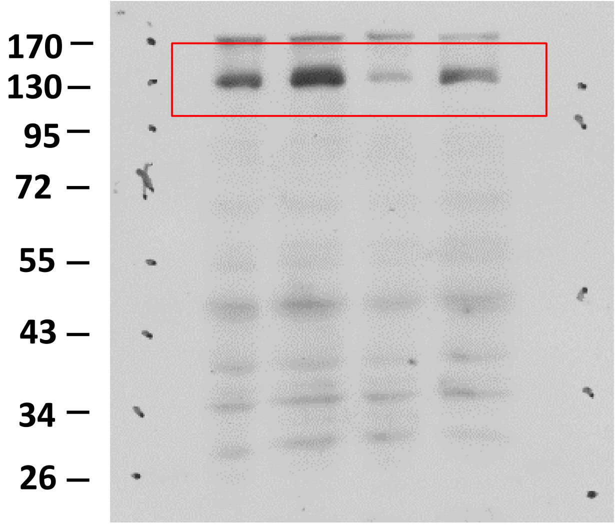

Fig4a-Vinculin

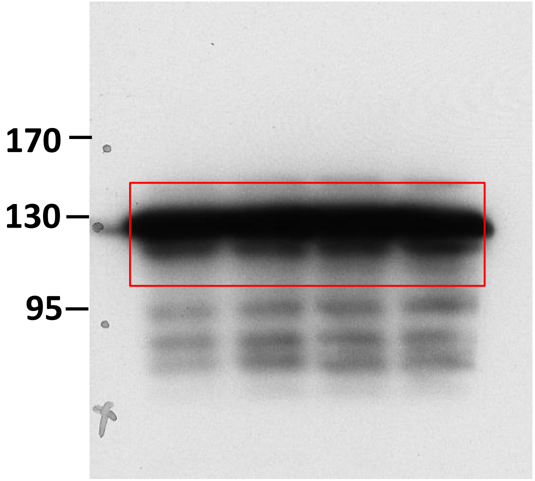

Fig4h-Brg1

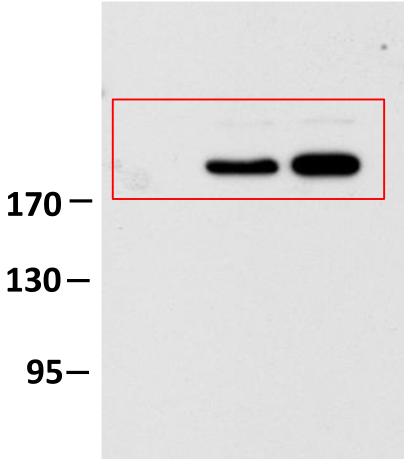

Fig4h-E-cadherin

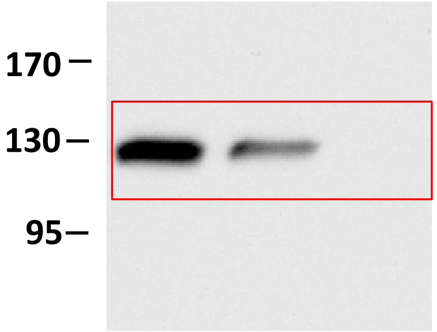

Fig4h-Vimentin

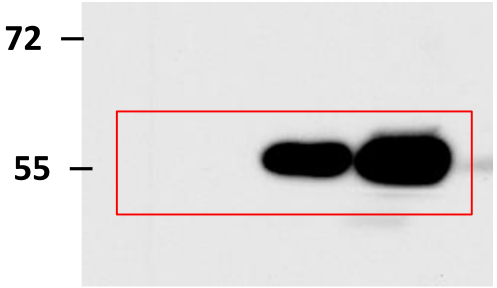

Fig4h-Vinculin

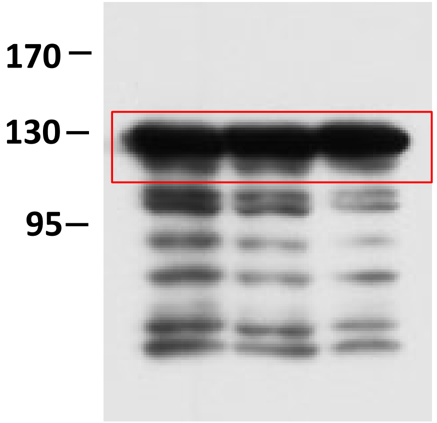

Uncropped blots related to Figure 5a

Brg1

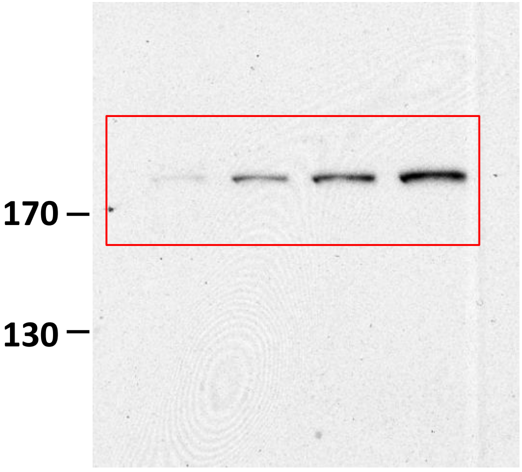

E-cadherin

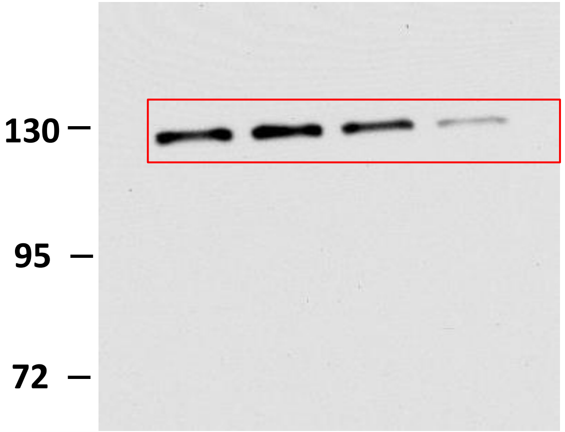

Vimentin

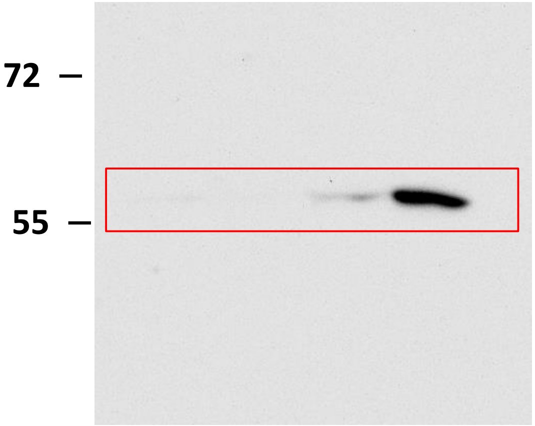

Snail

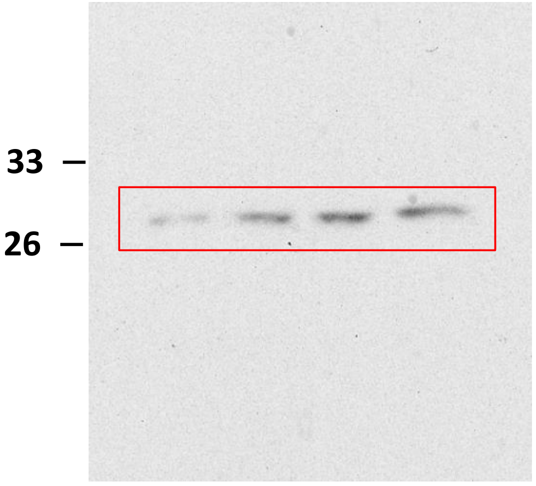

ZEB-1

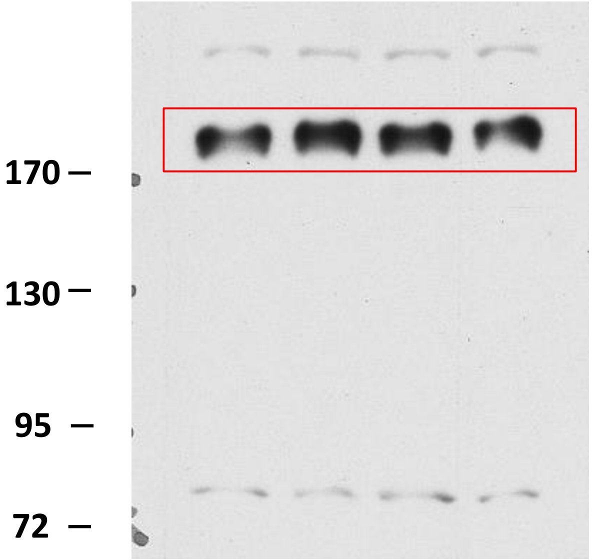

TWIST-1

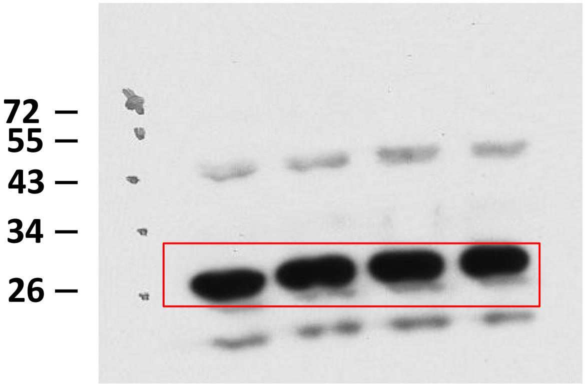

β-cateinin

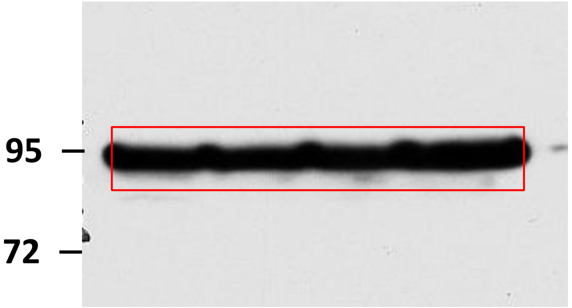

Vinculin

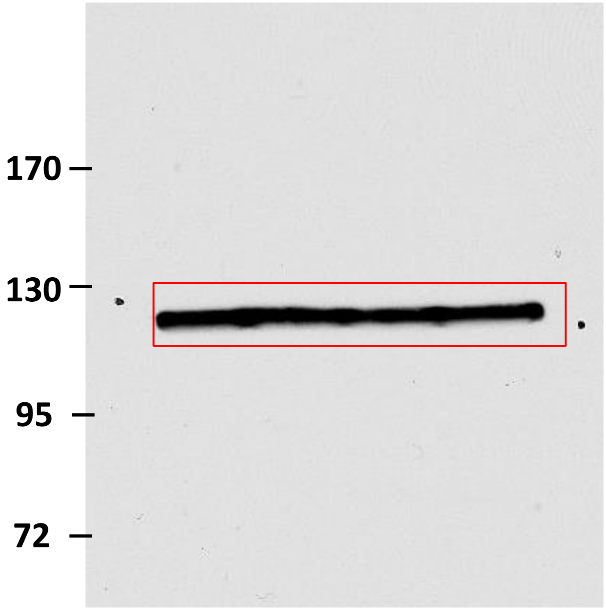

Uncropped blots related to Figure 5d

Brg1

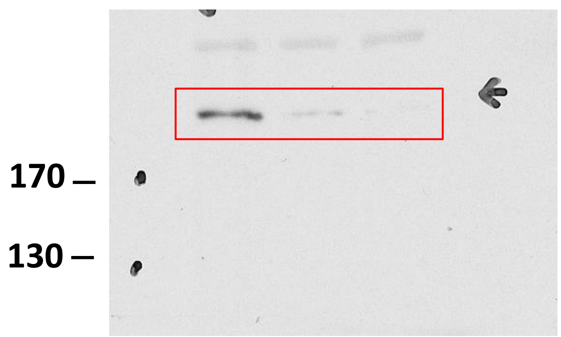

E-cadherin

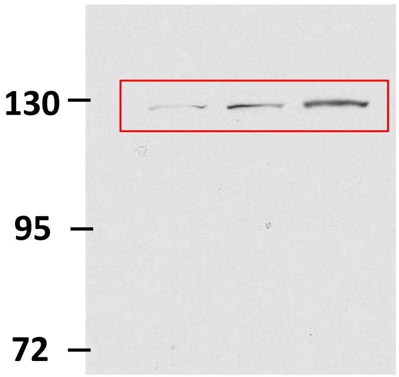

Vimentin

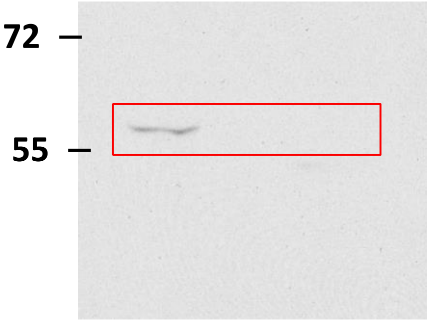

Snail

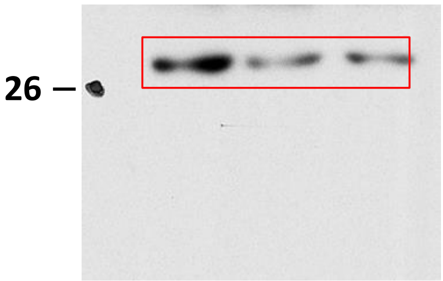

ZEB-1

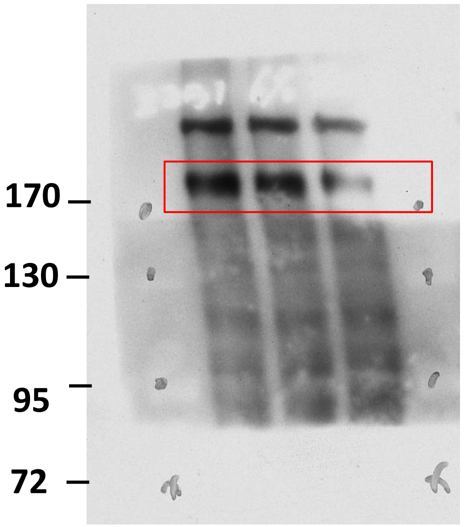

TWIST-1

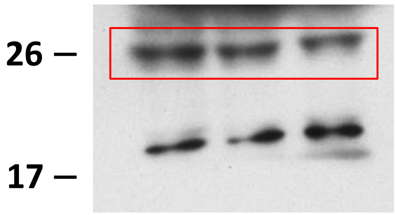

β-cateinin

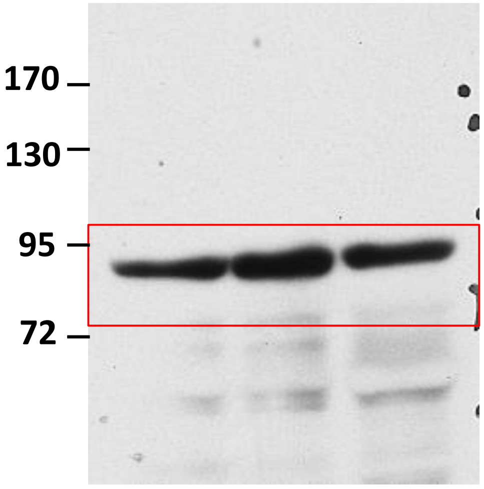

Vinculin

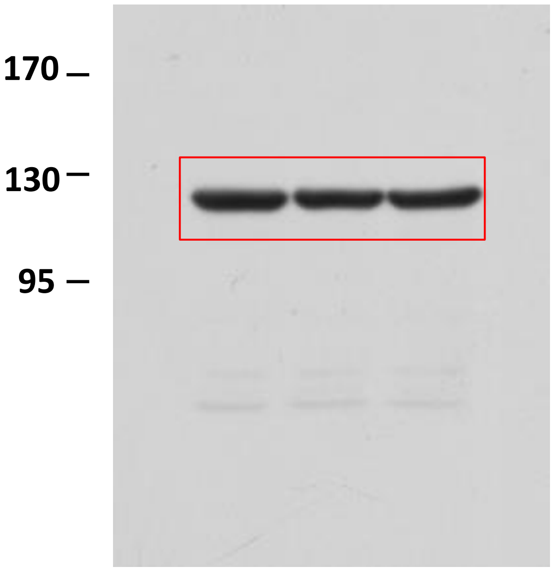

Uncropped blots related to Figure S1a

Brg1

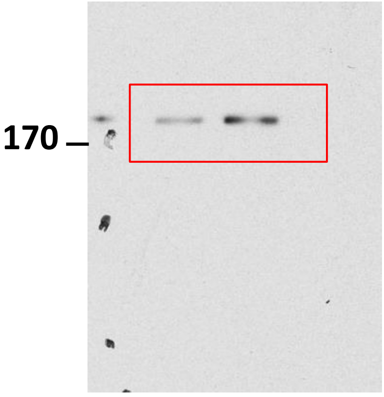

Arid1a

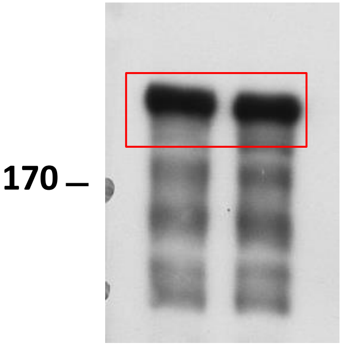

c-Myc

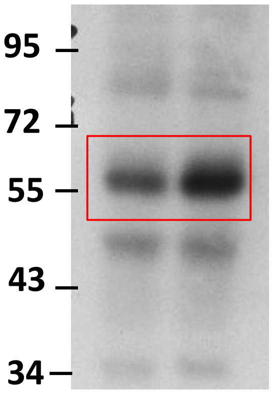

BRM

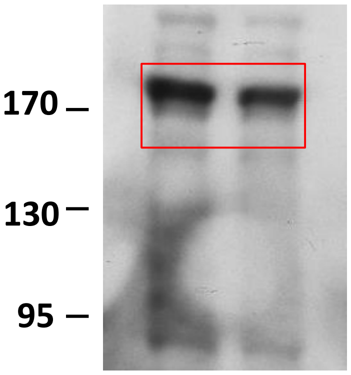

cyclinE

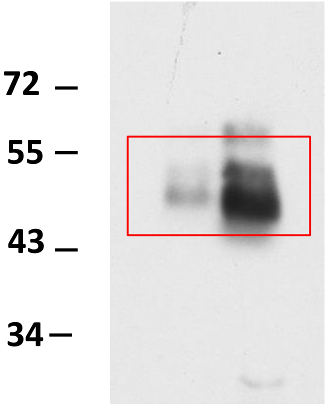

Vinculin

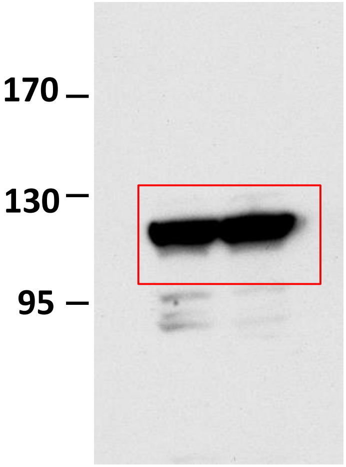

Uncropped blots related to Figure S1c

Brg1

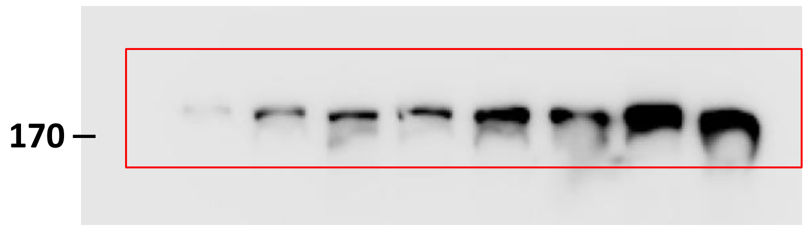

FBW7

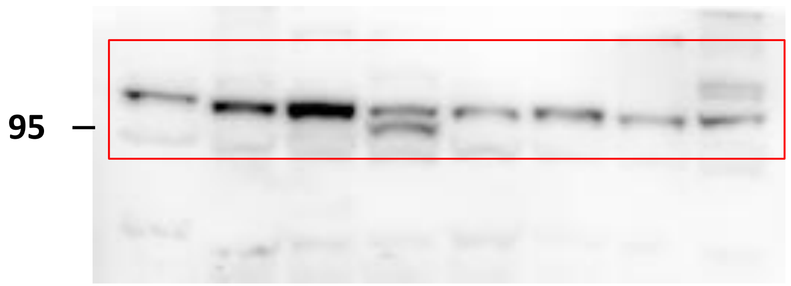

Actin

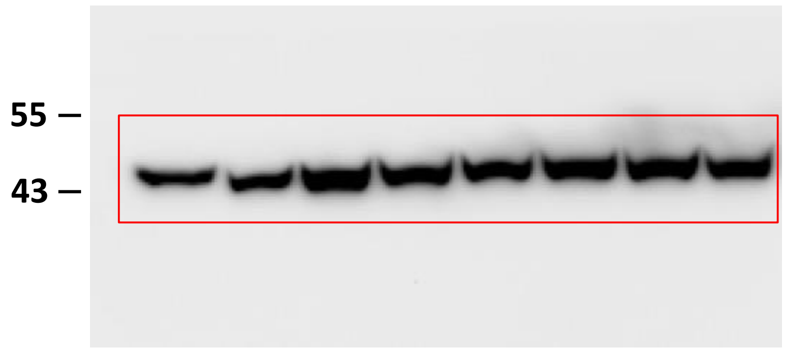

Uncropped blots related to Figure S1d

Brg1

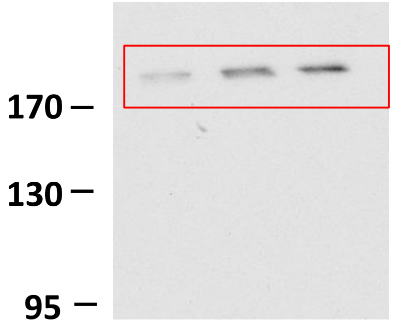

Arid1a

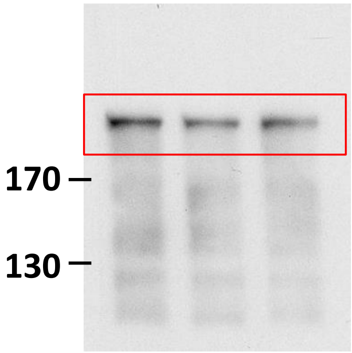

BRM

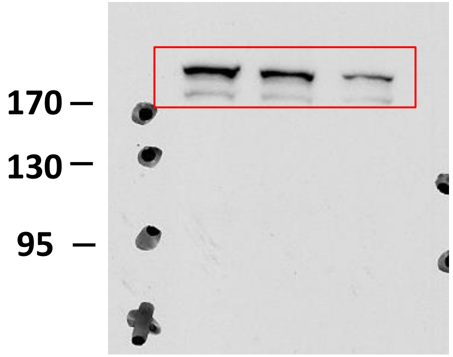

c-Myc

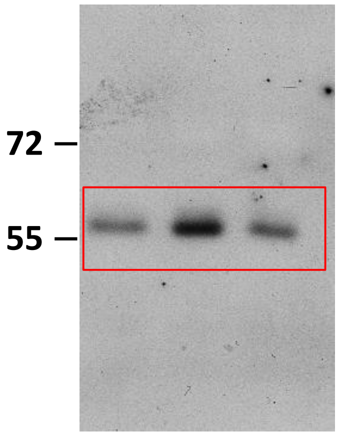

cyclinE

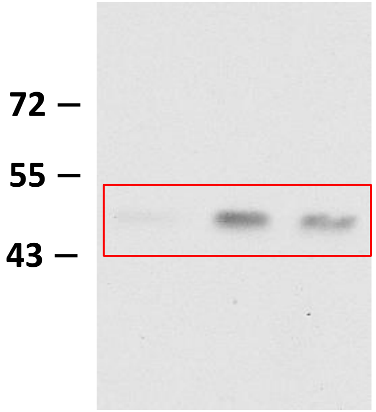

Vinculin

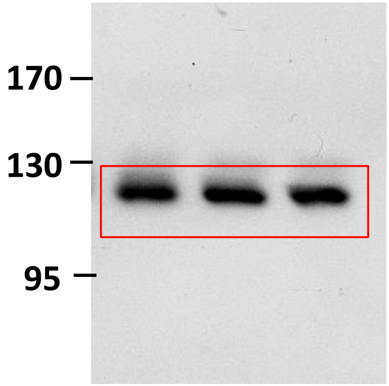

FBW7

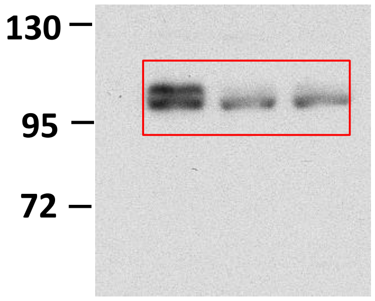

Uncropped blots related to Figure S1f, S1g

FigS1f-Brg1

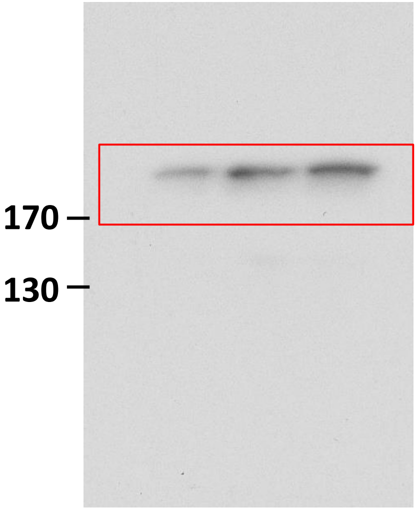

FigS1f-Cul1

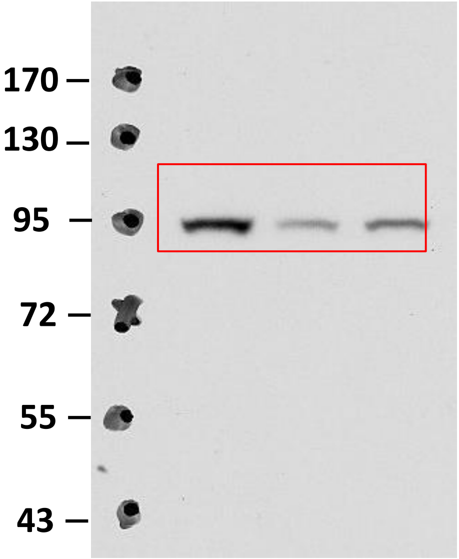

FigS1f-Vinculin

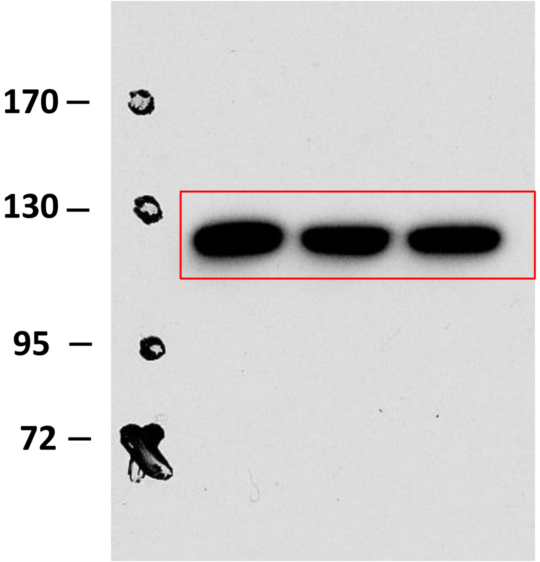

FigS1g-Brg1 (IP)

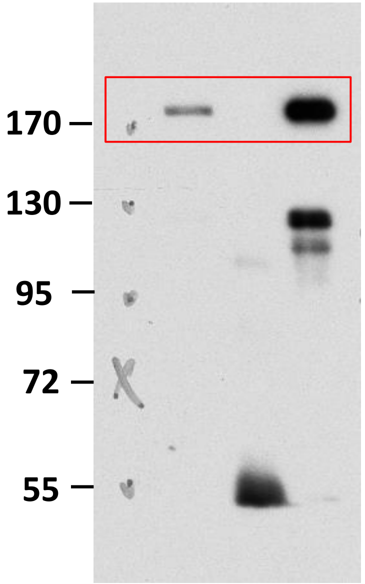

FigS1g-FBW7 (IP)

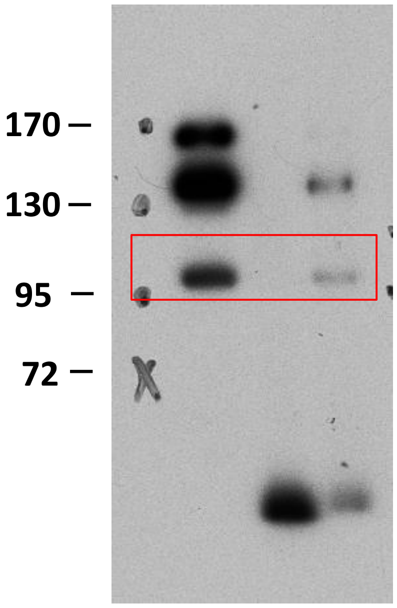

Uncropped blots related to Figure S2b

Flag (IP)

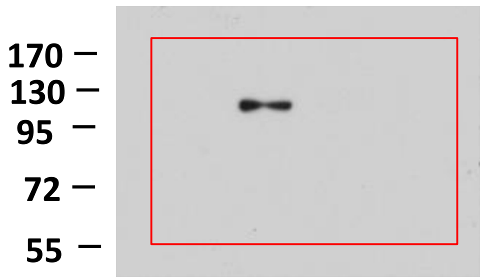

Flag (lysates)

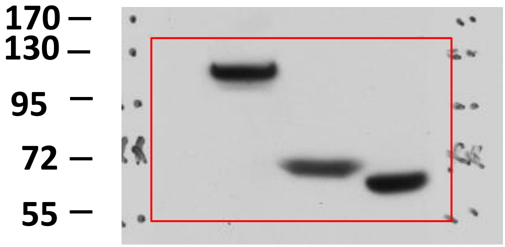

HA (IP)

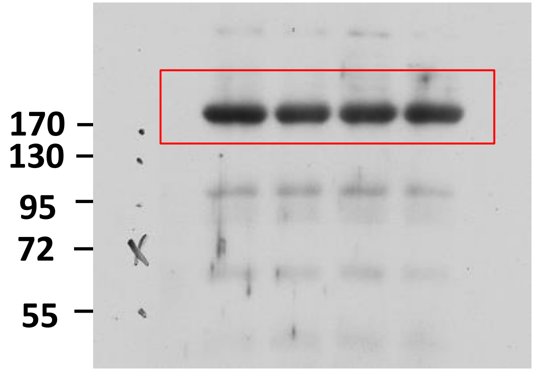

Vinculin (lysates)

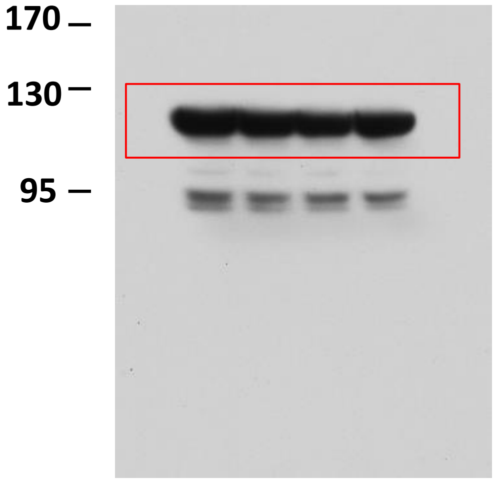

HA (lysates)

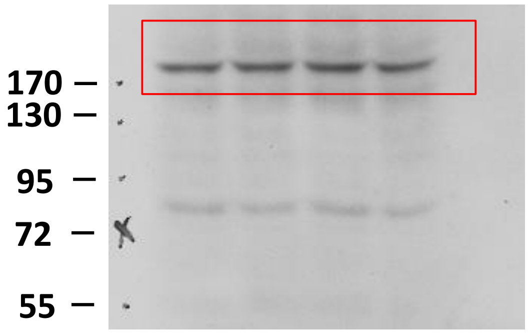

Uncropped blots related to Figure S2c

Brg1

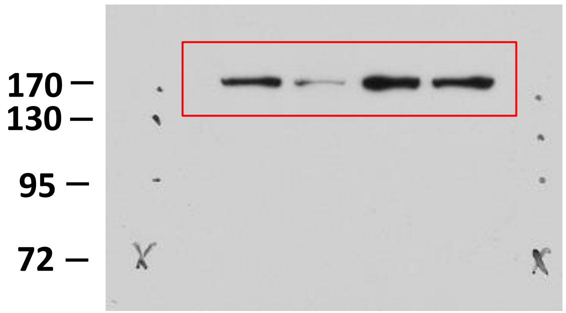

Cyclin E

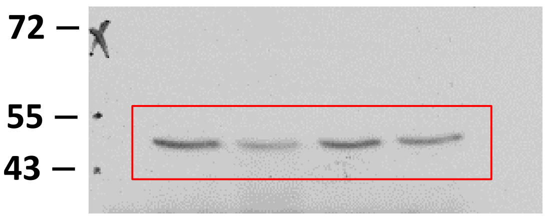

c-myc

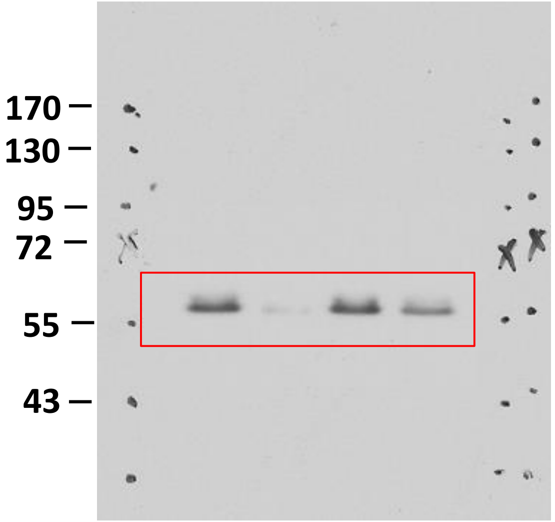

Vinculin

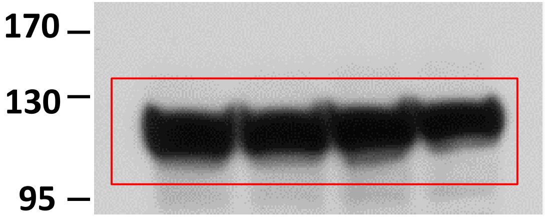

Flag

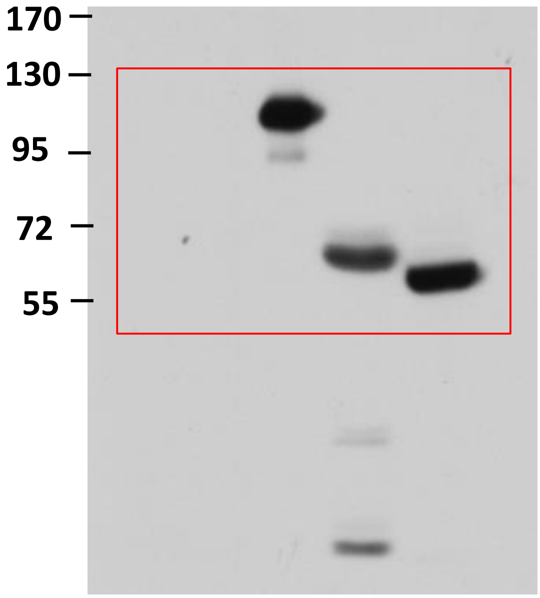

Uncropped blots related to Figure S3a

Flag

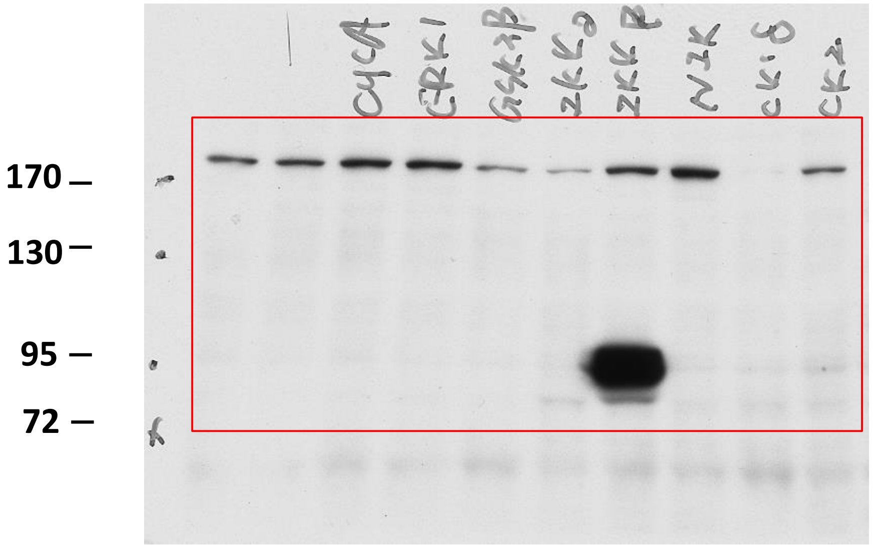

HA

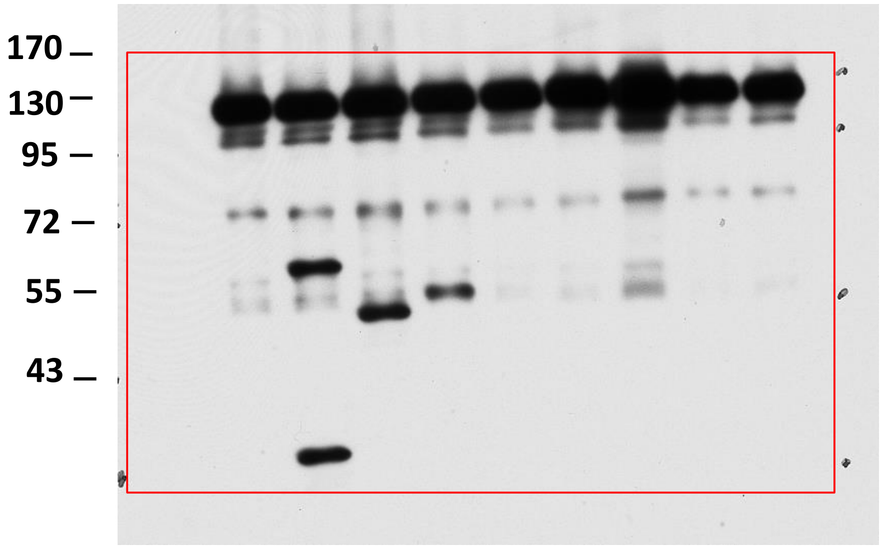

Myc

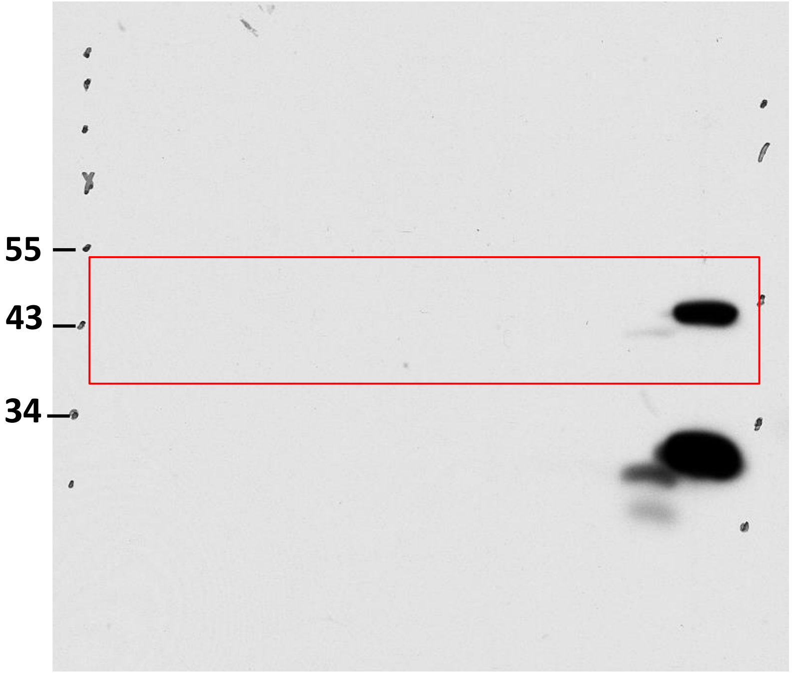

GFP

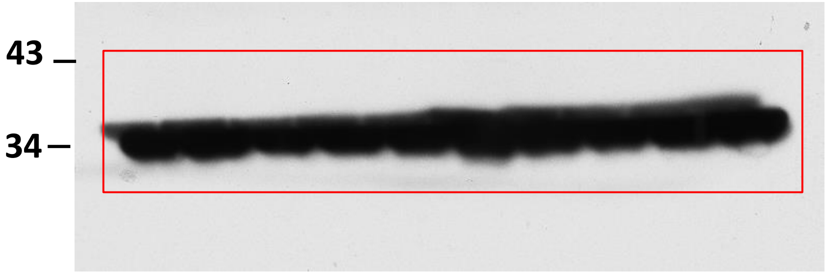

Vinculin

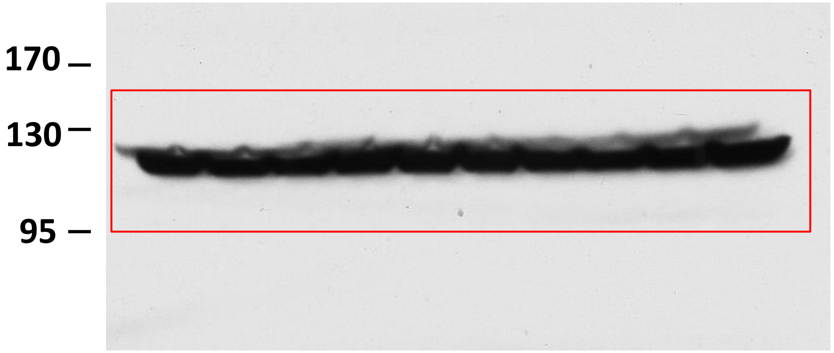

Uncropped blots related to Figure S3b

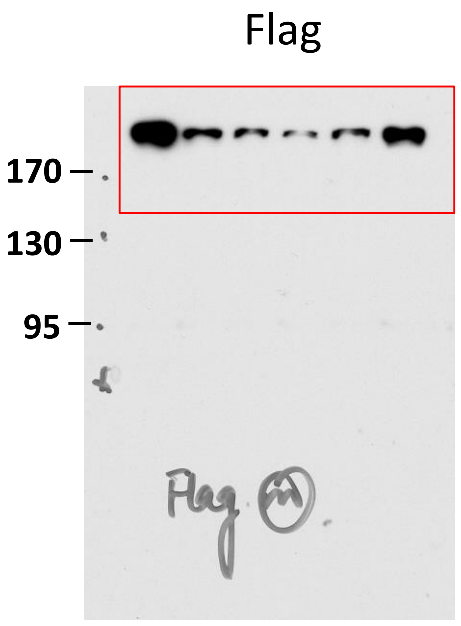

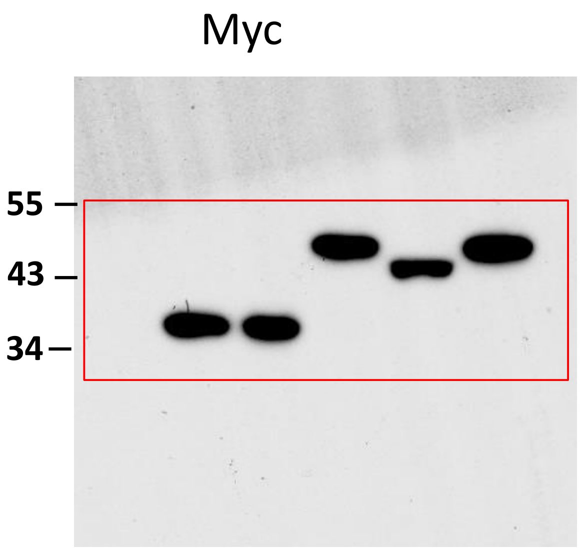

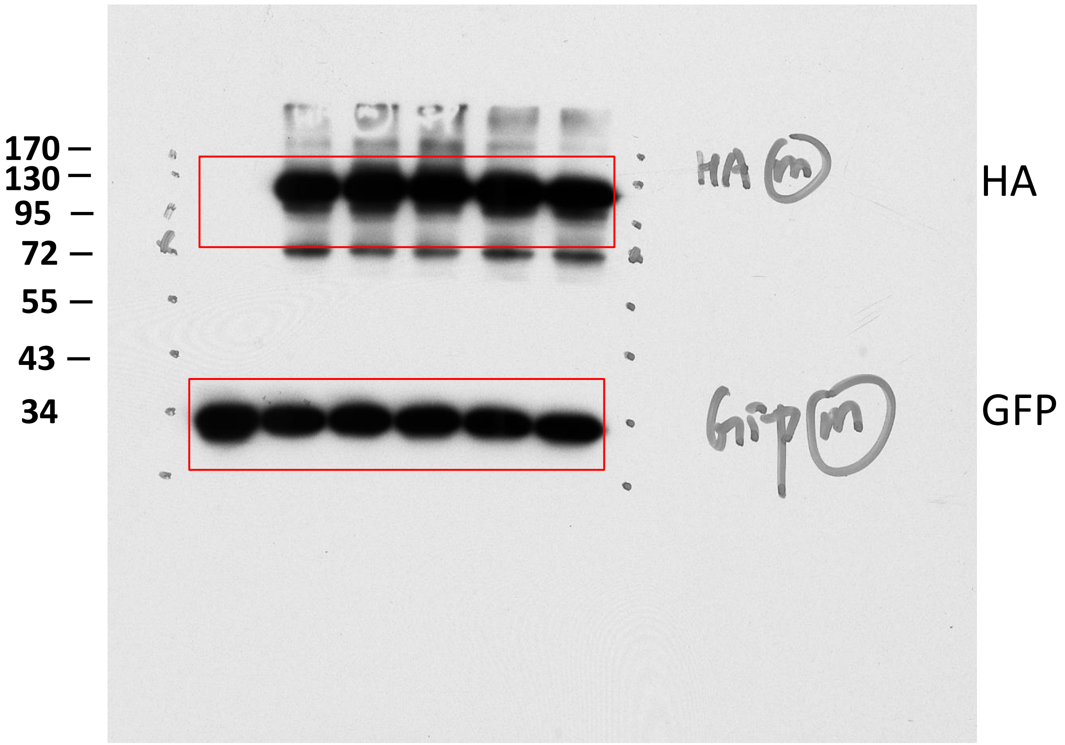

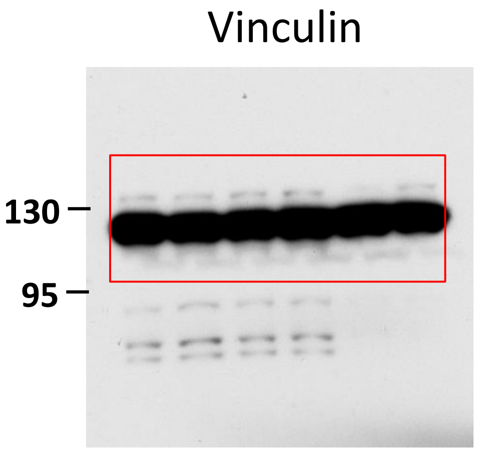

Uncropped blots related to Figure S3c, S3d

Fig S3C-Myc (IP)

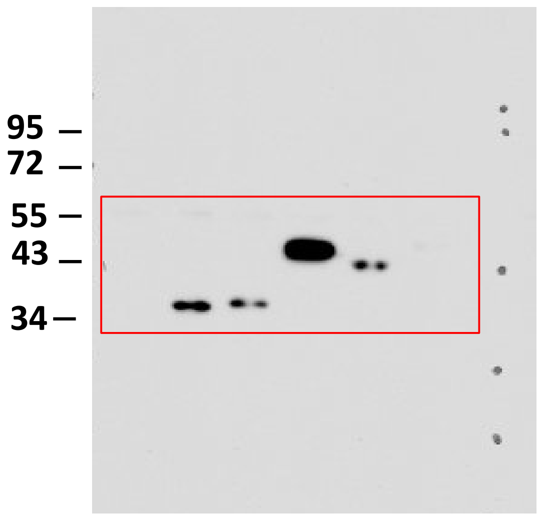

Fig S3C-Flag (IP)

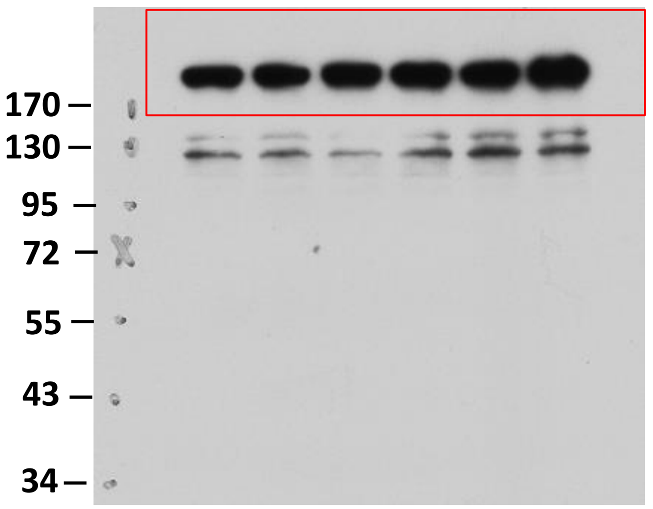

Fig S3c-Myc (lysates)

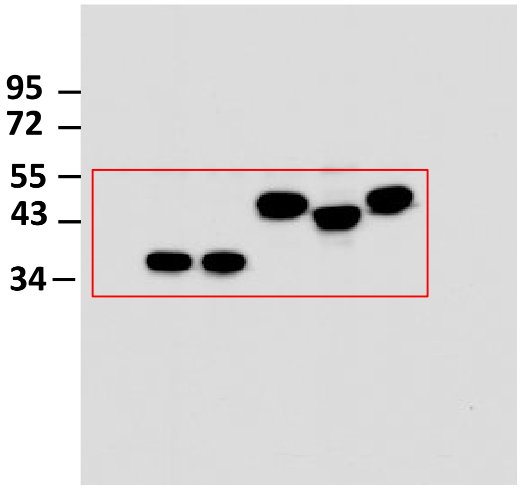

Fig S3C-Flag (lysates)

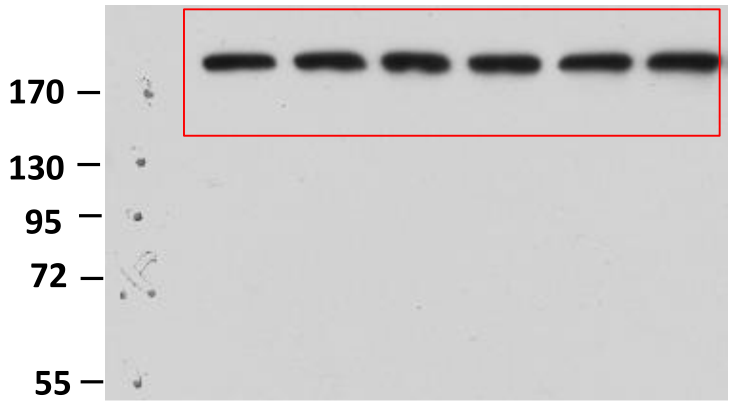

Fig S3d-Brg1

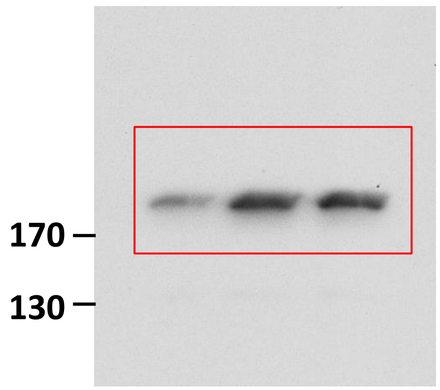

Fig S3d-CK1δ

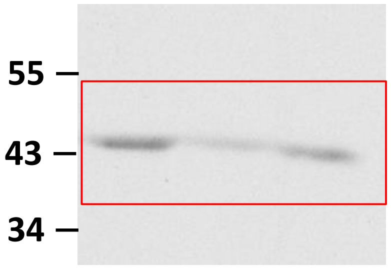

Fig S3d-Vinculin

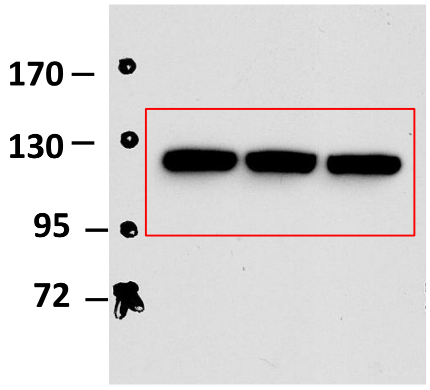

Uncropped blots related to Figure S3e

Flag (Ni-NTA)

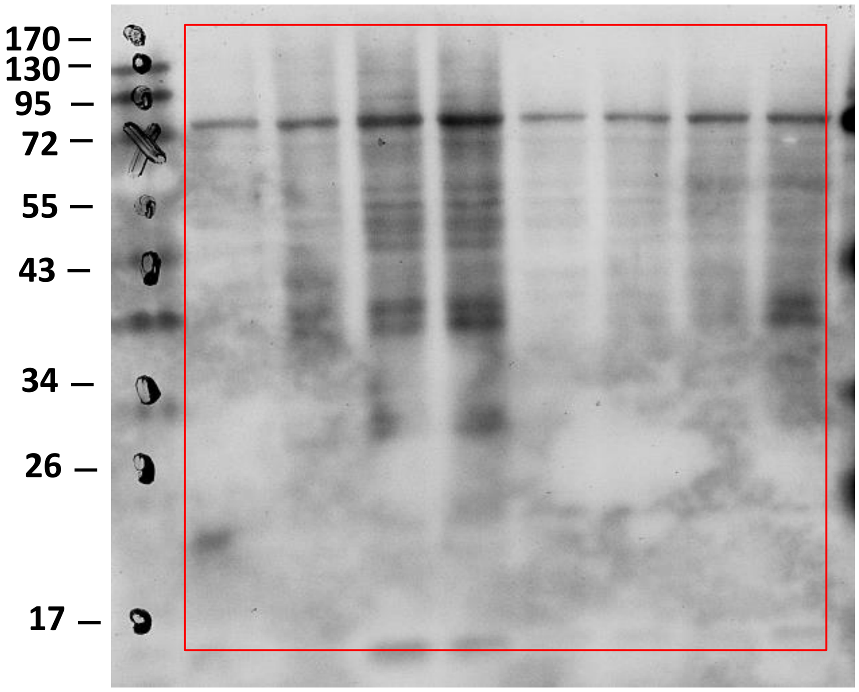

Flag (lysates)

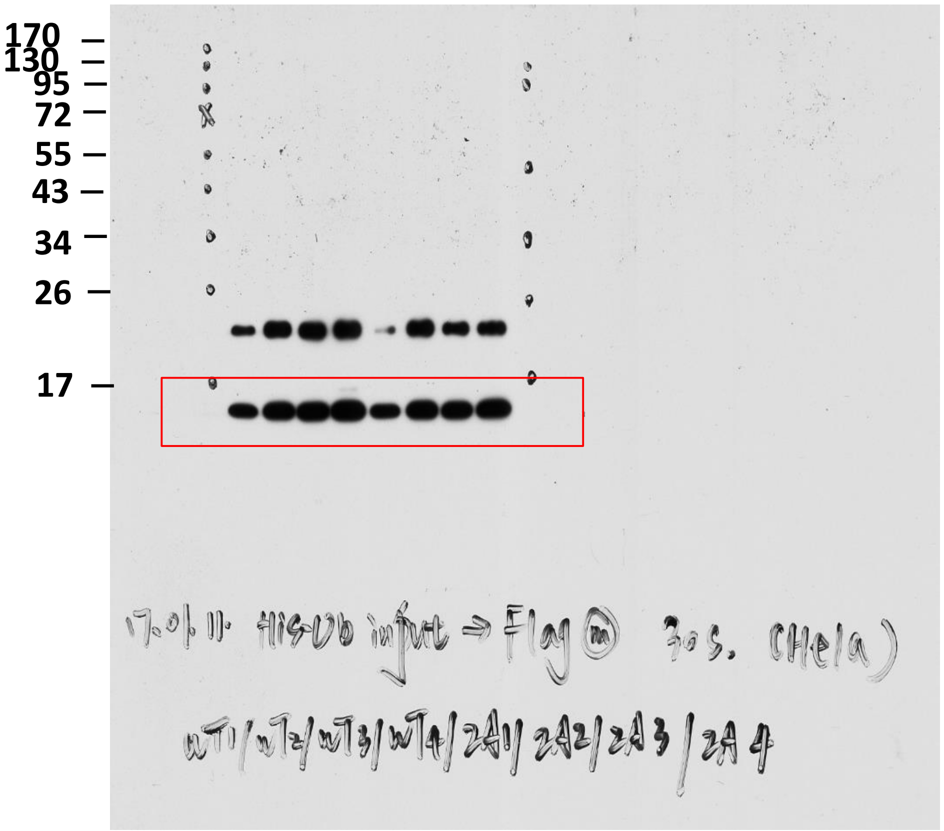

HA

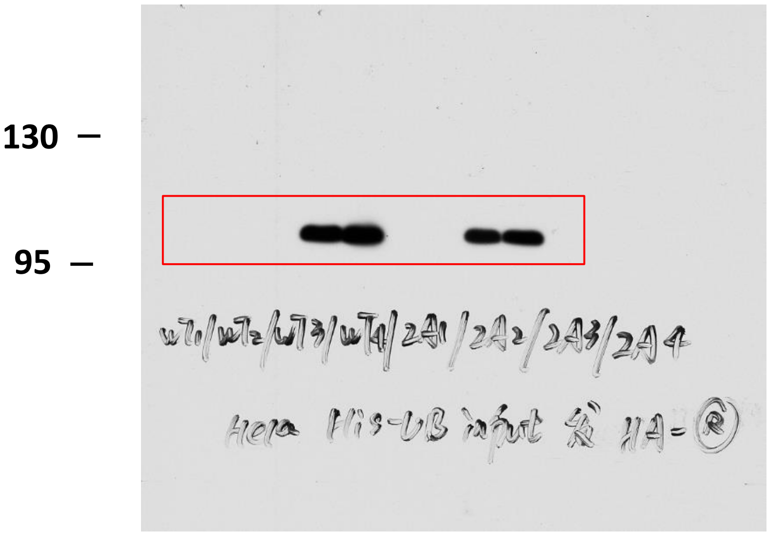

Myc

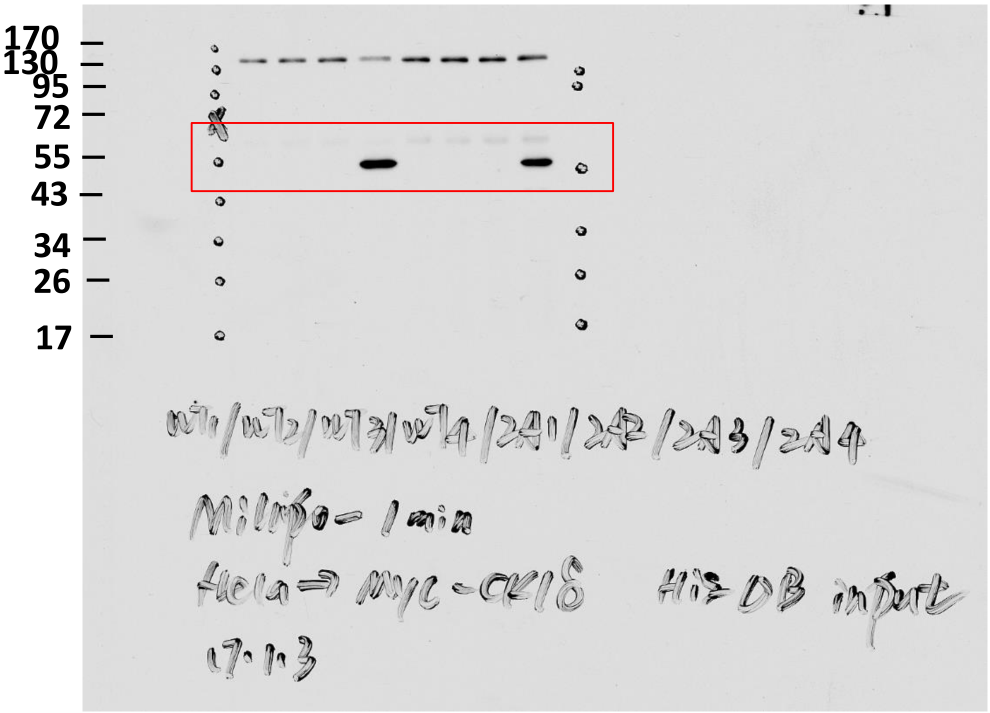

Uncropped blots related to Figure S4a

Brg-1

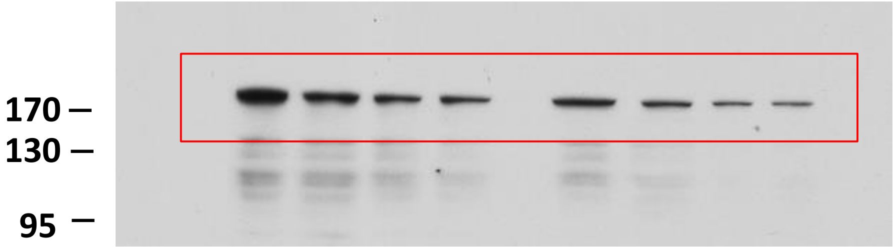

Fbw7

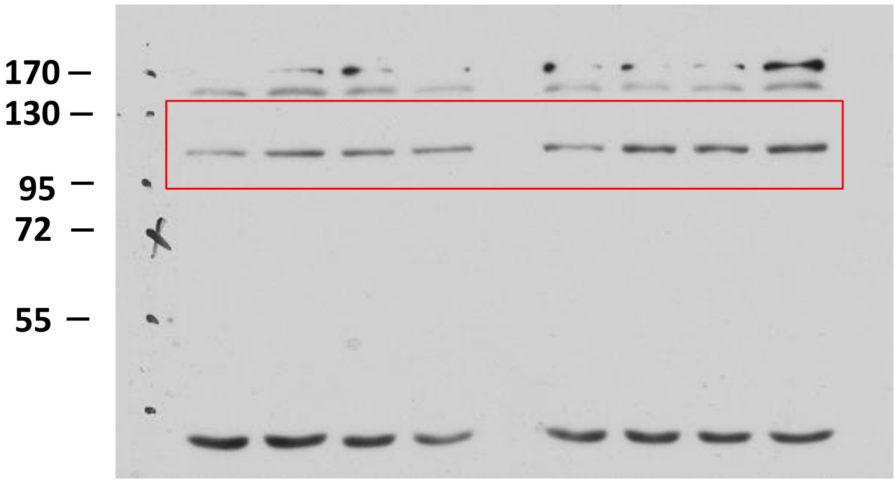

Vinculin

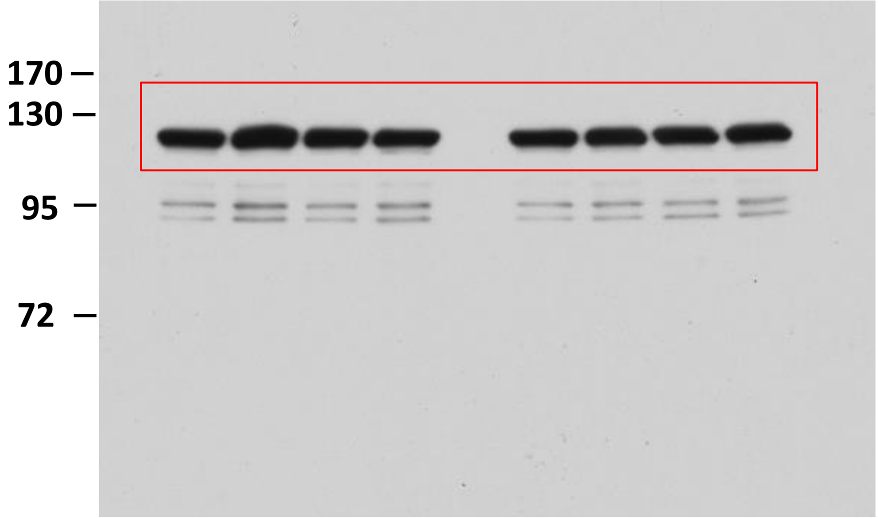

Uncropped blots related to Figure S4b

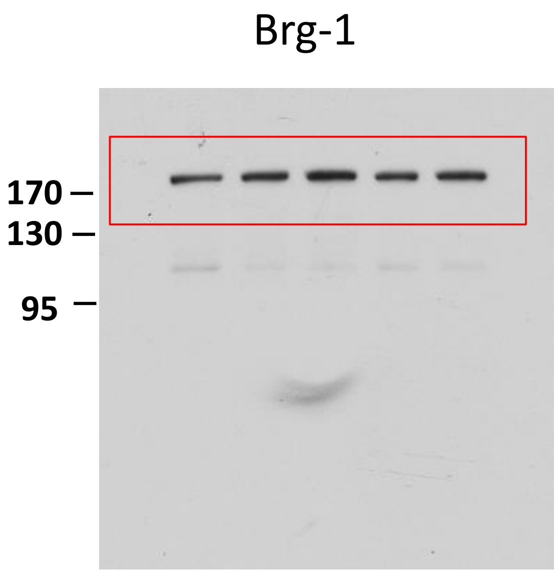

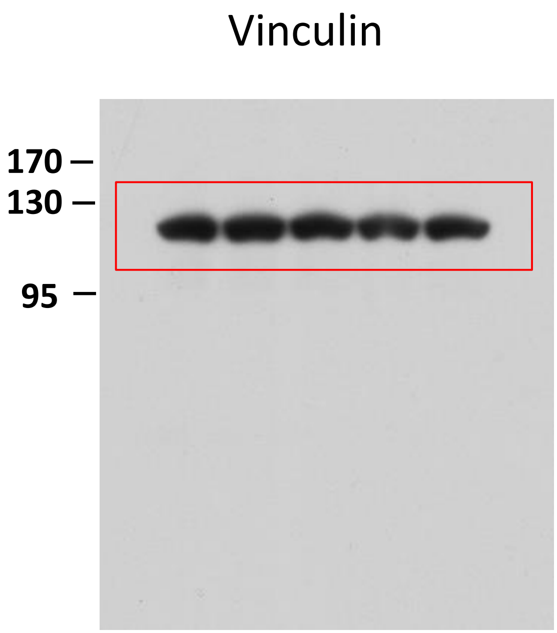

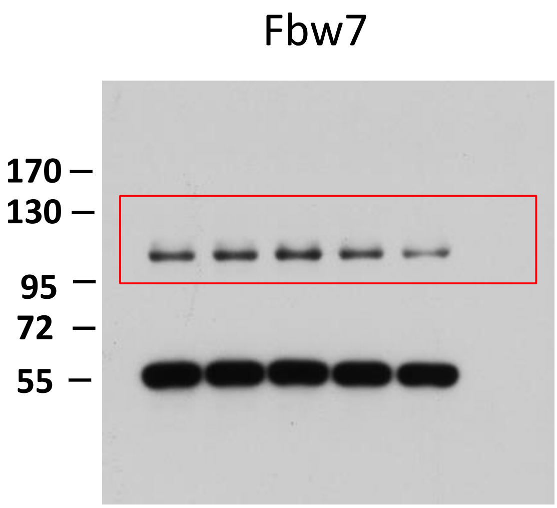

Uncropped blots related to Figure S4c

Brg-1

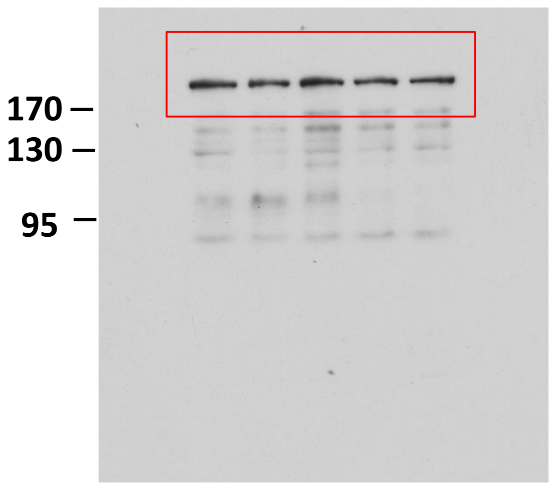

Cyclin B

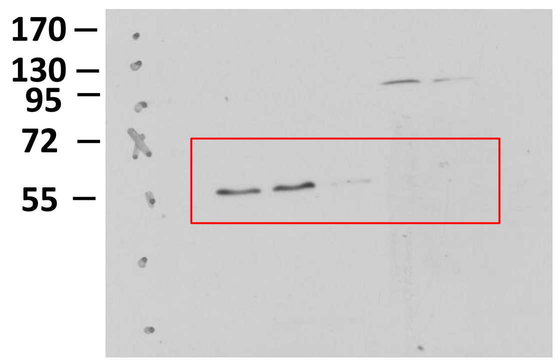

Fbw7

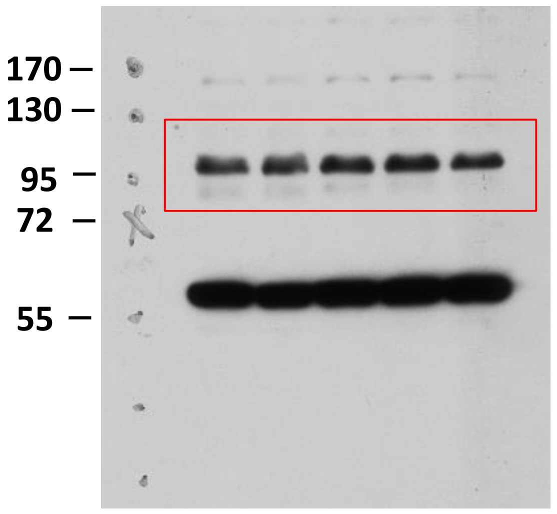

Cyclin E

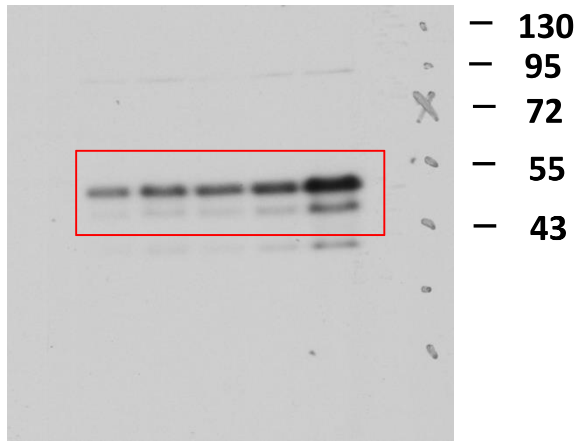

Vinculin

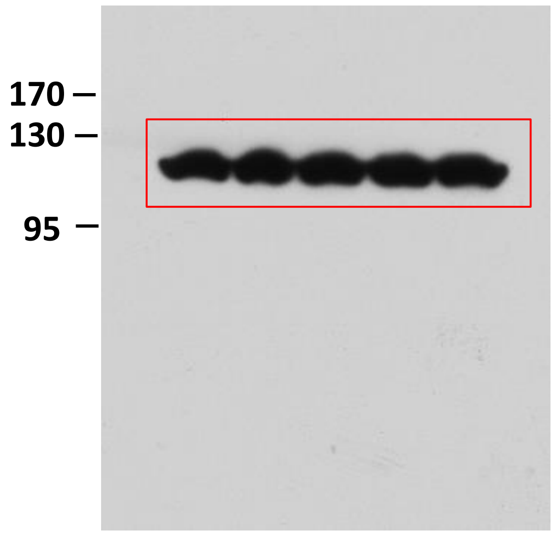

Uncropped blots related to Figure S4d

Brg-1

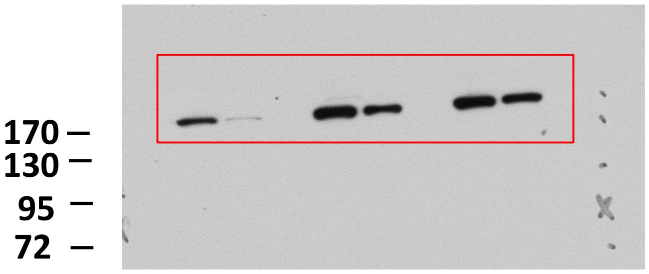

Vinculin

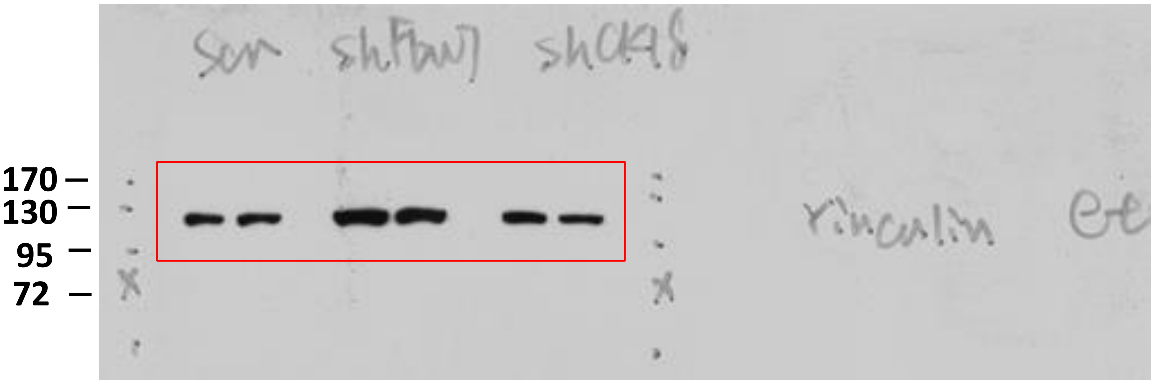

Fbw7

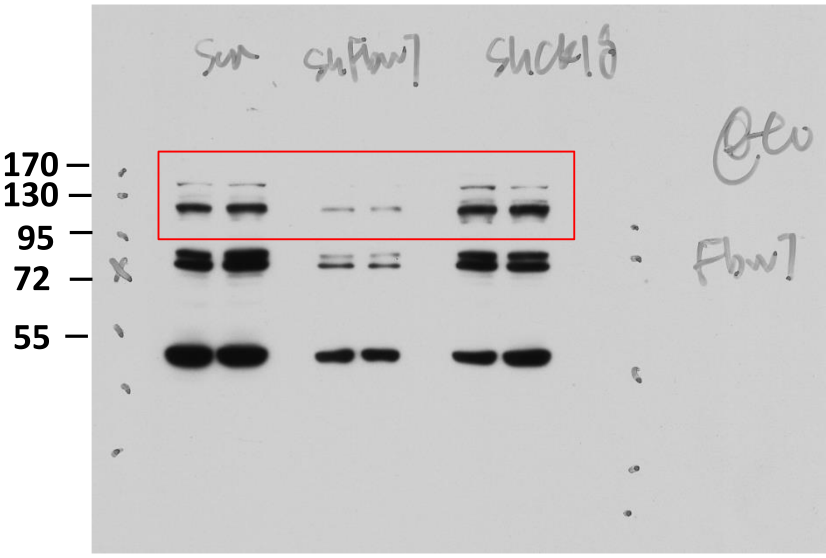

CK 1δ

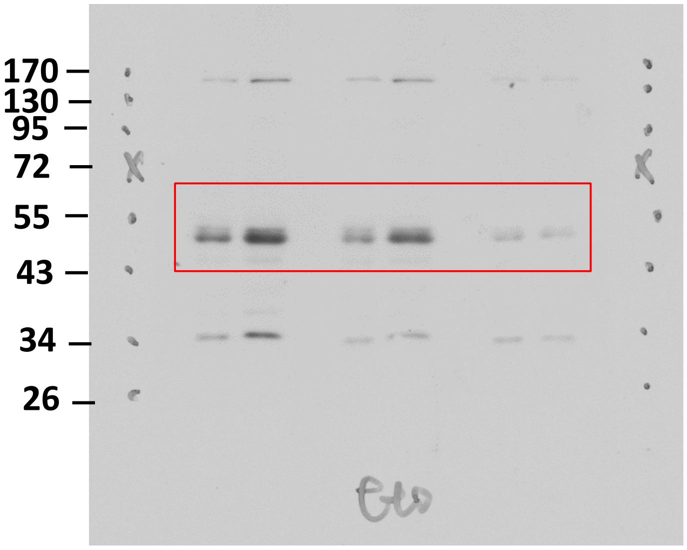

Uncropped blots related to Figure S4e

Brg-1

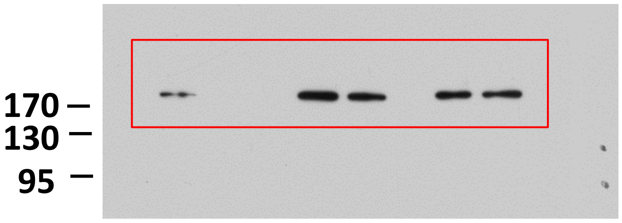

Vinculin

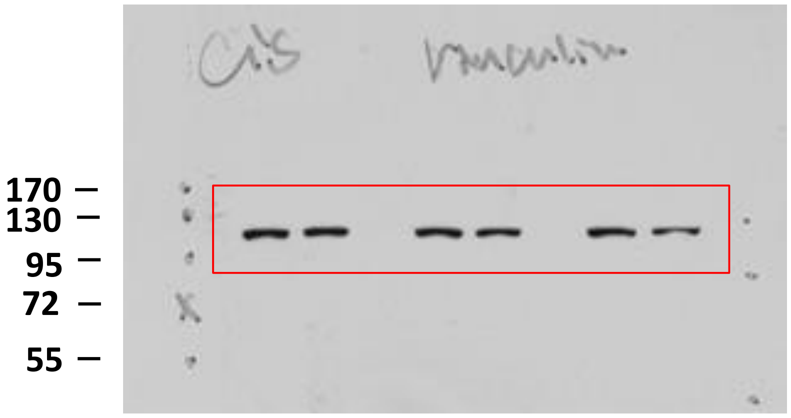

Fbw7

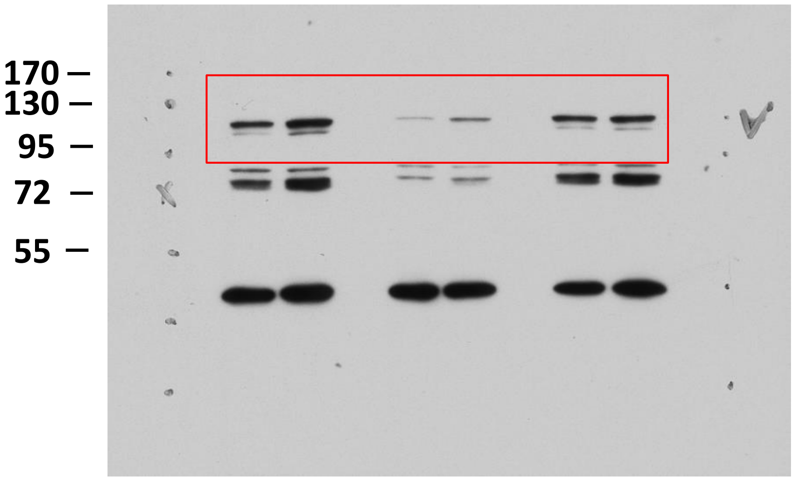

CK 1δ

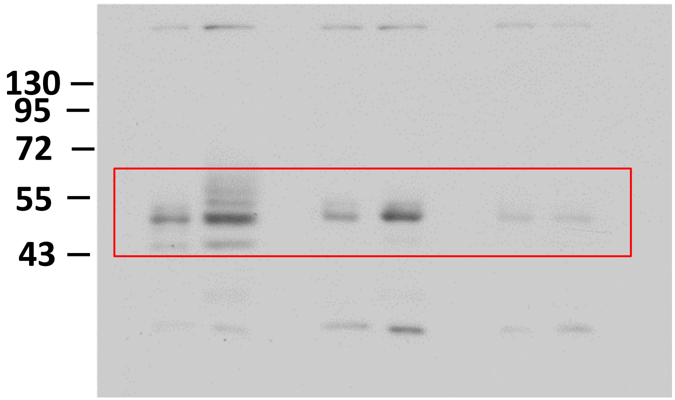

Uncropped blots related to Figure S4f, S4g

Fig S4f-Brg-1 (IP)

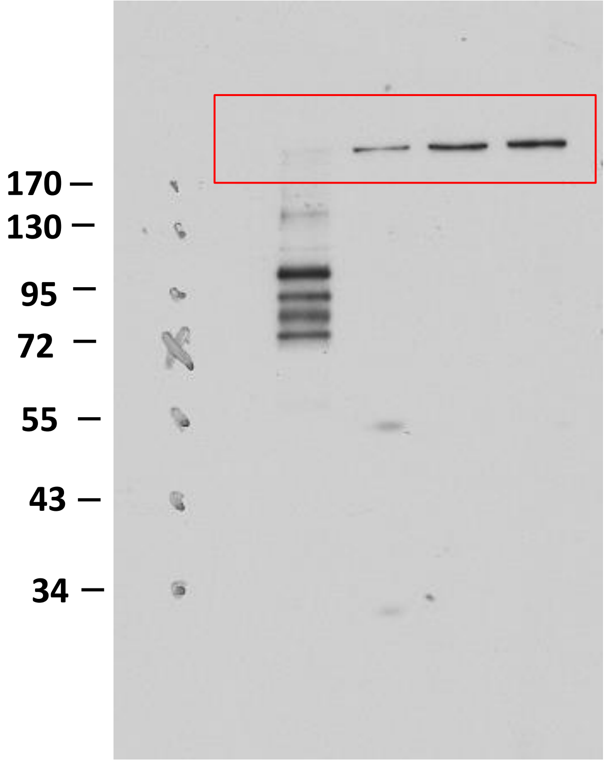

Fig S4g-Ub (IP)

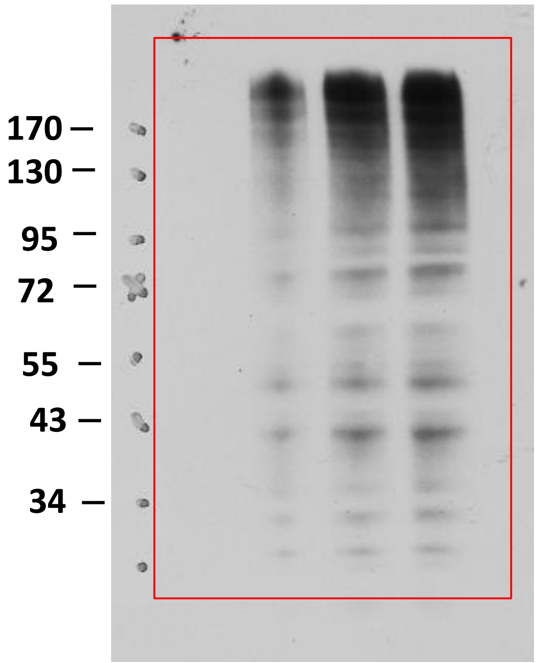

Fig S4f-Fbw7 (IP)

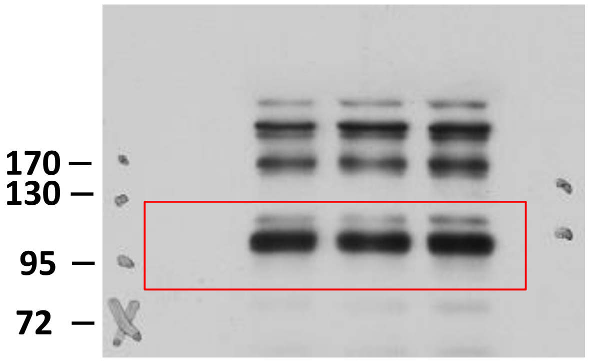

Fig S4g-Brg-1 (Lysates)

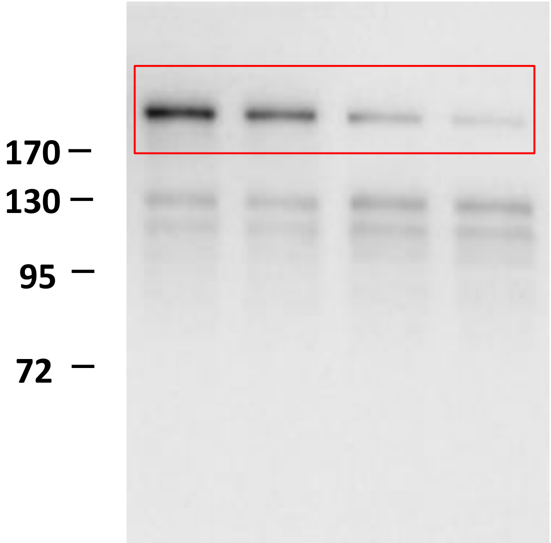

Fig S4g-Tubulin (Lysates)

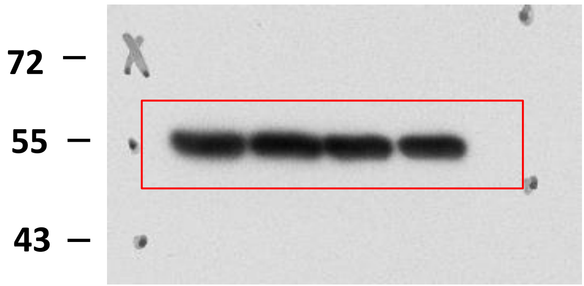

Uncropped blots related to Figure S9a, S9d

Fig S9a-c-JUN

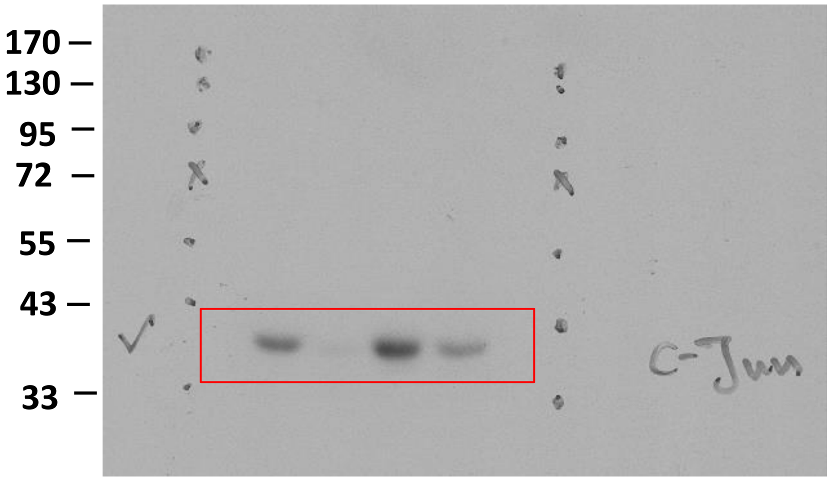

Fig S9a-FBW7

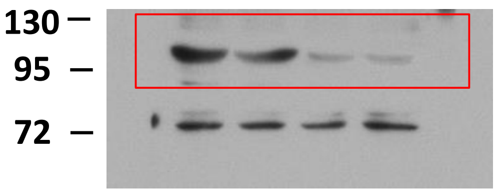

Fig S9a-Vinculin

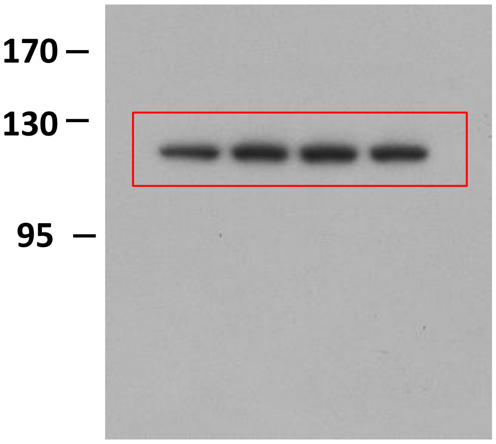

Fig S9d-KLF-2

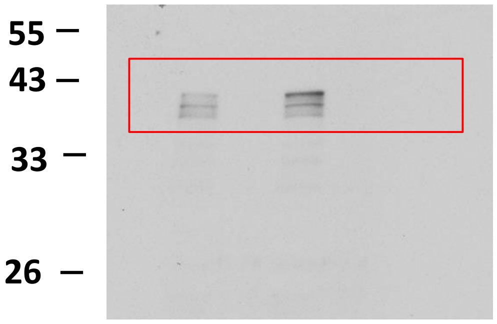

Fig S9d-FBW7

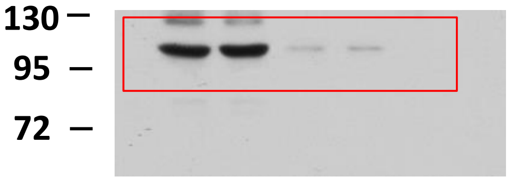

Fig S9d-Vinculin

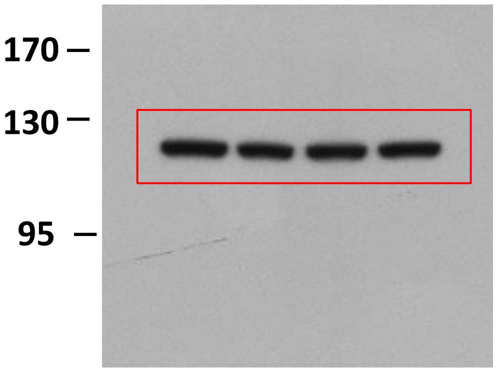

Uncropped blots related to Figure S11a

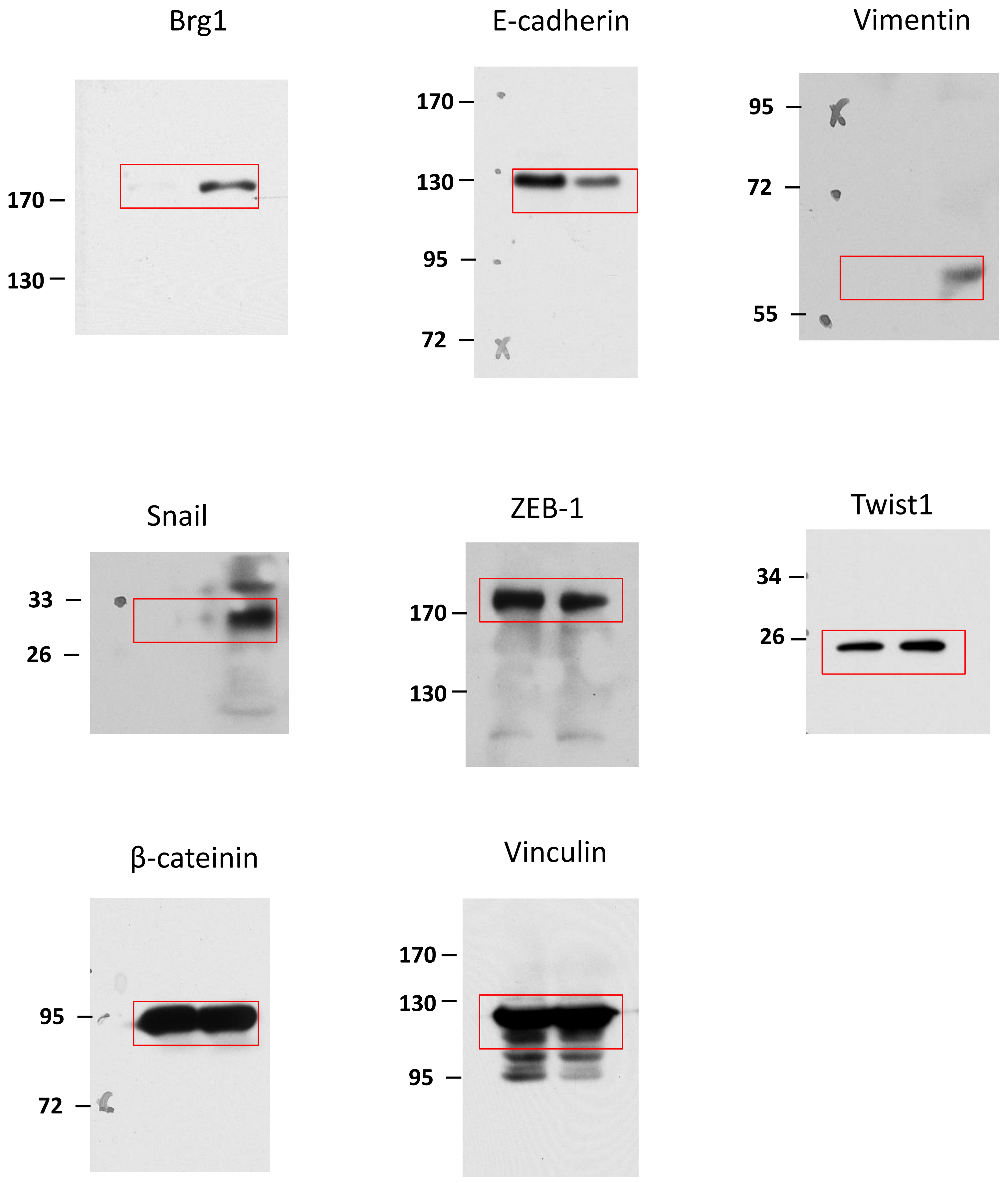

Uncropped blots related to Figure S11b

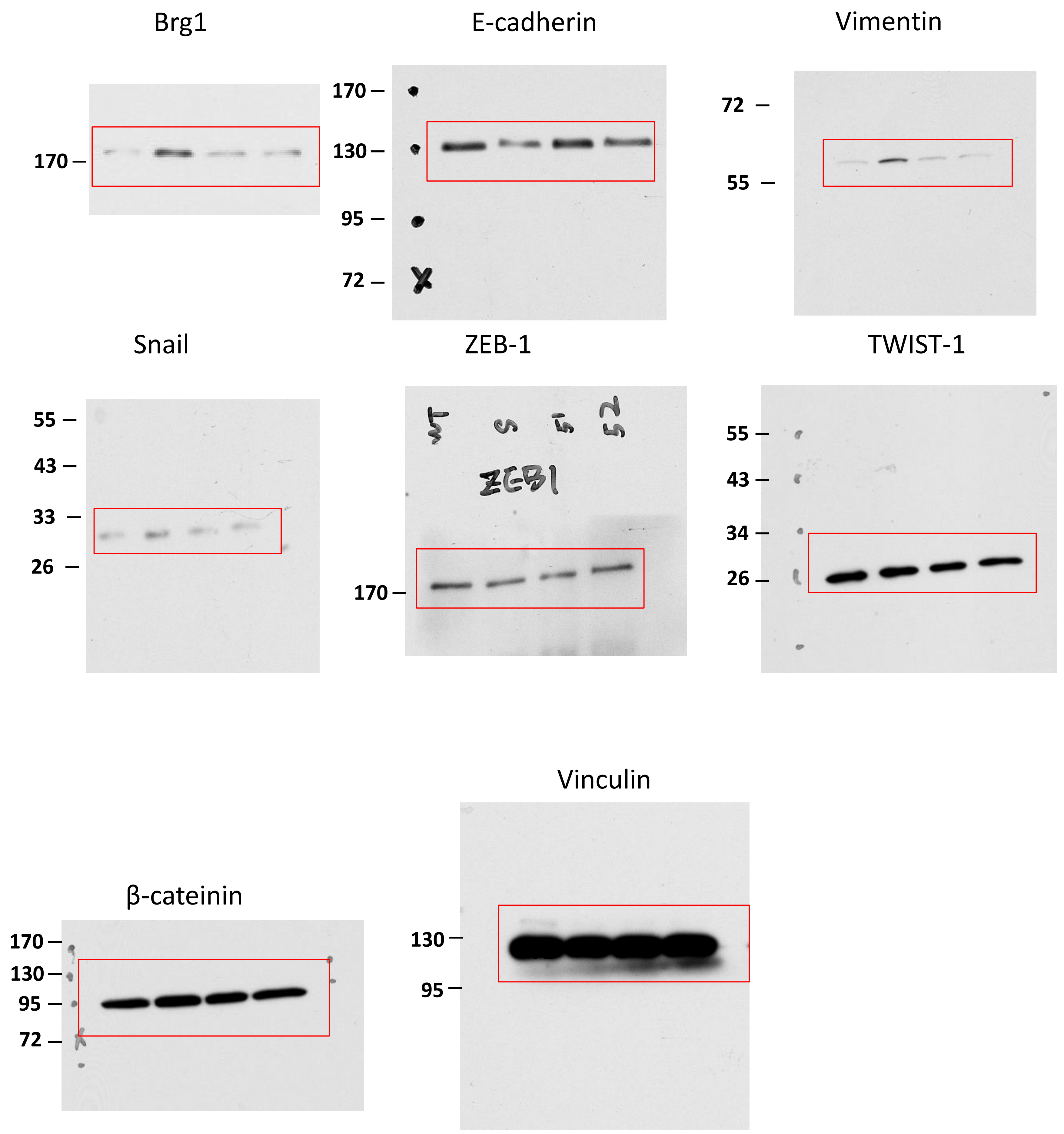

Uncropped blots related to Figure S11c

Brg1

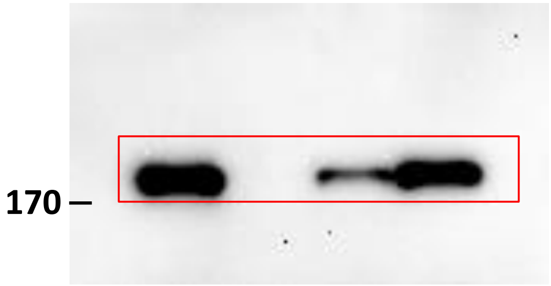

E-cadherin

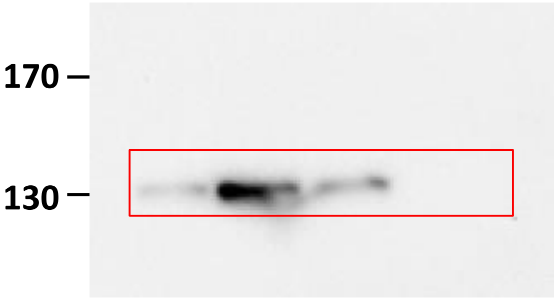

Vimentin

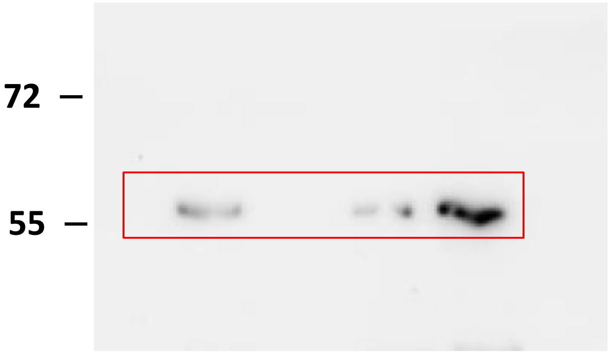

Snail

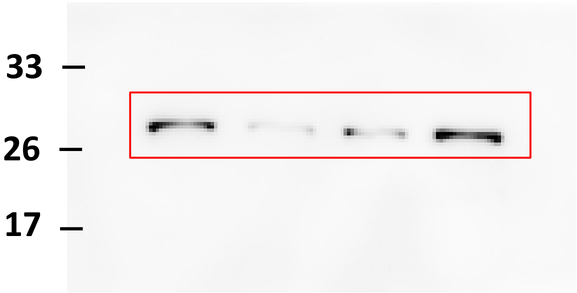

Vinculin

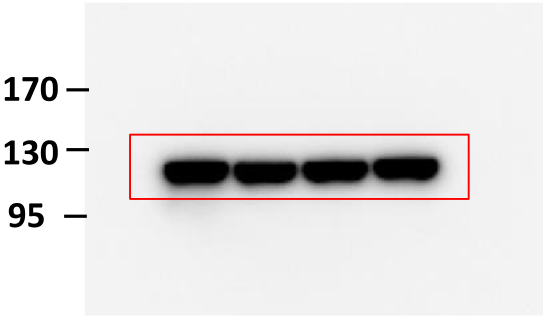

Uncropped blots related to Figure S12a

Flag-Brg1

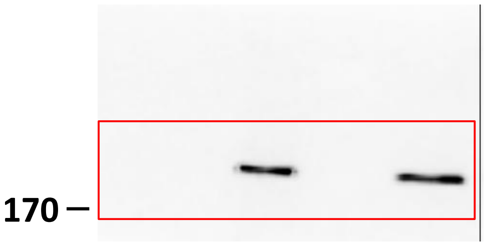

Snail

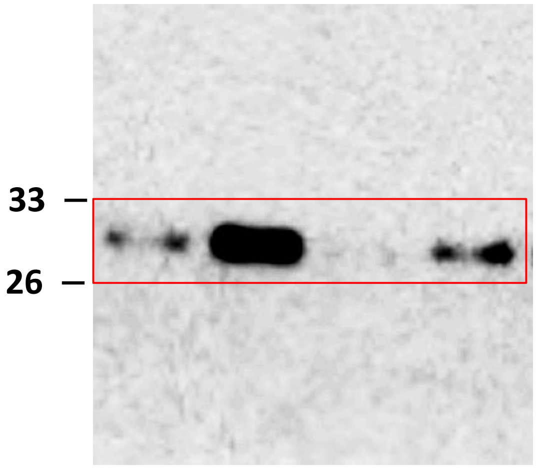

Vinculin

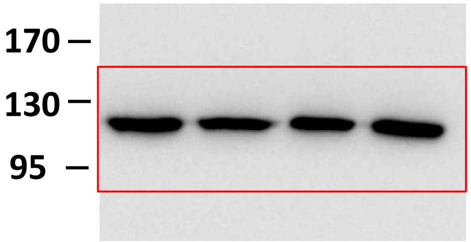

Uncropped blots related to Figure S13c

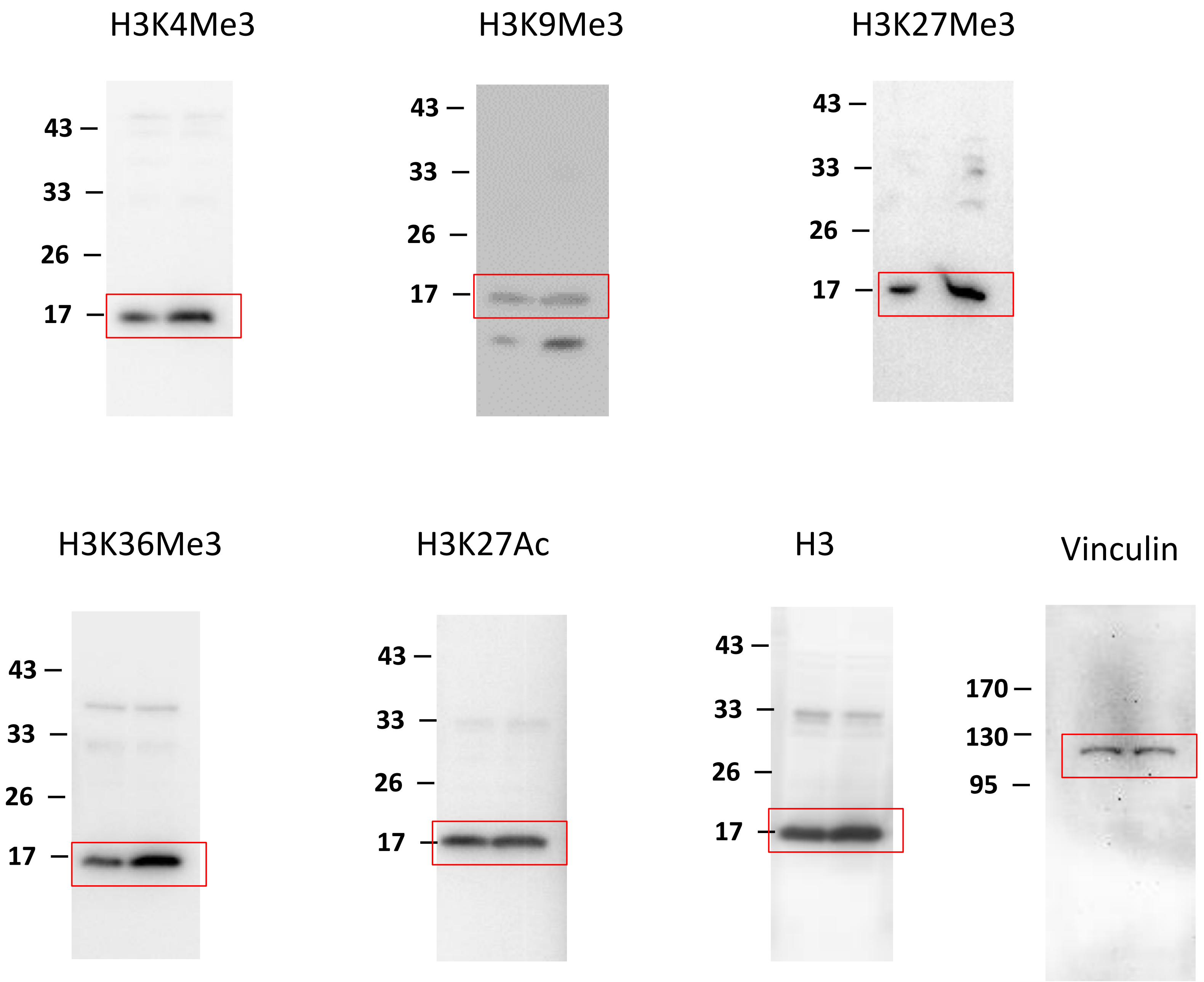

Supplement: Supplementary file 1 — Supplementary Information [file 41467_2018_6038_MOESM1_ESM.pdf]
